# An Effective Synthesis of Previously Unknown 7-Aryl Substituted Paullones

**DOI:** 10.3390/molecules28052324

**Published:** 2023-03-02

**Authors:** Dmitrii A. Aksenov, Alesia S. Akulova, Elena A. Aleksandrova, Nicolai A. Aksenov, Alexander V. Leontiev, Alexander V. Aksenov

**Affiliations:** Department of Chemistry, North Caucasus Federal University, 1a Pushkin St., 355009 Stavropol, Russia

**Keywords:** Fisher indolization, paullones synthesis, anticancer drug discovery

## Abstract

A straightforward three-step procedure affording a wide range of novel 7-aryl substituted paullone derivatives was developed. This scaffold is structurally similar to 2-(1*H*-indol-3-yl)acetamides—promising antitumor agents—hence, could be useful for the development of a new class of anticancer drugs.

## 1. Introduction

**Figure 1:**
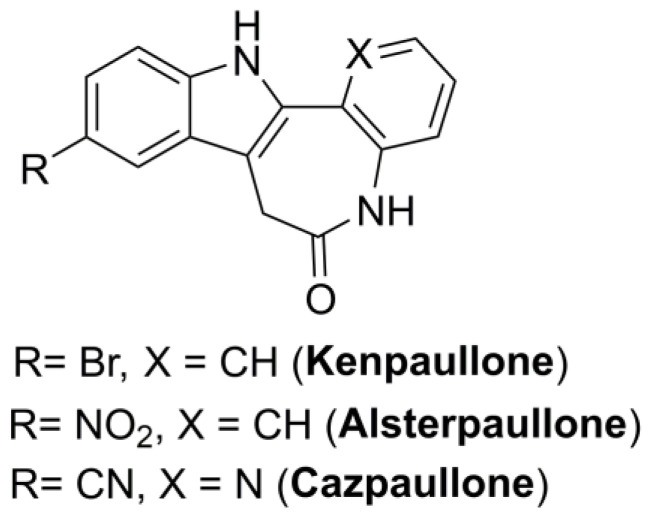
Some biologically active paullones.

Paullones are an important class of biologically active compounds that were found to be, for instance, efficient inhibitors of various kinases [1,2,3,4,5], including diazepam-binding sites [6] and agonists of GABA-A receptors [6]. Other types of activities shown by this kind of molecules are antitumor [3,7,8], anti-inflammatory [9], anti-parasitic [10], and antiviral [11] ones. Thus, recently, several members of the paullone family (Figure 1) were subjects of studies evaluating their applicability to treat some common illnesses and conditions, such as Alzheimer’s disease [2], osteoporosis [12], and leishmaniasis [13].

**Scheme 1:**
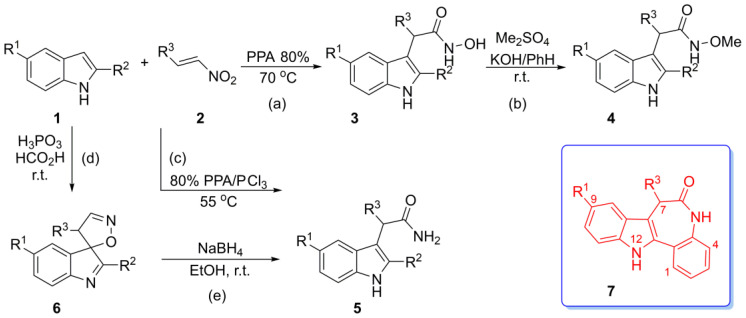
Indole hydroxamic acids **3** and indole amides **5** as structural analogs of 7-aryl substituted paullones **7**.

Although a large number of paullone derivatives are known, including 7-alkylidene [14] and 7-trifluoromethyl [15] ones, compounds possessing an aryl substituent at C-7 (**7**, Scheme 1) are somewhat underrepresented and, therefore, understudied. At the same time, in our opinion, such molecules should be of great interest, since they are structurally similar to indolyl-3-arylacetylhydroxamic acids **3**, which demonstrated very promising in vitro activity against cancer cells resistant to apoptosis, as well as against multi-drug resistant cell lines [16,17,18]. However, according to the initial in vivo tests, those hydroxamic acids **3**, while being readily accessible from indoles **1** and nitroalkanes **2** in polyphosphoric acid (PPA) (Scheme 1a) [16,19], have shown inefficiency in the remediation of cancer tumor in mice, supposedly, because of a highly unfavorable pharmacokinetic profile.

As it was found, the concentration of **3** in plasma goes below the activity threshold level within one hour, most likely due to the facile glucuronidation of hydroxamic acid functionality [17]. This result prompted us to search for the structural analogs of **3** that would be more resilient towards such metabolic degradation. Our first attempt involving protection of the hydroxamic acid moiety with the methoxy group proved to be unsuccessful because the obtained derivatives **4** demonstrated only marginal antitumor activity (Scheme 1b) [18]. Another approach relied upon the replacement of hydroxamic acid with primary amide function to obtain compounds **5**. To do so, two alternative synthetic pathways, both based on reactions of indoles **1** with nitrostyrenes **2**, were developed. According to the first one, the reaction takes place in a mixture of PPA (both as a solvent and reagent) and PCl_3_ (as a reductant) (Scheme 1c) [20]. The second method is a stepwise sequence where the intermediate spirane **6** (Scheme 1d) [21,22] formed in H_3_PO_3_ (a reaction media); then, it was reduced to the corresponding amide **5** with sodium borohydride (Scheme 1e) [23]. To our satisfaction, either approach works well and the obtained 2-(1*H*-indol-3-yl)acetamides **5** demonstrated in vitro submicromolar antitumor activity significantly exceeding the performance of hydroxamic acid analogs **3**. Supposedly, the structurally related cyclic amides, i.e., paullones **7** bearing aryl substituents R^3^ at C-7, would also possess the desired activity as well as required metabolic stability. Herein, we disclose the method for preparation of such compounds.

## 2. Results and Discussion

**Scheme 2:**
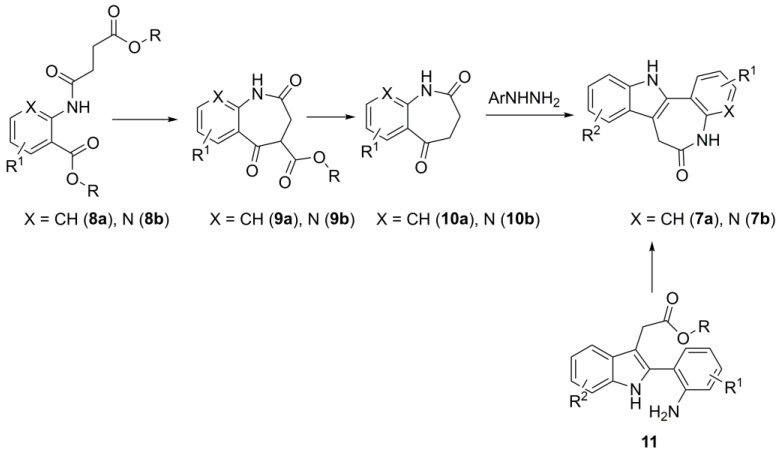
Common approaches to synthesis of the paullone derivatives.

Presently, there are many studies dedicated to the synthesis of various members of the paullone family. Most of them employ a rather standard approach of assembling the indole subunit via Fischer reaction [5,7,24,25,26,27,28,29] of arylhydrazines with a ketone **10** which, in turn, are accessed by the intramolecular condensation of anthranilic acids **8** following the decarboxylation of the resulting β-keto-acids **9** (Scheme 2). Several examples utilizing the lactonization of 2-(*o*-aminophenyl)indole-3-acetic esters **11** have also been reported [30,31,32,33,34].

**Scheme 3:**
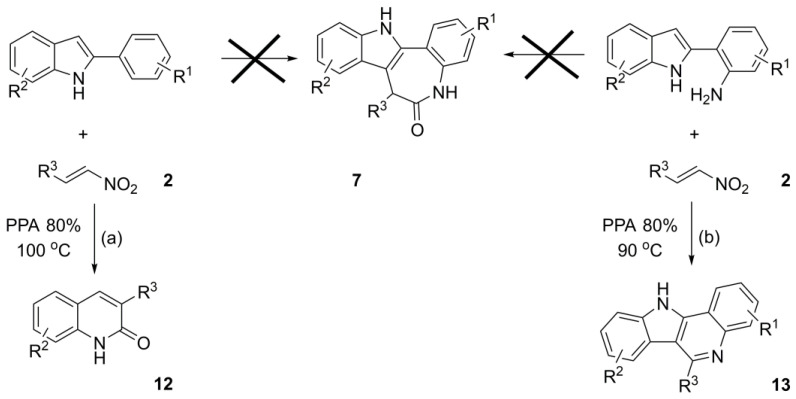
Overview of speculative approaches to paullones **7** based on the previously reported reactions of indoles with nitrostyrenes **2**.

We speculated that paullone derivatives **7** could be easily accessed as well via synthetic methodologies previously developed in our laboratories, such as the acetamidation of electron-rich arenes with nitroalkenes [35]. It was shown, however, that the reaction of the corresponding 2-arylindoles with nitrostyrenes **2** leads to the formation of 2-quinolones **12** instead of desired paullones **7** [19] (Scheme 3a). An attempt to introduce an *ortho*-amine function into the aryl substituent did not afford the paullones either, resulting in indoloquinolines **13** related to the isocryptolepine alkaloid as the only isolable products (Scheme 3b) [36]. This prompted us to explore alternative approaches based on the classical Fischer indolization reaction.

**Scheme 4:**
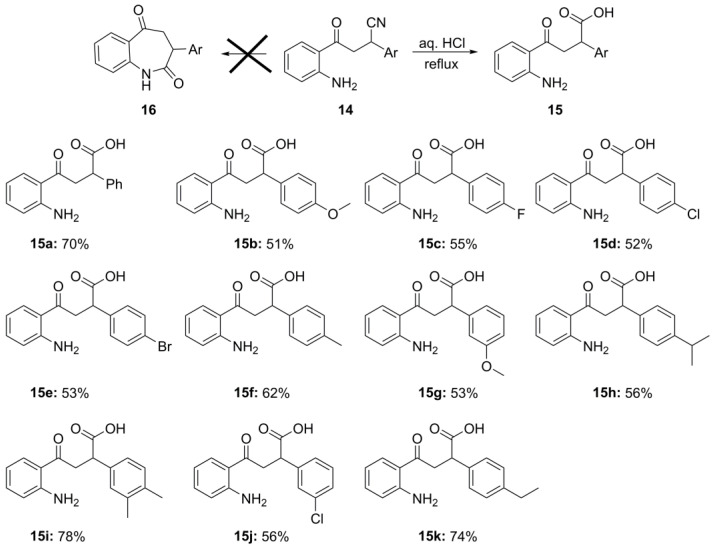
Preparation of the starting acids **15**.

To explore this venue, however, one would need an efficient route to suitable keto-lactam precursors **16**. Originally, we assumed that this could be achieved through the direct cyclization of readily available cyanoketones **14** [37] to keto-lactams **16**; however, none of the attempts to carry out this transformation were successful affording only black tar-like residue. Therefore, a two-step protocol, involving initial acid-mediated hydrolysis of the nitrile function, was implemented (Scheme 4) to give the expected acids **15** with yields ranging from 51 to 78%.

**Scheme 5:**
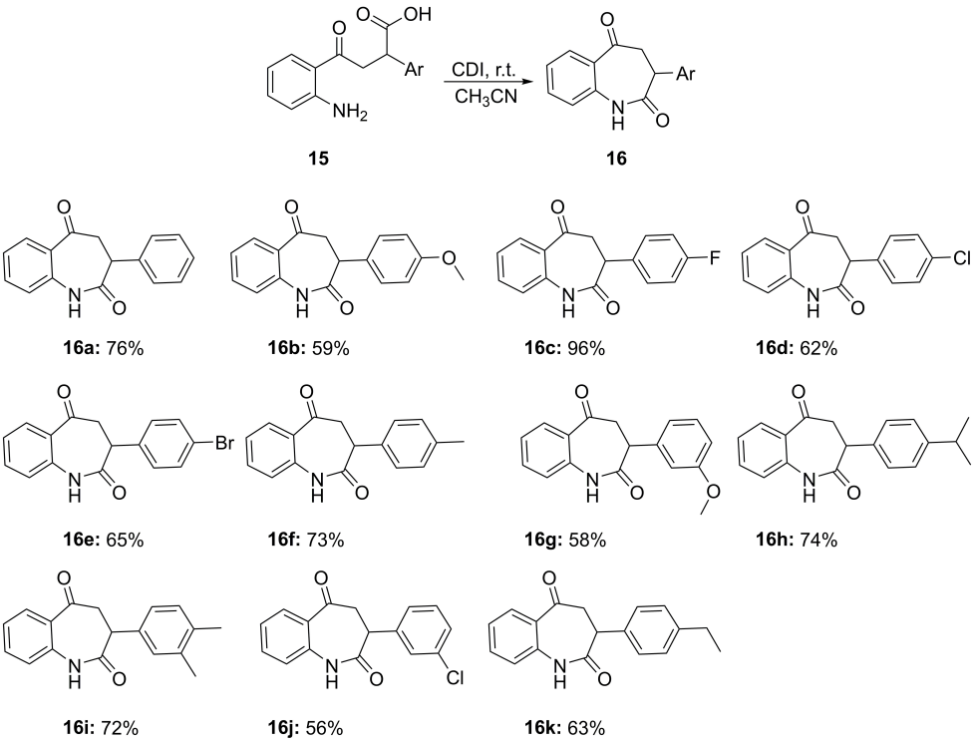
Intramolecular cyclization of acids **15** to keto-lactams **16**.

The next step, a cyclization of the obtained acid **15** to the corresponding lactam **16** was carried out in the presence of 1,1′-carbonyldiimidazole (CDI) in acetonitrile (Scheme 5).

Having access to a variety of keto-lactams **16**, we were ready to run the Fisher indolization only to find out, disappointingly, that this reaction does not proceed under the commonly used conditions. Therefore, screening studies of the reaction between keto-lactam **16a** and phenylhydrazine **17** under various conditions were undertaken (Table 1).

**Scheme 6:**
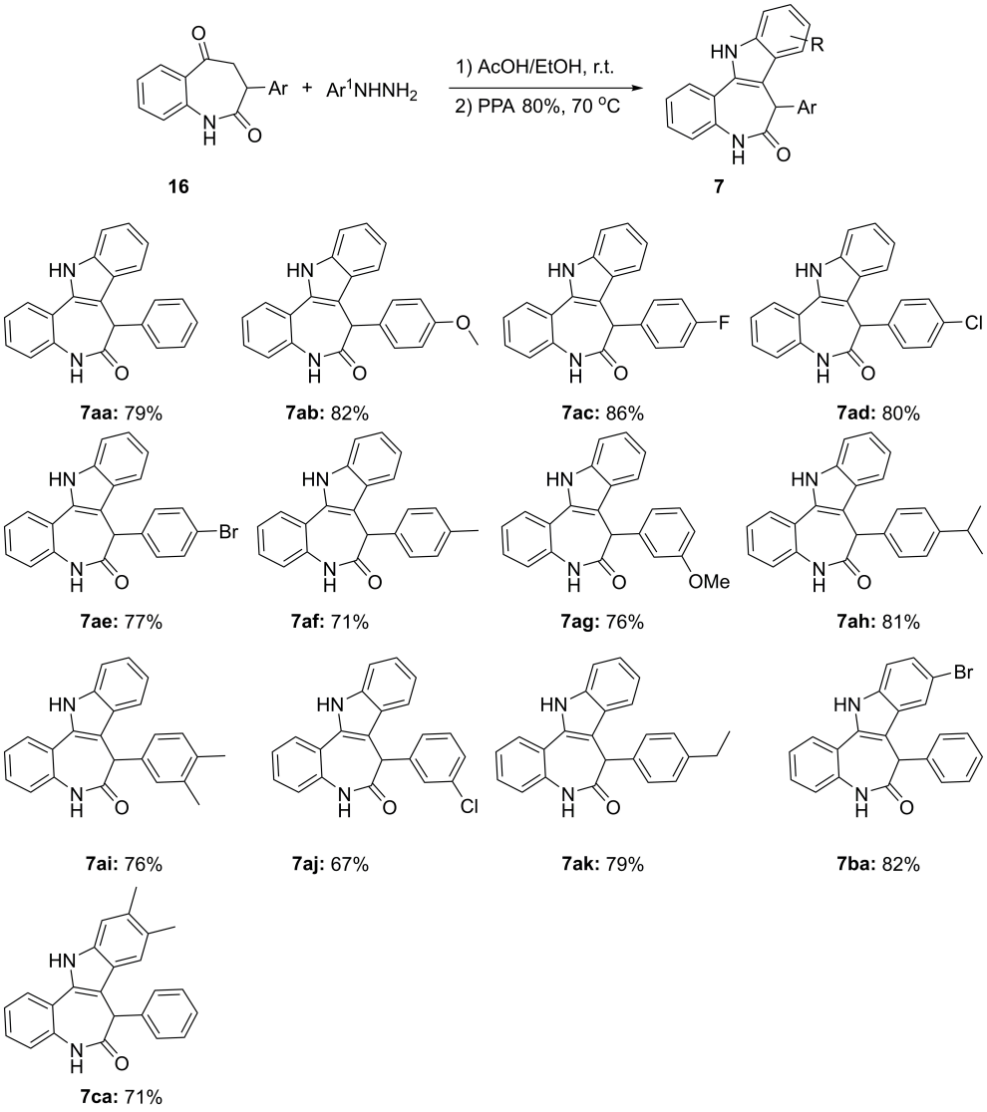
Library of 7-aryl paullones (**7**) synthesized by the procedure developed herein.

Thus, it was found that reaction in PPA containing 80% P_2_O_5_ produces only trace amounts of targeted paullone **7aa** (entries 1,2) and PPA with an increased concentration of P_2_O_5_ gives even worse results (entries 3,4). Similarly, an unsatisfactory outcome was observed with methanesulfonic acid (MsOH) (entry 5). Marginal improvement was achieved when a reaction in the presence of 80% PPA was carried out in organic solvent (EtOAc or EtOH), as the yields were increased to almost 30% (entries 6,7). At this point, it seemed evident that the root of the problem lies in the formation of the key intermediate—corresponding hydrazone—which is problematic in PPA for some reasons. This issue was addressed by carrying out the indolization reaction in two steps (entry 8). First, 3-phenyl-3,4-dihydro-1*H*-benzo[*b*]azepine-2,5-dion (**16a**) and phenylhydrazine (**17**) were allowed to react in ethanol at room temperature for 30 min in the presence of acetic acid (entry 8.1). Then PPA containing 80% P_2_O_5_ was added to the reaction mixture, which was then heated for another 30 min at 70 °C (entry 8.2). Under these conditions, the desired product **7aa** was obtained in 79% yield. Therefore, with the optimized conditions in hand, we proceeded with the synthesis of a series of paullones **7** (Scheme 6). As could be seen, the reaction is rather tolerant to the nature of the aryl substituent both in hydrazine and keto-lactam **16** as the yields stays in the range between 67 and 86%.

## 3. Experimental

### 3.1. General Information

NMR spectra (^1^H, ^13^C and ^19^F) were recorded on a Bruker AVANCE-III HD spectrometer (400, 101, and 376 MHz, respectively) equipped with a BBO probe in CDCl_3_ or DMSO-*d*_6_ using residual solvent signals as the internal standard. Spectral data are provided in the Supplementary Materials (Figures S1–S75). High-resolution mass spectra were registered in MeCN solutions on a Bruker maXis impact (electrospray ionization, using HCO_2_ Na–HCO_2_H for calibration). IR spectra were measured on a FT-IR spectrometer Shimadzu IRAffinity-1 S equipped with an ATR sampling module. Reaction progress, the purity of isolated compounds, and R_f_ values were assessed by TLC on Silufol UV-254 plates. Column chromatography was performed on silica gel (32–63 μm, 60 Å pore size). Melting points were measured on Stuart SMP30 apparatus. Cyanoketones **14a–d,f,h,j,k** [37] and **14e [38]** were synthesized according to the previously reported procedures and were identical to those described. All the reagents and solvents were purchased from commercial vendors and used as received.

### 3.2. General Procedure for Preparation of 4-(2-Aminophenyl)-2-aryl-4-oxo-butanenitriles ***14g,i***

These compounds were prepared in analogy to the method described in [37]. A 25 mL round-bottom flask was charged with 3-aryl-2′-aminochalcone (2.00 mmol), acetic acid (120 mg, 0.114 mL, 2.00 mmol), and DMSO (6 mL). The mixture was vigorously stirred, and a solution of KCN (260 mg, 4.00 mmol) in water (0.5 mL) was added dropwise. Then, the reaction vessel was equipped with a reflux condenser, and the mixture was stirred at 50 °C for 1 h, while the reaction progress was monitored by TLC. Upon complete conversion, the mixture was diluted with water (30 mL) and extracted with dichloromethane (4 × 15 mL). Combined organic extracts were washed with water (4 × 15 mL), concentrated in vacuum, and purified by preparative column chromatography on silica gel eluting with 1:4 EtOAc/hexane.

**4-(2-Aminophenyl)-2-(3-methoxyphenyl)-4-oxobutanenitrile (14g).** Orange solid, mp (EtOH) 94.9–96.5 °C, R*_f_* 0.63 (EtOAc/Hex, 1:2). Yield: 376 mg (1.40 mmol, 70%). ^1^H NMR (400 MHz, CDCl_3_) δ 7.59 (d, *J* = 8.2 Hz, 1H), 7.37–7.31 (m, 2H), 7.30–7.24 (m, 1H), 6.95–6.86 (m, 2H), 6.70–6.57 (m, 2H), 6.31 (s, 2H), 4.47 (dd, *J* = 8.4, 5.7 Hz, 1H), 3.81 (s, 3H), 3.66 (dd, *J* = 17.5, 8.4 Hz, 1H), 3.44 (dd, *J* = 17.5, 5.9 Hz, 1H). ^13^C NMR (101 MHz, CDCl_3_) δ 196.4, 159.6, 150.8, 135.1, 130.6, 128.8 (2C), 127.5, 121.4, 117.6, 116.9, 116.0, 114.7 (2C), 55.5, 45.1, 31.4. IR, v_max_/cm^−1^: 2939, 2255, 1676, 1646, 1238, 1539, 1622, 1236, 974. HRMS (ES TOF) calculated for [M + Na]^+^ C_17_H_16_ N_2_NaO_2_ 303.1104, found 303.1098 (−2.0 ppm).

**4-(2-Aminophenyl)-2-(3,4-dimethylphenyl)-4-oxobutanenitrile (14i).** Yellow solid, mp (EtOH) 137.3–139.7 °C, R*_f_* 0.39 (EtOAc/Hex, 1:2). Yield: 423 mg (1.52 mmol, 76%). ^1^H NMR (400 MHz, CDCl_3_) δ 7.59 (dd, *J* = 8.3, 1.5 Hz, 1H), 7.31–7.23 (m, 1H), 6.96 (dd, *J* = 8.2, 2.2 Hz, 1H), 6.91 (d, *J* = 2.2 Hz, 1H), 6.85 (d, *J* = 8.3 Hz, 1H), 6.66 (dd, *J* = 8.3, 1.1 Hz, 1H), 6.64–6.59 (m, 1H), 6.32 (s, 2H), 4.47 (dd, *J* = 8.4, 5.7 Hz, 1H), 3.90 (s, 3H), 3.87 (s, 3H), 3.67 (dd, *J* = 17.6, 8.4 Hz, 1H), 3.45 (dd, *J* = 17.5, 5.7 Hz, 1H). ^13^C NMR (101 MHz, CDCl_3_) δ 196.4, 150.8, 149.5, 149.0, 135.1, 130.6, 127.9, 121.3, 119.8, 117.6, 116.8, 116.0, 111.6, 110.6, 56.10, 56.08, 45.1, 31.8. IR, v_max_/cm^−1^: 2931, 2247, 1684, 1507, 1254, 1248, 1018. HRMS (ES TOF) calculated for [M + Na]^+^ C_18_H_18_N_2_NaO 301.1311, found 301.1319 (2.7 ppm).

### 3.3. General Procedure for Preparation of 4-(2-Aminophenyl)-4-oxo-2-arylbutanoic Acids ***15a–k***

A 5 mL round-bottom flask equipped with a magnetic stirring bar was charged with the corresponding 4-(2-aminophenyl)-4-oxo-2-arylbutanenitrile **14a–k** (2.00 mmol), concentrated aqueous HCl solution (2 mL), and refluxed for 2 h (TLC control). After the consumption of the starting material, the reaction mixture was cooled to room temperature, carefully neutralized with 10% solution of NaHCO_3,_ and then washed with EtOAc (5 × 20 mL). The combined organic layer was concentrated and purified by column chromatography (EtOAc:Hex), followed by recrystallization from EtOAc.

**4-(2-Aminophenyl)-4-oxo-2-phenylbutanoic acid (15a)**. Yellow solid, mp (EtOAc) 138.0–139.1 °C, R*_f_* 0.40 (EtOAc/Hex, 1:1). Yield: 376 mg (1.40 mmol, 70%). ^1^H NMR (400 MHz, DMSO-*d*_6_) δ 12.31 (s, 1H), 7.84–7.81 (m, 1H), 7.39–7.32 (m, 4H), 7.29–7.21 (m, 2H), 7.18 (s, 2H), 6.77–6.72 (m, 1H), 6.55–6.48 (m, 1H), 4.06 (dd, *J* = 10.8, 3.7 Hz, 1H), 3.80 (dd, *J* = 17.9, 10.8 Hz, 1H), 3.20 (dd, *J* = 17.7, 3.8 Hz, 1H). ^13^C NMR (101 MHz, DMSO-*d*_6_) δ 199.5, 174.5, 151.1, 139.2, 134.2, 131.3, 128.6 (2C), 128.0 (2C), 127.1, 116.9, 116.2, 114.4, 46.2, 42.5. IR, v_max_/cm^−1^: 3507, 3380, 2915, 2362, 1646, 1622, 1539, 1236, 974. HRMS (ES TOF) calculated for [M + Na]^+^ C_16_H_15_NNaO_3_ 292.0944, found 292.0950 (2.1 ppm).

**4-(2-Aminophenyl)-2-(4-methoxyphenyl)-4-oxobutanoic acid (15b)**. Yellow solid, mp (EtOAc) 192.3–194.3 °C, R*_f_* 0.27 (EtOAc/Hex, 1:1). Yield: 304 mg (1.02 mmol, 51%). ^1^H NMR (400 MHz, DMSO-*d*_6_) δ 12.24 (s, 1H), 7.82 (d, *J* = 8.0 Hz, 1H), 7.29 (d, *J* = 8.4 Hz, 2H), 7.23 (t, *J* = 7.6 Hz, 1H), 7.18 (s, 2H), 6.90 (d, *J* = 8.2 Hz, 2H), 6.74 (d, *J* = 8.3 Hz, 1H), 6.56–6.47 (m, 1H), 4.00 (dd, *J* = 10.5, 3.7 Hz, 1H), 3.78 (dd, *J* = 17.7 Hz, 1H), 3.73 (s, 3H), 3.15 (dd, *J* = 17.7, 3.9 Hz, 1H). ^13^C NMR (101 MHz, DMSO-*d*_6_) δ 199.6, 174.8, 158.3, 151.1, 134.2, 131.3, 131.1, 129.0 (2C), 116.9, 116.3, 114.4, 114.0 (2C), 55.1, 45.3, 42.5. IR, v_max_/cm^−1^: 3507, 3384, 2990, 2366, 1769, 1238, 827, 743. HRMS (ES TOF) calculated for [M + Na]^+^ C_17_H_17_NNaO_4_ 322.1050, found 322.1059 (2.8 ppm).

**4-(2-Aminophenyl)-2-(4-fluorophenyl)-4-oxobutanoic acid (15c)**. Yellow solid, mp (EtOAc) 157.8–158.9 °C, R*_f_* 0.31 (EtOAc/Hex, 1:1). Yield: 315 mg (1.10 mmol, 55%). ^1^H NMR (400 MHz, DMSO-*d*_6_) δ 12.38 (s, 1H), 7.83 (d, *J* = 7.7 Hz, 1H), 7.45–7.40 (m, 2H), 7.25–7.16 (m, 4H), 7.15 (s, 1H), 6.74 (d, *J* = 8.3 Hz, 1H), 6.52 (t, *J* = 7.5 Hz, 1H), 4.08 (dd, *J* = 10.6, 3.9 Hz, 1H), 3.80 (dd, *J* = 17.8, 10.6 Hz, 1H), 3.20 (dd, *J* = 17.8, 4.0 Hz, 1H). ^13^C NMR (101 MHz, DMSO-*d*_6_) δ 199.4, 174.4, 161.3 (d, *J* = 242.8 Hz), 151.1, 135.4 (d, *J* = 2.9 Hz), 134.3, 131.3, 129.9 (d, *J* = 8.1 Hz, 2C), 116.9, 116.2, 115.3 (d, *J* = 21.3 Hz, 2C), 114.4, 45.4, 42.4. ^19^F NMR (376 MHz, DMSO-*d*_6_) δ −115.9. IR, v_max_/cm^−1^: 3460, 2919, 2358, 1765, 1507, 1236, 978, 739. HRMS (ES TOF) calculated for [M + Na]^+^ C_16_H_14_FNNaO_3_ 310.0850, found 310.0842 (2.6 ppm).

**4-(2-Aminophenyl)-2-(4-chlorophenyl)-4-oxobutanoic acid (15d)**. Yellow solid, mp (EtOAc) 191.1–192.4 °C, R*_f_* 0.37 (EtOAc/Hex, 1:1). Yield: 315 mg (1.04 mmol, 52%). ^1^H NMR (400 MHz, DMSO-*d*_6_) δ 12.44 (s, 1H), 7.82 (d, *J* = 7.6 Hz, 1H), 7.40 (s, 4H), 7.26–7.22 (m, 1H), 7.18 (s, 2H), 6.74 (d, *J* = 8.2 Hz, 1H), 6.52 (t, *J* = 7.5 Hz, 1H), 4.08 (dd, *J* = 10.5, 4.0 Hz, 1H), 3.78 (dd, *J* = 17.9, 10.5 Hz, 1H), 3.22 (dd, *J* = 17.8, 4.1 Hz, 1H). ^13^C NMR (101 MHz, DMSO-*d*_6_) δ 199.3, 174.2, 151.1, 138.3, 134.3, 131.7, 131.3, 129.9 (2C), 128.5 (2C), 116.9, 116.2, 114.4, 45.6, 42.2. IR, v_max_/cm^−1^: 3519, 3376, 3002, 2358, 1773, 1244, 820, 749. HRMS (ES TOF) calculated for [M + Na]^+^ C_16_H_14_ClNNaO_3_ 326.0554, found 326.0562 (2.6 ppm).

**4-(2-Aminophenyl)-2-(4-bromophenyl)-4-oxobutanoic acid (15e)**. Yellow solid, mp (EtOAc) 196.5–197.8 °C, R*_f_* 0.33 (EtOAc/Hex, 1:1). Yield: 298 mg (0.86 mmol, 43%). ^1^H NMR (400 MHz, DMSO-*d*_6_) δ 12.43 (s, 1H), 7.83–7.81 (m, 1H), 7.56–7.52 (m, 2H), 7.36–7.33 (m, 2H), 7.26–7.22 (m, 1H), 7.18 (s, 2H), 6.75–6.73 (m, 1H), 6.54–6.50 (m, 1H), 4.06 (dd, *J* = 10.5, 4.0 Hz, 1H), 3.78 (dd, *J* = 17.8, 10.5 Hz, 1H), 3.22 (dd, *J* = 17.7, 4.0 Hz, 1H). ^13^C NMR (101 MHz, DMSO-*d*_6_) δ 199.3, 174.1, 151.1, 139.0, 134.2, 131.4 (2C), 131.3, 130.3 (2C), 120.1, 116.9, 116.2, 114.4, 45.9, 42.2. IR, v_max_/cm^−1^: 3360, 2994, 2358, 1771, 1244, 1053, 819, 751. HRMS (ES TOF) calculated for [M + Na]^+^ C_16_H_14_BrNNaO_3_ 370.0049, found 370.0038 (−3.0 ppm).

**4-(2-Aminophenyl)-4-oxo-2-(*p*-tolyl)butanoic acid (15f)**. Colorless solid, mp (EtOAc) 153.7–155.2 °C, R*_f_* 0.34 (EtOAc/Hex, 1:1). Yield: 350 mg (1.24 mmol, 62%). ^1^H NMR (400 MHz, CDCl_3_) δ 7.72 (d, *J* = 8.1 Hz, 1H), 7.30–7.20 (m, 4H), 7.15 (d, *J* = 8.1 Hz, 2H), 6.66–6.57 (m, 2H), 4.21 (dd, *J* = 10.4, 3.9 Hz, 1H), 3.88 (dd, *J* = 17.8, 10.5 Hz, 1H), 3.27 (dd, *J* = 17.8, 4.0 Hz, 1H), 2.33 (s, 3H).^13^C NMR (101 MHz, CDCl_3_) δ 199.4, 178.8, 150.5, 137.6, 134.9, 134.7, 131.0, 129.7 (2C), 128.0 (2C), 117.5, 115.9, 46.1, 43.1, 21.2. IR, v_max_/cm^−1^: 3380, 2994, 1773, 1684, 1656, 1507, 1475, 1437, 1240. HRMS (ES TOF) calculated for [M + Na]^+^ C_17_H_17_NNaO_3_ 306.1101, found 306.1088 (4.3 ppm).

**4-(2-Aminophenyl)-2-(3-methoxyphenyl)-4-oxobutanoic acid (15g)**. Colorless solid, mp (EtOAc) 163.9–165.1 °C, R*_f_* 0.26 (EtOAc/Hex, 1:1). Yield: 316 mg (1.06 mmol, 53%). ^1^H NMR (400 MHz, DMSO-*d*_6_) δ 12.22 (s, 1H), 7.82 (dd, *J* = 8.3, 1.5 Hz, 1H), 7.32–7.25 (m, 2H), 7.28–7.19 (m, 1H), 7.16 (d, *J* = 13.6 Hz, 2H), 6.94–6.86 (m, 2H), 6.74 (dd, *J* = 8.4, 1.2 Hz, 1H), 6.52 (ddd, *J* = 8.2, 7.0, 1.2 Hz, 1H), 4.00 (dd, *J* = 10.8, 3.9 Hz, 1H), 3.81–3.74 (m, 1H), 3.73 (s, 3H), 3.15 (dd, *J* = 17.8, 3.8 Hz, 1H). ^13^C NMR (101 MHz, DMSO-*d*_6_) δ 199.6, 174.7, 158.3, 151.1, 134.2, 131.3, 131.1, 129.0 (2C), 116.9, 116.2, 114.4, 114.0 (2C), 55.1, 45.3, 42.5. IR, v_max_/cm^−1^: 3392, 2994, 1775, 1735, 1698, 1654, 1558, 1453, 1244. HRMS (ES TOF) calculated for [M + Na]^+^ C_17_H_17_NNaO_4_ 322.1050, found 322.1055 (1.6 ppm).

**4-(2-Aminophenyl)-2-(4-isopropylphenyl)-4-oxobutanoic acid (15h)**. Colorless solid, mp (EtOAc) 121.1–122.9 °C, R*_f_* 0.6 (EtOAc/Hex, 1:1). Yield: 348 mg (1.12 mmol, 56%). ^1^H NMR (400 MHz, DMSO-*d*_6_) δ 7.82 (d, *J* = 6.7 Hz, 1H), 7.29 (s, 2H), 7.24–7.16 (m, 5 H), 6.74 (d, *J* = 7.2 Hz, 1H), 6.51 (t, *J* = 7.6 Hz, 1H), 4.01 (dd, *J* = 10.8, 3.7 Hz, 1H), 3.78 (dd, *J* = 17.8, 10.8 Hz, 1H), 3.17 (dd, *J* = 17.8, 3.7 Hz, 1H), 2.86 (p, *J* = 6.9 Hz, 1H), 1.19 (d, *J* = 7.0 Hz, 6 H). ^13^C NMR (101 MHz, DMSO-*d*_6_) δ 199.6, 174.6, 151.06, 147.2, 136.6, 134.2, 131.3, 127.9 (2C), 126.5 (2C), 116.9, 116.2, 114.4, 45.8, 42.5, 33.1, 24.0 (2C). IR, v_max_/cm^−1^: 3280, 2954, 1712, 1668, 1556, 1514, 1442, 1427, 1251, 1062. HRMS (ES TOF) calculated for [M + Na]^+^ C_19_H_21_NNaO_3_ 334.1414, found 334.1422 (4.1 ppm).

**4-(2-Aminophenyl)-2-(3,4-dimethylphenyl)-4-oxobutanoic acid (15i)**. Yellowish solid, mp (EtOAc) 138.0–139.8 °C, R*_f_* 0.42 (EtOAc/Hex, 1:1). Yield: 439 mg (1.48 mmol, 74%). ^1^H NMR (400 MHz, DMSO-*d*_6_) δ 12.25 (s, 1H), 7.82 (dd, *J* = 8.2, 1.6 Hz, 1H), 7.30–7.25 (m, 2H), 7.24–7.21 (m, 1H), 7.20–7.15 (m, 3H), 6.74 (dd, *J* = 8.5, 1.2 Hz, 1H), 6.51 (t, *J* = 7.5 Hz, 1H), 4.02 (dd, *J* = 10.7, 3.6 Hz, 1H), 3.78 (dd, *J* = 17.8, 10.8 Hz, 1H), 3.17 (dd, *J* = 17.8, 3.8 Hz, 1H), 2.57 (q, *J* = 7.6 Hz, 2H), 1.20–1.14 (m, 3H). ^13^C NMR (101 MHz, DMSO-*d*_6_) δ 199.5, 174.6, 151.1, 142.6, 136.4, 134.2, 131.3, 128.0 (2C), 127.9 (2C), 116.9, 116.2, 114.4, 45.8, 42.5, 27.8, 15.7. IR, v_max_/cm^−1^: 3380, 2974, 1702, 1688, 1650, 1558, 1503, 1455, 1234, 1189. HRMS (ES TOF) calculated for [M + Na]^+^ C_18_H_19_NNaO_3_ 320.1257, found 320.1248 (−2.8 ppm).

**4-(2-Aminophenyl)-2-(3-chlorophenyl)-4-oxobutanoic acid (15j)**. Colorless solid, mp (EtOAc) 164.0–165.3 °C, R*_f_* 0.42 (EtOAc/Hex, 1:1). Yield: 339 mg (1.12 mmol, 56%). ^1^H NMR (400 MHz, CDCl_3_) δ 7.74 (d, *J* = 7.1 Hz, 1H), 7.40 (s, 1H), 7.29 (d, *J* = 6.7 Hz, H), 6.65 (d, *J* = 7.4 Hz, 2H), 4.25 (dd, *J* = 10.4, 4.0 Hz, 1H), 3.89 (dd, *J* = 17.8, 10.3 Hz, 1H), 3.32 (dd, *J* = 17.7, 4.0 Hz, 1H). ^13^C NMR (101 MHz, CDCl_3_) 198.8, 177.4, 150.6, 139.8, 134.9, 134.8, 131.0, 130.3, 128.3, 128.2, 126.5, 117.5, 117.3, 116.0, 46.1, 42.9. IR, v_max_/cm^−1^: 3002, 1775, 1759, 1708, 1618, 1562, 1435, 1240, 1053. HRMS (ES TOF) calculated for [M + Na]^+^ C_17_H_14_ClNNaO_3_ 326.0554, found 306.0541 (4.1 ppm).

**4-(2-Aminophenyl)-2-(4-ethylphenyl)-4-oxobutanoic acid (15k)**. Yellowish solid, mp (EtOAc) 138.0–139.8 °C, R*_f_* 0.42 (EtOAc/Hex, 1:1). Yield: 439 mg (1.48 mmol, 74%). ^1^H NMR (400 MHz, DMSO-*d*_6_) δ 12.25 (s, 1H), 7.82 (dd, *J* = 8.2, 1.6 Hz, 1H), 7.30–7.25 (m, 2H), 7.24–7.21 (m, 1H), 7.20–7.15 (m, 3H), 6.74 (dd, *J* = 8.5, 1.2 Hz, 1H), 6.51 (t, *J* = 7.5 Hz, 1H), 4.02 (dd, *J* = 10.7, 3.6 Hz, 1H), 3.78 (dd, *J* = 17.8, 10.8 Hz, 1H), 3.17 (dd, *J* = 17.8, 3.8 Hz, 1H), 2.57 (q, *J* = 7.6 Hz, 2H), 1.20–1.14 (m, 3H). ^13^C NMR (101 MHz, DMSO-*d*_6_) δ 199.5, 174.6, 151.1, 142.6, 136.4, 134.2, 131.3, 128.0 (2C), 127.9 (2C), 116.9, 116.2, 114.4, 45.8, 42.5, 27.8, 15.7. IR, v_max_/cm^−1^: 3380, 2974, 1702, 1688, 1650, 1558, 1503, 1455, 1234, 1189. HRMS (ES TOF) calculated for [M + Na]^+^ C_18_H_19_NNaO_3_ 320.1257, found 320.1248 (−2.8 ppm).

### 3.4. General Procedure for Preparation of 3-Aryl-3,4-dihydro-1H-benzo[b]azepine-2,5-diones ***16a–k***

A 5 mL round-bottom flask equipped with a magnetic stirring bar was charged with the corresponding 4-(2-aminophenyl)-4-oxo-2-arylbutanoic acid **15a–k** (1.00 mmol), 1,1′-carbonyldiimidazole (162 mg, 1.00 mmol), and acetonitrile (0.1 mL). The reaction mixture was left stirring at room temperature for 4 h. After this period of time, 20 mL of water was poured into the reaction mixture and then, extracted with EtOAc (5 × 20 mL). The organic phase was concentrated on a rotary evaporator and residue was purified by column chromatography using mixture EtOAc/Hex.

**3-Phenyl-3,4-dihydro-1*H*-benzo[b]azepine-2,5-dione (16a)**. Colorless solid, mp (EtOAc) 180.3–181.4 °C, R*_f_* 0.48 (EtOAc/Hex, 1:1). Yield: 190 mg (0.76 mmol, 76%). ^1^H NMR (400 MHz, DMSO-*d*_6_) δ 10.34 (s, 1H), 7.82–7.80 (m, 1H), 7.59–7.55 (m, 1H), 7.31–7.27 (m, 2H), 7.24–7.16 (m, 5 H), 4.29–4.25 (m, 1H), 3.60 (dd, *J* = 18.0, 12.0 Hz, 1H), 3.02–2.97 (m, 1H). ^13^C NMR (101 MHz, DMSO-*d*_6_) δ 197.8, 173.5, 139.0, 138.3, 134.4, 130.0, 128.7 (2C), 128.1 (2C), 126.9, 126.7, 123.6, 121.9, 45.8, 43.2. IR, v_max_/cm^−1^: 3312, 2919, 2366, 1670, 1485, 1250. HRMS (ES TOF) calculated for [M + Na]^+^ C_16_H_13_NNaO_2_ 274.0838, found 274.0843 (1.8 ppm).

**3-(4-Methoxyphenyl)-3,4-dihydro-1*H*-benzo[b]azepine-2,5-dione (16b)**. Colorless solid, mp (EtOAc) 174.8–176.2 °C, R*_f_* 0.32 (EtOAc/Hex, 1:1). Yield: 166 mg (0.59 mmol, 59%). ^1^H NMR (400 MHz, CDCl_3_) δ 8.23 (s, 1H), 7.99 (d, *J* = 7.9 Hz, 1H), 7.51 (t, *J* = 7.6 Hz, 1H), 7.23 (t, *J* = 7.6 Hz, 1H), 7.12 (d, *J* = 8.7 Hz, 2H), 6.96 (d, *J* = 8.1 Hz, 1H), 6.87 (d, *J* = 8.1 Hz, 2H), 4.16 (d, *J* = 11.5 Hz, 1H), 3.79 (s, 3H), 3.53–3.45 (m, 1H), 3.20 (d, *J* = 18.2 Hz, 1H). ^13^C NMR (101 MHz, CDCl_3_) δ 197.8, 174.7, 159.0, 137.7, 134.8, 131.1, 129.5 (2C), 128.7, 127.5, 125.0, 121.7, 114.2 (2C), 55.4, 46.6, 43.4. IR, v_max_/cm^−1^: 2923, 2374, 1771, 1662, 1514, 1248, 1027, 843, 767. HRMS (ES TOF) calculated for [M + Na]^+^ C_17_H_15_NNaO_3_ 304.0944, found 304.0944 (−1.6 ppm).

**3-(4-Fluorophenyl)-3,4-dihydro-1*H*-benzo[b]azepine-2,5-dione (16c)**. Colorless solid, mp (EtOAc) 212.5–214.6 °C, R*_f_* 0.34 (EtOAc/Hex, 1:1). Yield: 258 mg (0.96 mmol, 96%). ^1^H NMR (400 MHz, DMSO-*d*_6_) δ 10.34 (s, 1H), 7.83–7.80 (m, 1H), 7.60–7.56 (m, 1H), 7.30–7.25 (m, 2H), 7.22–7.19 (m, 2H), 7.16–7.10 (m, 2H), 4.34–4.30 (m, 1H), 3.61 (dd, *J* = 18.2, 12.4 Hz, 1H), 2.96–2.91 (m, 1H). ^13^C NMR (101 MHz, DMSO-*d*_6_) δ 197.9, 173.3, 161.2 (d, *J* = 242.5 Hz), 138.9, 134.7 (d, *J* = 3.3 Hz), 134.4, 130.8 (d, *J* = 8.4 Hz, 2C), 130.1, 126.8, 123.7, 121.9, 114.8 (d, *J* = 21.3 Hz, 2C), 46.1, 42.2. ^19^F NMR (376 MHz, DMSO-*d*_6_) δ −116.1. IR, v_max_/cm^−1^: 3285, 2915, 2334, 1779, 1652, 1376, 1246. HRMS (ES TOF) calculated for [M + Na]^+^ C_16_H_12_FNNaO_2_ 292.0744, found 292.0747 (−1.1 ppm).

**3-(4-Chlorophenyl)-3,4-dihydro-1*H*-benzo[b]azepine-2,5-dione (16d)**. Colorless solid, mp (EtOAc) 191.8–193.4 °C, R*_f_* 0.33 (EtOAc/Hex, 1:1). Yield: 167 mg (0.62 mmol, 62%). ^1^H NMR (400 MHz, DMSO-*d*_6_) δ 10.36 (s, 1H), 7.83–7.80 (m, 1H), 7.60–7.56 (m, 1H), 7.36 (d, *J* = 8.4 Hz, 2H), 7.27 (d, *J* = 8.6 Hz, 2H), 7.22–7.19 (m, 2H), 4.33 (d, *J* = 11.7 Hz, 1H), 3.62 (dd, *J* = 18.1, 12.5 Hz, 1H), 2.94 (d, *J* = 17.8 Hz, 1H). ^13^C NMR (101 MHz, DMSO-*d*_6_) δ 197.8, 173.1, 138.8, 137.6, 134.4, 131.6, 130.8 (2C), 130.1, 128.0 (2C), 126.8, 123.7, 121.9, 45.8, 42.3. IR, v_max_/cm^−1^: 3297, 2994, 2370, 1771, 1383, 1242, 1057. HRMS (ES TOF) calculated for [M + Na]^+^ C_16_H_12_ClNNaO_2_ 308.0449, found 308.0440 (−2.9 ppm).

**3-(4-Bromophenyl)-3,4-dihydro-1*H*-benzo[b]azepine-2,5-dione (16e)**. Colorless solid, mp (EtOAc) 191.8–193.4 °C, R*_f_* 0.46 (EtOAc/Hex, 1:1). Yield: 214 mg (0.65 mmol, 65%). ^1^H NMR (400 MHz, DMSO-*d*_6_) δ 10.36 (s, 1H), 7.83–7.80 (m, 1H), 7.60–7.56 (m, 1H), 7.51–7.48 (m, 2H), 7.22–7.19 (m, 4H), 4.32 (dd, *J* = 12.5, 1.9 Hz, 1H), 3.61 (dd, *J* = 18.0, 12.5 Hz, 1H), 2.93 (dd, *J* = 18.1, 2.0 Hz, 1H). ^13^C NMR (101 MHz, DMSO-*d*_6_) δ 197.8, 173.1, 138.8, 138.0, 134.4, 131.2 (2C), 130.9 (2C), 130.1, 126.8, 123.8, 121.9, 120.1, 45.8, 42.4. IR, v_max_/cm^−1^: 2927, 2386, 1773, 1664, 1244, 1043, 769. HRMS (ES TOF) calculated for [M + Na]^+^ C_16_H_12_BrNNaO_2_ 351.9944, found 351.9951 (2.0 ppm).

**3-(*p*-Tolyl)-3,4-dihydro-1*H*-benzo[b]azepine-2,5-dione (16f)**. Colorless solid, mp (EtOAc) 158.3–160.37 °C, R*_f_* 0.34 (EtOAc/Hex, 1:2). Yield: 193 mg (0.73 mmol, 73%). ^1^H NMR (400 MHz, CDCl_3_) δ 8.69 (s, 1H), 7.97 (d, *J* = 7.9 Hz, 1H), 7.48 (t, *J* = 7.6 Hz, 1H), 7.20 (t, *J* = 7.6 Hz, 1H), 7.14 (d, *J* = 8.2 Hz, 2H), 7.08 (d, *J* = 8.2 Hz, 2H), 6.97 (d, *J* = 8.2 Hz, 1H), 3.50 (dd, *J* = 18.1, 11.8 Hz, 1H), 3.21 (dd, *J* = 18.1, 2.0 Hz, 1H), 2.32 (s, 3H). ^13^C NMR (101 MHz, CDCl_3_) δ 197.8, 174.9, 137.8, 137.4, 134.7, 133.6, 131.0, 129.5 (2C), 128.3 (2C), 127.4, 124.8, 121.8, 46.4, 43.8, 21.2. IR, v_max_/cm^−1^: 3233, 2994, 1737, 1688, 1562, 1509, 1473, 1385, 1250, 1169. HRMS (ES TOF) calculated for [M + Na]^+^ C_17_H_17_NNaO_4_ 288.0995, found 288.0985 (3.5 ppm).

**3-(3-Methoxyphenyl)-3,4-dihydro-1*H*-benzo[b]azepine-2,5-dione (16g)**. Colorless solid, mp (EtOAc) 169.7–170.2 °C, R*_f_* 0.47 (EtOAc/Hex, 1:1). Yield: 163 mg (0.58 mmol, 58%). ^1^H NMR (400 MHz, CDCl_3_) δ 8.76 (s, 1H), 7.97 (d, *J* = 7.9 Hz, 1H), 7.48 (t, *J* = 7.6 Hz, 1H), 7.20 (t, *J* = 7.5 Hz, 1H), 7.11 (d, *J* = 8.2 Hz, 2H), 6.97 (d, *J* = 8.1 Hz, 1H), 6.86 (d, *J* = 8.2 Hz, 2H), 4.19–4.11 (m, 1H), 3.78 (s, 3H), 3.48 (dd, *J* = 18.1, 11.7 Hz, 1H), 3.24–3.14 (m, 1H). ^13^C NMR (101 MHz, CDCl_3_) δ 197.8, 175.1, 159.0, 137.8, 134.7, 131.0, 129.5 (2C), 128.7, 127.5, 124.9, 121.8, 114.2 (2C), 55.3, 46.6, 43.4. IR, v_max_/cm^−1^: 3209, 2990, 1733, 1686, 1558, 1505, 1475, 1433, 1244. HRMS (ES TOF) calculated for [M + Na]^+^ C_17_H_15_NNaO_3_ 304.0944, found 304.0939 (1.8 ppm).

**3-(4-Isopropylphenyl)-3,4-dihydro-1*H*-benzo[b]azepine-2,5-dione (16h)**. Colorless solid, mp (EtOAc) 139.2–140.1 °C, R*_f_* 0.44 (EtOAc/Hex, 1:2). Yield: 217 mg (0.74 mmol, 74%). ^1^H NMR (400 MHz, DMSO-*d*_6_) δ 10.32 (s, 1H), 7.81 (dd, *J* = 7.9, 1.7 Hz, 1H), 7.57 (ddd, *J* = 8.2, 7.2, 1.7 Hz, 1H), 7.24–7.17 (m, 2H), 7.16–7.09 (m, 4H), 4.22 (dd, *J* = 12.0, 1.9 Hz, 1H), 3.56 (dd, *J* = 17.9, 12.1 Hz, 1H), 2.98 (dd, *J* = 18.0, 1.9 Hz, 1H), 2.85 (hept, *J* = 6.9 Hz, 1H), 1.18 (d, *J* = 7.3 Hz, 6H). ^13^C NMR (101 MHz, DMSO-*d*_6_) δ 197.9, 173.6, 146.80, 139.0, 135.7, 134.3, 130.0, 128.6 (2C), 126.7, 126.0 (2C), 123.6, 121.8, 45.9, 42.8, 33.1, 24.0, 23.9. IR, v_max_/cm^−1^: 3209, 2990, 1733, 1686, 1558, 1519, 1505, 1457, 1433, 1393, 1244. HRMS (ES TOF) calculated for [M + Na]^+^ C_19_H_19_NNaO_2_ 316.1308, found 316.1316 (2.5 ppm).

**3-(3,4-Dimethylphenyl)-3,4-dihydro-1*H*-benzo[b]azepine-2,5-dione (16i)**. Colorless solid, mp (EtOAc) 194.6–195.9 °C, R*_f_* 0.28 (EtOAc/Hex, 1:1). Yield: 200 mg (0.72 mmol, 72%). ^1^H NMR (400 MHz, CDCl_3_) δ 7.99 (dd, *J* = 8.0, 1.7 Hz, 1H), 7.90 (s, 1H), 7.55–7.50 (m, 1H), 7.25–7.20 (m, 1H), 6.96 (d, *J* = 8.1 Hz, 1H), 6.82 (d, *J* = 8.2 Hz, 1H), 6.76–6.70 (m, 2H), 4.17 (dd, *J* = 11.8, 2.0 Hz, 1H), 3.85 (d, *J* = 6.5 Hz, 6 H), 3.50 (dd, *J* = 18.1, 11.8 Hz, 1H), 3.23 (dd, *J* = 18.1, 2.0 Hz, 1H). ^13^C NMR (101 MHz, CDCl_3_) δ 197.7, 174.4, 149.0, 148.6, 137.6, 134.8, 131.2, 129.0, 127.6, 125.1, 121.6, 120.6, 111.6, 111.3, 56.0 (2C), 46.6, 43.7. IR, v_max_/cm^−1^: 3193, 2939, 1775, 1737, 1668, 1521, 1504, 1473, 1455, 1242, 1151. HRMS (ES TOF) calculated for [M + Na]^+^ C_18_H_17_NNaO_2_ 302.1151, found 302.1143 (−2.6 ppm).

**3-(3-Chlorophenyl)-3,4-dihydro-1*H*-benzo[b]azepine-2,5-dione (16j)**. Colorless solid, mp (EtOAc) 221.7–222.7 °C, R*_f_* 0.4 (EtOAc/Hex, 1:2). Yield: 160 mg (0.56 mmol, 56%). ^1^H NMR (400 MHz, CDCl_3_) δ 8.00 (d, *J* = 7.9 Hz, 1H), 7.89 (s, 1H), 7.59–7.50 (m, 1H), 7.33–7.25 (m, 3H), 7.21 (s, 1H), 7.10 (ddd, *J* = 5.7, 3.2, 1.9 Hz, 1H), 6.97 (d, *J* = 8.1 Hz, 1H), 4.17 (dd, *J* = 12.3, 2.0 Hz, 1H), 3.49 (dd, *J* = 18.2, 12.3 Hz, 1H), 3.18 (dd, *J* = 18.2, 2.0 Hz, 1H). ^13^C NMR (101 MHz, CDCl_3_) δ 197.3, 173.5, 138.7, 137.3, 134.9, 131.3, 130.1, 128.9, 128.1, 127.6, 126.8, 125.4, 121.8, 46.4, 43.7, 29.9. IR, v_max_/cm^−1^: 3293, 2990, 1773, 1684, 1560, 1477, 1437, 1244. HRMS (ES TOF) calculated for [M + Na]^+^ C_16_H_12_ClNNaO_2_ 308.0449, found 308.0456 (2.3 ppm).

**3-(4-Ethylphenyl)-3,4-dihydro-1*H*-benzo[b]azepine-2,5-dione (16k)**. Colorless solid, mp (EtOAc) 159.8–162.1 °C, R*_f_* 0.28 (EtOAc/Hex, 1:1). Yield: 176 mg (0.63 mmol, 63%). ^1^H NMR (400 MHz, DMSO-*d*_6_) δ 10.32 (s, 1H), 7.80 (dd, *J* = 7.9, 1.7 Hz, 1H), 7.56 (ddd, *J* = 8.1, 7.2, 1.7 Hz, 1H), 7.22–7.16 (m, 2H), 7.12 (s, 4H), 4.21 (dd, *J* = 11.9, 1.9 Hz, 1H), 3.56 (dd, *J* = 17.9, 11.9 Hz, 1H), 2.99 (dd, *J* = 17.9, 1.9 Hz, 1H), 2.56 (d, *J* = 7.6 Hz, 2H), 1.16 (t, *J* = 7.6 Hz, 3H). ^13^C NMR (101 MHz, DMSO-*d*_6_) δ 197.8, 173.6, 142.2, 139.0, 135.4, 134.4, 130.0, 128.6 (2C), 127.5 (2C), 126.6, 123.6, 121.8, 45.8, 42.9, 27.8, 15.6. IR, v_max_/cm^−1^: 3225, 2990, 1769, 1700, 1654, 1612, 1522, 1511, 1417, 1240. HRMS (ES TOF) calculated for [M + Na]^+^ C_18_H_17_NNaO_2_ 302.1151, found 302.1160 (3.0 ppm).

### 3.5. General Procedure for Preparation of 7-Arylpaullones (***7aa–ak,ba,ca***)

A 5 mL round-bottom flask equipped with magnetic stirring bar was charged with the corresponding keto-lactam **16a–k** (0.50 mmol), phenylhydrazine (0.50 mmol), ethanol (0.25 mL), and AcOH (30 mg, 0.029 mL, 0.50 mmol). The reaction mixture was left stirring at room temperature for 30 min. After this, 0.5 g of 80% PPA was added and stirring continued at 70 °C for another 30 min. The reaction mixture was then poured into water (20 mL) and carefully neutralized with concentrated aqueous ammonia solution. The formed precipitate was filtered off, open-air dried, and purified by column chromatography using mixture EtOAc/Hex, followed by recrystallization from EtOAc.

**7-Phenyl-7,12-dihydrobenzo [2,3]azepino[4,5-b]indol-6(5*H*)-one (7aa)**. Colorless solid, mp (EtOAc) 224.9–226.1 °C, R*_f_* 0.67 (EtOAc/Hex, 1:1). Yield: 128 mg (0.40 mmol, 79%). ^1^H NMR (400 MHz, DMSO-*d*_6_) δ 11.74 (s, 1H), 10.34 (s, 1H), 7.77–7.69 (m, 2H), 7.50 (d, *J* = 8.1 Hz, 1H), 7.25–7.17 (m, 3H), 7.15 (d, *J* = 7.4 Hz, 1H), 7.11–7.02 (m, 4H), 6.91 (d, *J* = 7.4 Hz, 2H), 5.52 (s, 1H). ^13^C NMR (101 MHz, DMSO-*d*_6_) δ 171.6, 137.5, 137.4, 134.7, 132.6, 128.2 (2C), 128.1, 128.0, 127.6, 126.6, 126.4 (2C), 123.4, 122.6, 122.5, 121.3, 119.5, 117.8, 111.6, 110.1, 48.8. IR, v_max_/cm^−1^: 3229, 2994, 1757, 1652, 1558, 1487, 1459, 1248, 1063. HRMS (ES TOF) calculated for [M + Na]^+^ C_22_H_16_N_2_NaO 347.1155, found 347.1149 (−1.7 ppm).

**7-(4-Methoxyphenyl)-7,12-dihydrobenzo[2,3]azepino[4,5-b]indol-6(5*H*)-one (7ab)**. Colorless solid, mp (EtOAc) 284.2–287.2 °C, R*_f_* 0.38 (EtOAc/Hex, 1:1). Yield: 145 mg (0.41 mmol, 82%) ^1^H NMR (400 MHz, DMSO-*d*_6_) δ 11.73 (s, 1H), 10.31 (s, 1H), 7.72 (t, *J* = 6.6 Hz, 2H), 7.49 (d, *J* = 8.2 Hz, 1H), 7.23–7.13 (m, 3H), 7.08 (t, *J* = 7.5 Hz, 2H), 6.82 (d, *J* = 8.8 Hz, 2H), 6.64 (d, *J* = 8.8 Hz, 2H), 5.45 (s, 1H), 3.58 (s, 3H). ^13^C NMR (101 MHz, DMSO-*d*_6_) δ 171.8, 157.9, 137.5, 134.8, 132.5, 129.2, 128.0, 127.53, 127.50 (2C), 126.4, 123.4, 122.6, 122.5, 121.3, 119.4, 117.8, 113.6 (2C), 111.5, 110.4, 54.9, 48.1. IR, v_max_/cm^−1^: 3297, 2978, 2366, 1773, 1638, 1240, 1039. HRMS (ES TOF) calculated for [M + Na]^+^ C_23_H_18_N_2_NaO_2_ 377.1260, found 377.1251 (−2.4 ppm).

**7-(4-Fluorophenyl)-7,12-dihydrobenzo[2,3]azepino[4,5-b]indol-6(5*H*)-one (7ac)**. Colorless solid, mp (EtOAc) 298.8–303.1 °C, R*_f_* 0.36 (EtOAc/Hex, 1:1). Yield: 147 mg (0.43 mmol, 86%). ^1^H NMR (400 MHz, DMSO-*d*_6_) δ 11.77 (s, 1H), 10.37 (s, 1H), 7.76–7.71 (m, 2H), 7.50 (d, *J* = 8.2 Hz, 1H), 7.24–7.19 (m, 2H), 7.17–7.13 (m, 1H), 7.11–7.05 (m, 2H), 6.92 (d, *J* = 7.2 Hz, 4H), 5.51 (s, 1H). ^13^C NMR (101 MHz, DMSO-*d*_6_) δ 171.4, 160.8 (d, *J* = 242.8 Hz), 137.5, 134.6, 133.5 (d, *J* = 2.9 Hz), 132.6, 128.3 (d, *J* = 8.4 Hz, 2C), 128.1, 127.4, 126.4, 123.5, 122.6, 122.5, 121.4, 119.5, 117.8, 115.0 (d, *J* = 21.3 Hz, 2C), 111.6, 110.1, 48.0. ^19^F NMR (376 MHz, DMSO-*d*_6_) δ -116.3. IR, v_max_/cm^−1^: 3297, 2919, 1726, 1640, 1499, 1220, 1029. HRMS (ES TOF) calculated for [M + Na]^+^ C_22_H_15_FN_2_NaO 365.1061, found 365.1072 (3.0 ppm).

**7-(4-Chlorophenyl)-7,12-dihydrobenzo[2,3]azepino[4,5-b]indol-6(5*H*)-one (7ad)**. Colorless solid, mp (EtOAc) 291.0–293.0 °C, R*_f_* 0.33 (EtOAc/Hex, 1:1). Yield: 143 mg (0.40 mmol, 80%). ^1^H NMR (400 MHz, DMSO-*d*_6_) δ 11.79 (s, 1H), 10.40 (s, 1H), 7.76 (d, *J* = 7.9 Hz, 1H), 7.72–7.70 (m, 1H), 7.50 (d, *J* = 8.2 Hz, 1H), 7.25–7.19 (m, 2H), 7.17–7.13 (m, 3H), 7.11–7.05 (m, 2H), 6.90–6.88 (m, 2H), 5.52 (s, 1H). ^13^C NMR (101 MHz, DMSO-*d*_6_) δ 171.2, 137.5, 136.4, 134.5, 132.6, 131.2, 128.24 (2C), 128.23 (2C), 128.1, 127.5, 126.4, 123.6, 122.7, 122.4, 121.4, 119.6, 117.9, 111.6, 109.8, 48.1. IR, v_max_/cm^−1^: 3281, 3010, 2362, 1771, 1238, 1051. HRMS (ES TOF) calculated for [M + Na]^+^ C_22_H_15_ClN_2_NaO 381.0765, found 381.0770 (1.3 ppm).

**7-(4-Bromophenyl)-7,12-dihydrobenzo[2,3]azepino[4,5-b]indol-6(5*H*)-one (7ae)**. Colorless solid, mp (EtOAc) 273.2–274.5 °C, R*_f_* 0.47 (EtOAc/Hex, 1:1). Yield: 155 mg (0.39 mmol, 77%). ^1^H NMR (400 MHz, DMSO-*d*_6_) δ 11.81 (s, 1H), 10.40 (s, 1H), 7.76 (d, *J* = 7.9 Hz, 1H), 7.72 (d, *J* = 6.5 Hz, 1H), 7.50 (d, *J* = 7.7 Hz, 1H), 7.29 (d, *J* = 8.6 Hz, 2H), 7.25–7.19 (m, 2H), 7.17–7.13 (m, 1H), 7.11–7.06 (m, 2H), 6.83 (d, *J* = 8.6 Hz, 2H), 5.50 (s, 1H). ^13^C NMR (101 MHz, DMSO-*d*_6_) δ 171.1, 137.5, 136.9, 134.5, 132.6, 131.1 (2C), 128.6 (2C), 128.2, 127.5, 126.5, 123.6, 122.7, 122.4, 121.4, 119.8, 119.6, 117.9, 111.6, 109.7, 48.1. IR, v_max_/cm^−1^: 2994, 2362, 1773, 1248, 1053. HRMS (ES TOF) calculated for [M + Na]^+^ C_22_H_15_BrN_2_NaO 425.0260, found 425.0272 (2.8 ppm).

**7-(p-Tolyl)-7,12-dihydrobenzo[2,3]azepino[4,5-b]indol-6(5*H*)-one (7af)**. Yellowish solid, mp (EtOAc) 252.3–255.1 °C, R*_f_* 0.55 (EtOAc/Hex, 1:1). Yield: 125 mg (0.36 mmol, 71%). ^1^H NMR (400 MHz, DMSO-*d*_6_) δ 11.72 (s, 1H), 10.31 (s, 1H), 7.75–7.67 (m, 2H), 7.49 (d, *J* = 8.2 Hz, 1H), 7.24–7.17 (m, 2H), 7.16–7.11 (m, 1H), 7.10–7.03 (m, 2H), 6.88 (d, *J* = 7.9 Hz, 2H), 6.78 (d, *J* = 8.3 Hz, 2H), 5.46 (s, 1H), 2.10 (s, 3H). ^13^C NMR (101 MHz, DMSO-*d*_6_) δ 171.7, 137.5, 135.7, 134.7, 134.4, 132.5, 128.8 (2C), 128.0, 127.6, 126.4, 126.3 (2C), 123.4, 122.6, 122.5, 121.3, 119.4, 117.8, 111.5, 110.2, 48.4, 20.5. IR, v_max_/cm^−1^: 3225, 2991, 1749, 1655, 1555, 1482, 1452, 1248. HRMS (ES TOF) calculated for [M + Na]^+^ C_23_H_18_N_2_NaO 361.1311, found 361.1305 (−1.7 ppm).

**7-(3-Methoxyphenyl-7,12-dihydrobenzo [2,3]azepino [4,5-b]indol-6(5*H*)-one (7ag)**. Colorless solid, mp (EtOAc) 268.6–270.9 °C, R*_f_* 0.45 (EtOAc/Hex, 1:1). Yield: 135 mg (0.38 mmol, 76%). ^1^H NMR (400 MHz, DMSO-*d*_6_) δ 11.74 (s, 1H), 10.30 (s, 1H), 7.72 (t, *J* = 6.9 Hz, 2H), 7.49 (d, *J* = 8.1 Hz, 1H), 7.18 (dt, *J* = 24.3, 7.2 Hz, 3H), 7.07 (d, *J* = 7.8 Hz, 2H), 6.81 (d, *J* = 8.6 Hz, 2H), 6.64 (d, *J* = 8.6 Hz, 2H), 5.44 (s, 1H), 3.58 (s, 3H). ^13^C NMR (101 MHz, DMSO-*d*_6_) δ 171.8, 157.9, 137.5, 134.7, 132.5, 129.2, 128.0, 127.52, 127.50 (2C), 126.4, 123.4, 122.6, 122.5, 121.3, 119.4, 117.8, 113.6 (2C), 111.6, 110.4, 54.9, 48.1. IR, v_max_/cm^−1^: 3015, 2895, 1773, 1759, 1654, 1556, 1506, 1451, 1363, 1159. HRMS (ES TOF) calculated for [M + Na]^+^ C_23_H_18_N_2_NaO_2_ 377.1260, found 377.1256 (−1.1 ppm).

**7-(4-Isopropylphenyl)-7,12-dihydrobenzo [2,3]azepino [4,5-b]indol-6(5*H*)-one (7ah)**. Colorless solid, mp (EtOAc) 234.6–235.9 °C, R*_f_* 0.51 (EtOAc/Hex, 1:1). Yield: 148 mg (0.40 mmol, 81%). ^1^H NMR (400 MHz, DMSO-*d*_6_) δ 11.71 (s, 1H), 10.33 (d, *J* = 1.7 Hz, 1H), 7.78–7.68 (m, 2H), 7.48 (d, *J* = 8.1 Hz, 1H), 7.24–7.18 (m, 2H), 7.18–7.13 (m, 1H), 7.11–7.06 (m, 2H), 6.96 (d, *J* = 8.3 Hz, 2H), 6.84 (d, *J* = 8.1 Hz, 2H), 5.47 (s, 1H), 2.69 (p, *J* = 6.9 Hz, 1H), 1.04 (dd, *J* = 6.9, 3.3 Hz, 6H). ^13^C NMR (101 MHz, DMSO-*d*_6_) δ 171.7, 146.7, 137.5, 134.9, 134.7, 132.4, 128.1, 127.5, 126.4 (3 C), 126.2 (2C), 123.4, 122.6, 122.3, 121.3, 119.4, 117.8, 111.5, 110.1, 48.63, 32.8, 23.8, 23.7. IR, v_max_/cm^−1^: 2986, 2891, 1777, 1700, 1638, 1521, 1457, 1419, 1244, 1049. HRMS (ES TOF) calculated for [M + Na]^+^ C_25_H_22_N_2_NaO 389.1624, found 389.1630 (1.5 ppm).

**7-(3,4-Dimethylphenyl)-7,12-dihydrobenzo[2,3]azepino[4,5-b]indol-6(5*H*)-one (7ai)**. Colorless solid, mp (EtOAc) 192.8–195.4 °C, R*_f_* 0.55 (EtOAc). Yield: 134 mg (0.38 mmol, 76%). ^1^H NMR (400 MHz, DMSO-*d*_6_) δ 11.73 (s, 1H), 10.26 (s, 1H), 7.74 (dd, *J* = 18.3, 7.8 Hz, 2H), 7.52–7.45 (m, 1H), 7.28–7.15 (m, 3H), 7.08 (t, *J* = 7.1 Hz, 2H), 6.69–6.63 (m, 1H), 6.51–6.45 (m, 2H), 5.45 (s, 1H), 3.58 (d, *J* = 2.3 Hz, 3H), 3.38 (d, *J* = 2.4 Hz, 3H). ^13^C NMR (101 MHz, DMSO-*d*_6_) δ 171.8, 148.1, 147.5, 137.5, 134.9, 132.5, 129.6, 128.1, 127.4, 126.4, 123.5, 122.7, 122.6, 121.3, 119.4, 119.0, 117.8, 111.6, 111.5, 110.3, 110.2, 55.3, 55.0, 48.6. IR, v_max_/cm^−1^: 2990, 1773, 1759, 1634, 1560, 1507, 1457, 1378, 1246, 1135, 1055. HRMS (ES TOF) calculated for [M + Na]^+^ C_24_H_20_N_2_NaO 375.1468, found 375.1475 (1.8 ppm).

**7-(3-Chlorophenyl)-7,12-dihydrobenzo[2,3]azepino[4,5-b]indol-6(5*H*)-one (7aj)**. Colorless solid, mp (EtOAc) 243.9–245.9 °C, R*_f_* 0.5 (EtOAc/Hex, 1:1). Yield: 120 mg (0.34 mmol, 67%). ^1^H NMR (400 MHz, DMSO-*d*_6_) δ 11.81 (s, 1H), 10.41 (s, 1H), 7.78 (d, *J* = 7.9 Hz, 1H), 7.73 (dd, *J* = 7.7, 1.7 Hz, 1H), 7.51 (d, *J* = 8.0 Hz, 1H), 7.25–7.20 (m, 3H), 7.18 (dd, *J* = 7.5, 1.4 Hz, 1H), 7.15–7.11 (m, 2H), 7.11–7.04 (m, 1H), 6.90–6.86 (m, 1H), 6.85 (d, *J* = 1.7 Hz, 1H), 5.57 (s, 1H). ^13^C NMR (101 MHz, DMSO-*d*_6_) δ 171.0, 139.9, 137.5, 134.5, 132.8, 132.7, 130.2, 128.2, 127.4, 126.7, 126.5, 126.2, 125.2, 123.7, 122.7, 122.4, 121.4, 119.6, 117.9, 111.6, 109.5, 48.3. IR, v_max_/cm^−1^: 3002, 1773, 1759, 1638, 1556, 1509, 1451, 1378, 1242, 1053. HRMS (ES TOF) calculated for [M + Na]^+^ C_22_H_15_ClN_2_NaO 381.0765, found 381.0773 (2.1 ppm).

**7-(4-Ethylphenyl)-7,12-dihydrobenzo[2,3]azepino[4,5-b]indol-6(5*H*)-one (7ak)**. Colorless solid, mp (EtOAc) 241.0–243.2 °C, R*_f_* 0.49 (EtOAc). Yield: 139 mg (0.4 mmol, 79%). ^1^H NMR (400 MHz, DMSO-*d*_6_) δ 11.73 (s, 1H), 10.32 (d, *J* = 1.7 Hz, 1H), 7.76–7.69 (m, 2H), 7.49 (d, *J* = 8.2 Hz, 1H), 7.25–7.17 (m, 2H), 7.17–7.12 (m, 1H), 7.11–7.05 (m, 2H), 6.92 (d, *J* = 8.3 Hz, 2H), 6.82 (d, *J* = 8.3 Hz, 2H), 5.47 (s, 1H), 2.40 (q, *J* = 7.5 Hz, 2H), 1.02 (t, *J* = 7.6 Hz, 3H).^13^C NMR (101 MHz, DMSO-*d*_6_) δ 171.7, 142.0, 137.5, 134.7 (2C), 132.5, 128.0, 127.7 (2C), 127.6, 126.4, 126.3 (2C), 123.4, 122.5, 122.4, 121.3, 119.4, 117.8, 111.5, 110.2, 48.5, 27.6, 15.4. IR, v_max_/cm^−1^: 3283, 3007, 2365, 1773, 1652, 1555, 1481, 1229, 1055. HRMS (ES TOF) calculated for [M + Na]^+^ C_24_H_20_N_2_NaO 375.1468, found 375.1457 (−2.9 ppm).

**9-Bromo-7-phenyl-7,12-dihydrobenzo[2,3]azepino[4,5-b]indol-6(5*H*)-one (7ba)**. Yellowish solid, mp (EtOAc) 295.2–297.4 °C, R*_f_* 0.39 (EtOAc/Hex, 1:1). Yield: 165 mg (0.41 mmol, 82%). ^1^H NMR (400 MHz, DMSO-*d*_6_) δ 11.97 (s, 1H), 10.36 (s, 1H), 8.03 (s, 1H), 7.70 (d, *J* = 7.8 Hz, 1H), 7.45 (d, *J* = 8.6 Hz, 1H), 7.33 (t, *J* = 9.7 Hz, 1H), 7.22 (t, *J* = 7.6 Hz, 2H), 7.14 (t, *J* = 7.5 Hz, 1H), 7.11–6.99 (m, 3H), 6.89 (d, *J* = 7.4 Hz, 2H), 5.58 (s, 1H). ^13^C NMR (101 MHz, DMSO-*d*_6_) δ 171.5, 137.2, 136.1, 134.9, 134.1, 129.4, 128.5, 128.2 (2C), 126.7, 126.5, 126.4 (2C), 125.0, 123.5, 122.0, 121.4, 120.4, 113.5, 112.1, 109.8, 48.4. IR, v_max_/cm^−1^: 3277, 2994, 1769, 1757, 1638, 1558, 1455, 1401, 1240, 1045. HRMS (ES TOF) calculated for [M + Na]^+^ C_22_H_15_BrN_2_NaO 425.0260, found 425.0271 (2.6 ppm).

**9,10-Dimethyl-7-phenyl-7,12-dihydrobenzo[2,3]azepino[4,5-b]indol-6(5*H*)-one (7ca)**. Yellowish solid, mp (EtOAc) 252.3–255.1 °C, R*_f_* 0.55 (EtOAc/Hex, 1:1). Yield: 125 mg (0.36 mmol, 71%). ^1^H NMR (400 MHz, DMSO-*d*_6_) δ (s, 1H), 10.29 (s, 1H), 7.68 (d, *J* = 9.5 Hz, 1H), 7.48 (s, 1H), 7.26 (s, 1H), 7.17–7.11 (m, 2H), 7.10–7.02 (m, 4H), 6.89 (d, *J* = 7.3 Hz, 2H), 5.44 (s, 1H), 2.35 (s, 3H), 2.30 (s, 3H). ^13^C NMR (101 MHz, DMSO-*d*_6_) δ 171.5, 137.5, 136.5, 134.4, 131.6, 131.3, 128.2 (2C), 127.7, 127.6, 126.6, 126.4 (2C), 126.2, 126.1, 123.4, 122.7, 121.3, 117.8, 111.8, 109.7, 48.9, 20.3, 19.9. IR, v_max_/cm^−1^: 2986, 2893, 1777, 1710, 1635, 1522, 1505, 1451, 1363, 1149. HRMS (ES TOF) calculated for [M + Na]^+^ C_24_H_20_N_2_NaO 375.1468, found 375.1472 (−1.6 ppm).

## 4. Conclusions

In conclusion, a novel approach towards previously unknown paullone analogs bearing aryl substituent at C-7 was developed. Such paullones could be promising candidates for anticancer drug development as they are structurally similar to 2-(1*H*-indol-3-yl)acetamides known for their submicromolar antitumor activity. The reported synthetic approach is based on the reaction of readily available cyanoketones **14** and involves three steps: (1) acid-mediated hydrolysis of nitrile function; (2) assembly of a seven-membered lactam ring; and (3) Fischer indolization. Evaluation of the bioactivity of the synthesized paullones **7** is currently under way.

## Figures and Tables

**Table 1:** Screening of the reaction conditions for Fisher indolization of keto-lactam **16a** to 7-phenyl paullone **7aa**.

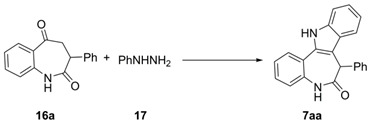					
No.	Acid 1 g/mmol	Solvent	Temp. (°C)	Time (h)	Yield (%)
1	PPA 80%	-	100	0.5	3
2	PPA 80%	-	80	1	6
3	PPA 87%	-	80	1	-
4	PPA 87% (0.5 g)/H_3_PO_3_ (0.5 g)	-	80	1	-
5	MsOH	-	90	1	-
6	PPA 80%	EtOAc	70	1	29
7	PPA 80%	EtOH	70	1	24
8.1 8.2	EtOH/AcOH (1 mmol) Then, PPA 80% (0.5 g)	EtOH EtOH	r.t. 70	0.5 0.5	- 79

## Data Availability

Supporting data including NMR spectral charts are available from the authors.

## Supplementary Materials

The following supporting information can be downloaded at: https://www.mdpi.com/article/10.3390/molecules28052324/s1, Figures S1–S4: ^1^H and ^13^C NMR spectral charts for starting cyanoketones **14g,i**; Figures S5–S30: ^1^H and ^13^C NMR spectral charts for 7-arylpaullones **7aa-ak,ba,ca**; Figures S31–S52: ^1^H and ^13^C NMR spectral charts for 4-(2-aminophenyl)-4-oxo-2-arylbutanoic acids **15a-k**; Figures S53–S74: ^1^H and ^13^C NMR spectral charts for 3-aryl-3,4-dihydro-1*H*-benzo[b]azepine-2,5-diones **16a-k**.

## Sample Availability

Not available.
