# Supplementary material for: An Effective Synthesis of Previously Unknown 7-Aryl Substituted Paullones
Source: Molecules. 2023 Mar 2;28(5):2324. doi: 10.3390/molecules28052324 (PMC10005103; doi:10.3390/molecules28052324)
Supplement: Supplementary file 1 [file molecules-28-02324-s001.zip › molecules-2256298-supplementary.pdf]

^{1}H and ^{13}C NMR spectral charts for 4-(2-aminophenyl)-2-aryl-4-oxo-butanenitriles **14g,i**

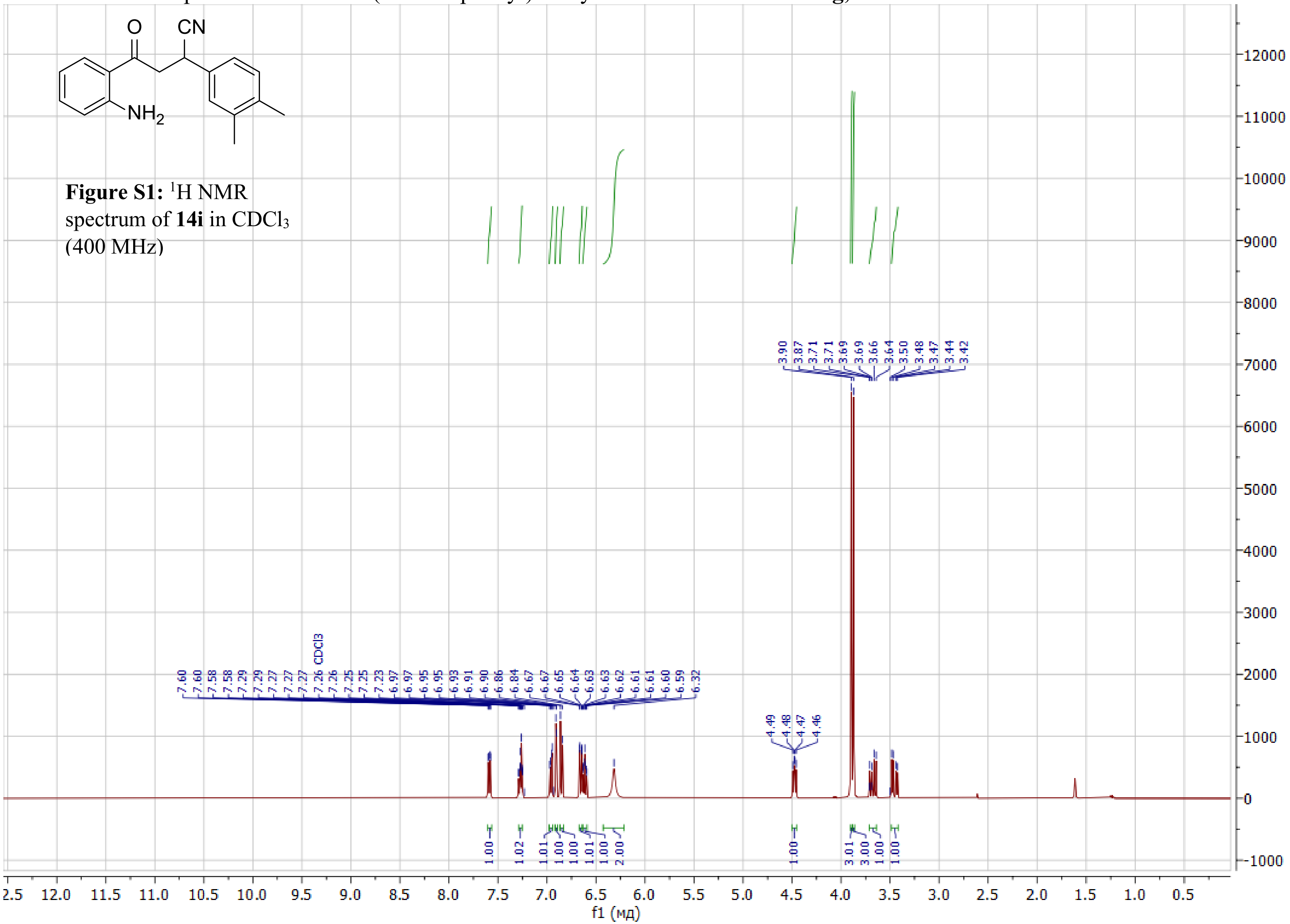

Figure S1: ^{1}H NMR spectrum of **14i** in $CDCl_3$ (400 MHz)

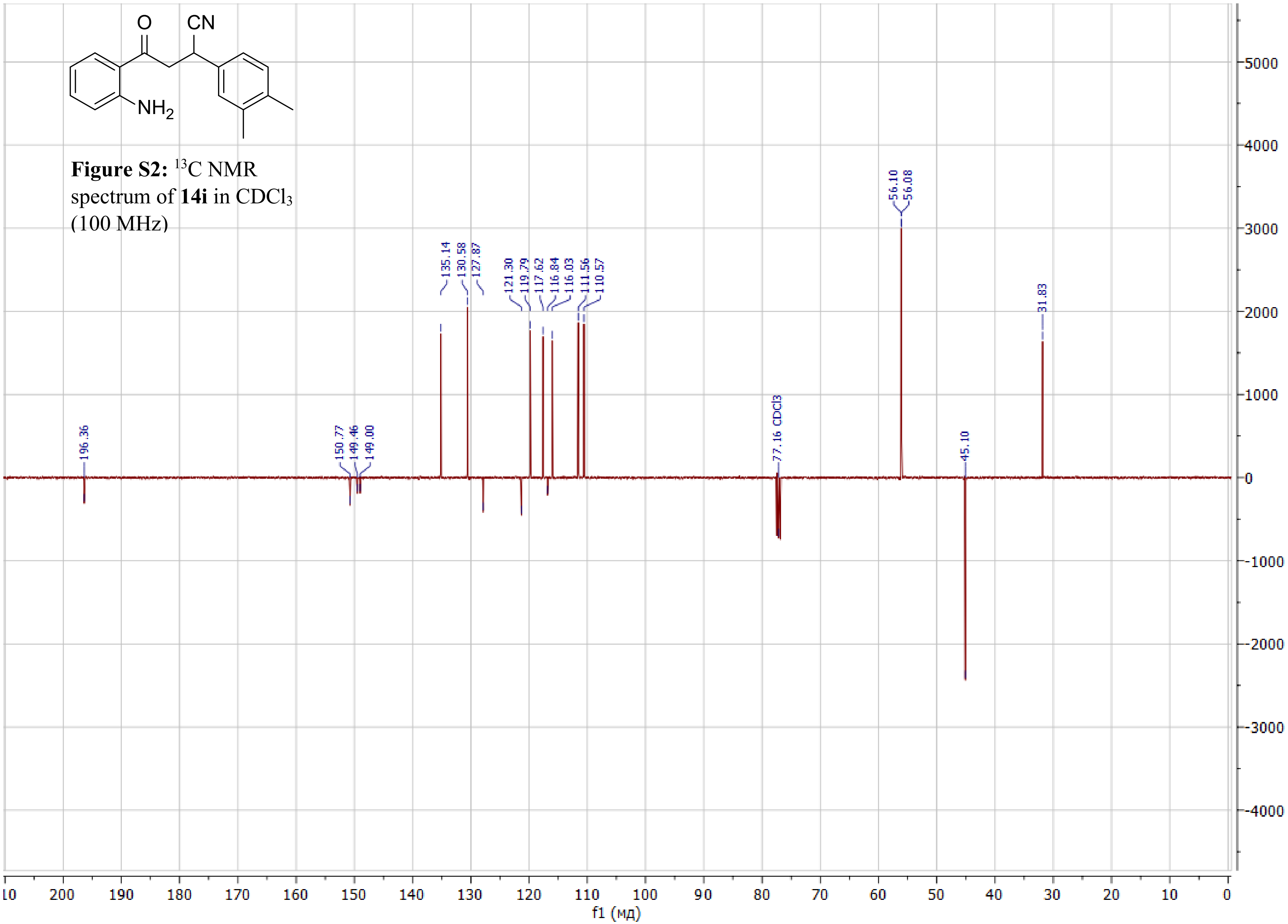

Figure S2: ^{13}C NMR spectrum of **14i** in $CDCl_3$ (100 MHz)

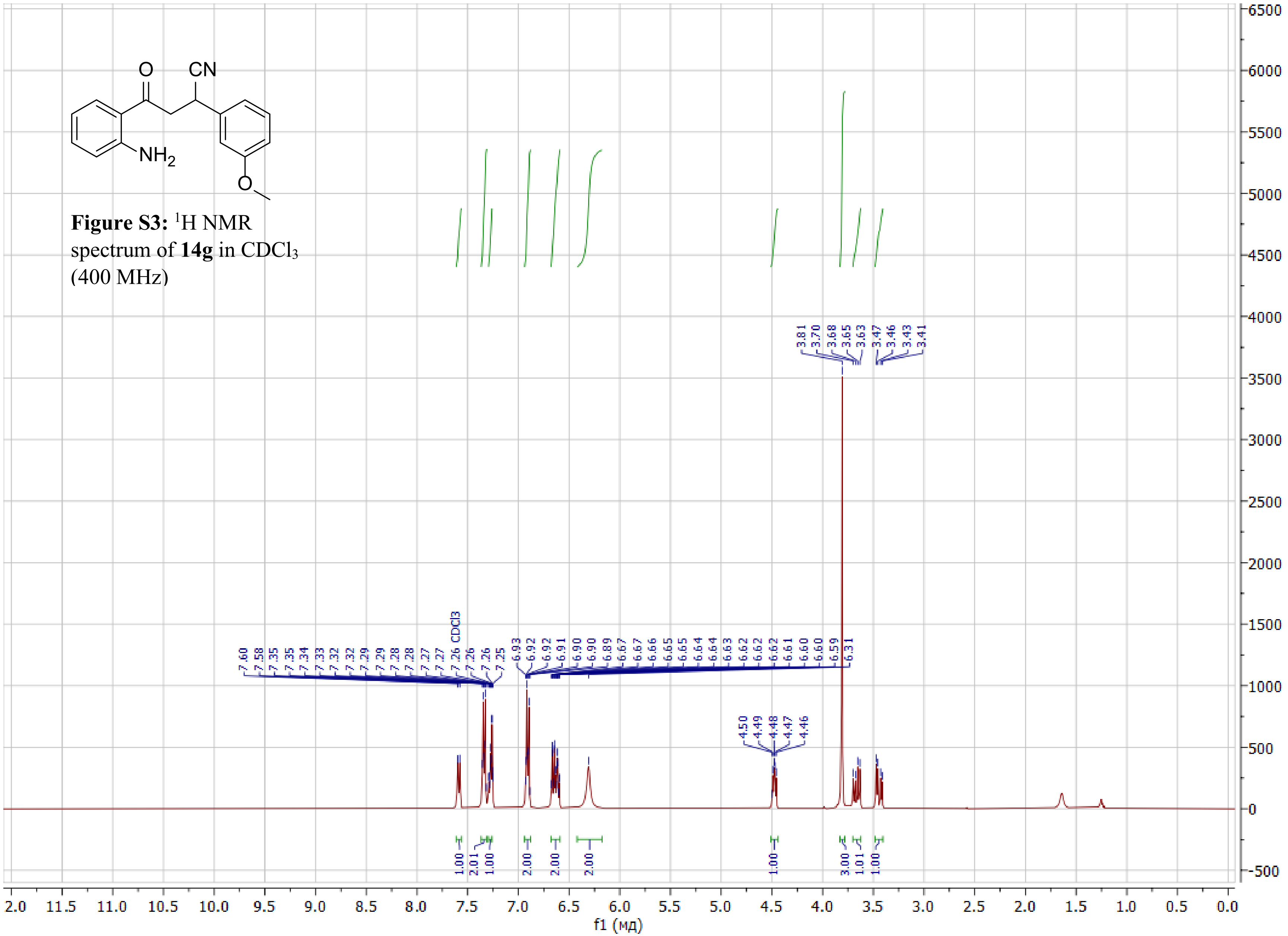

Figure S3: ^{1}H NMR spectrum of **14g** in $CDCl_3$ (400 MHz)

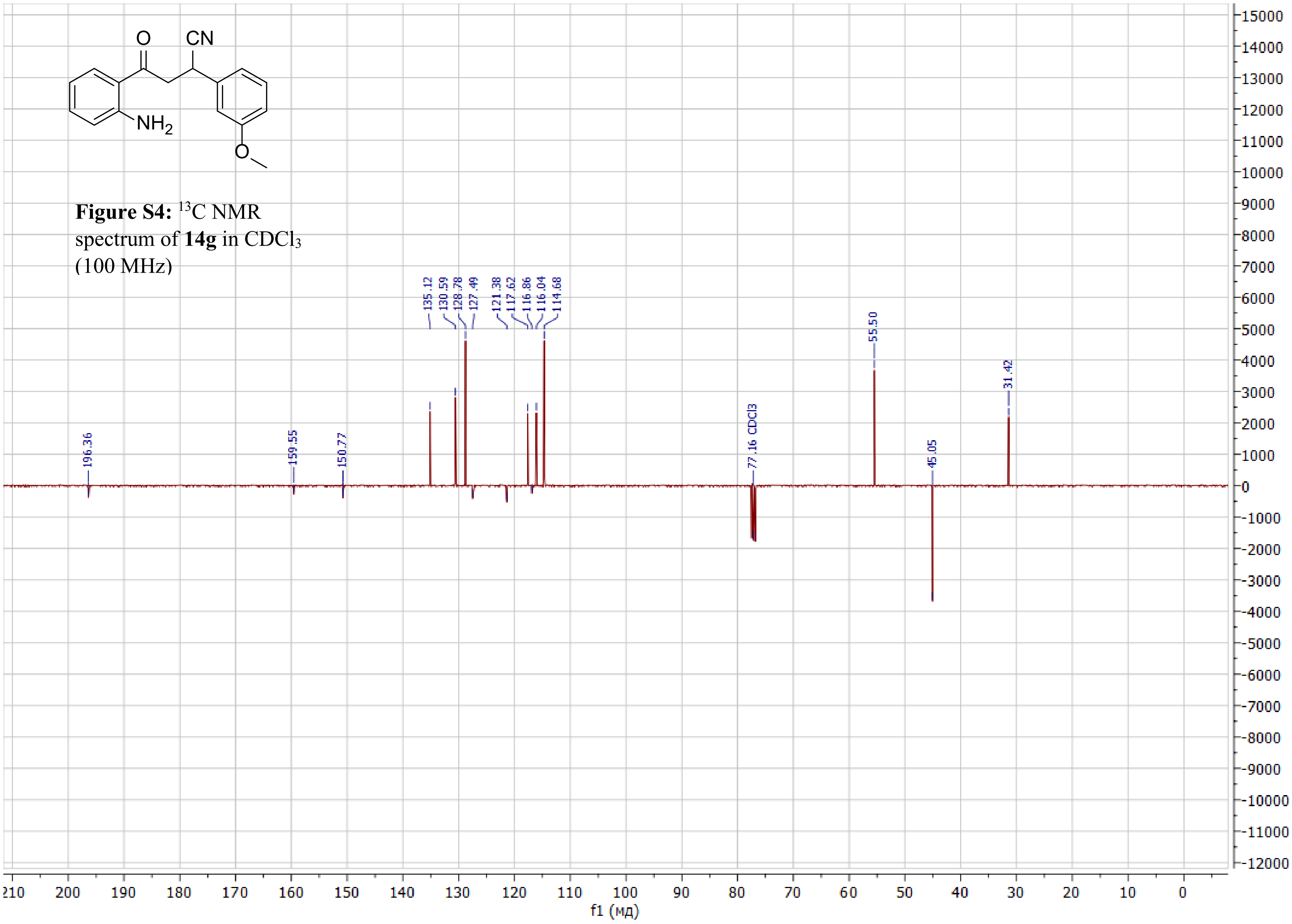

Figure S4: ^{13}C NMR spectrum of **14g** in $CDCl_3$ (100 MHz)

^{1}H and ^{13}C NMR spectral charts for 7-arylpaullones **7aa-ak,ba,ca**

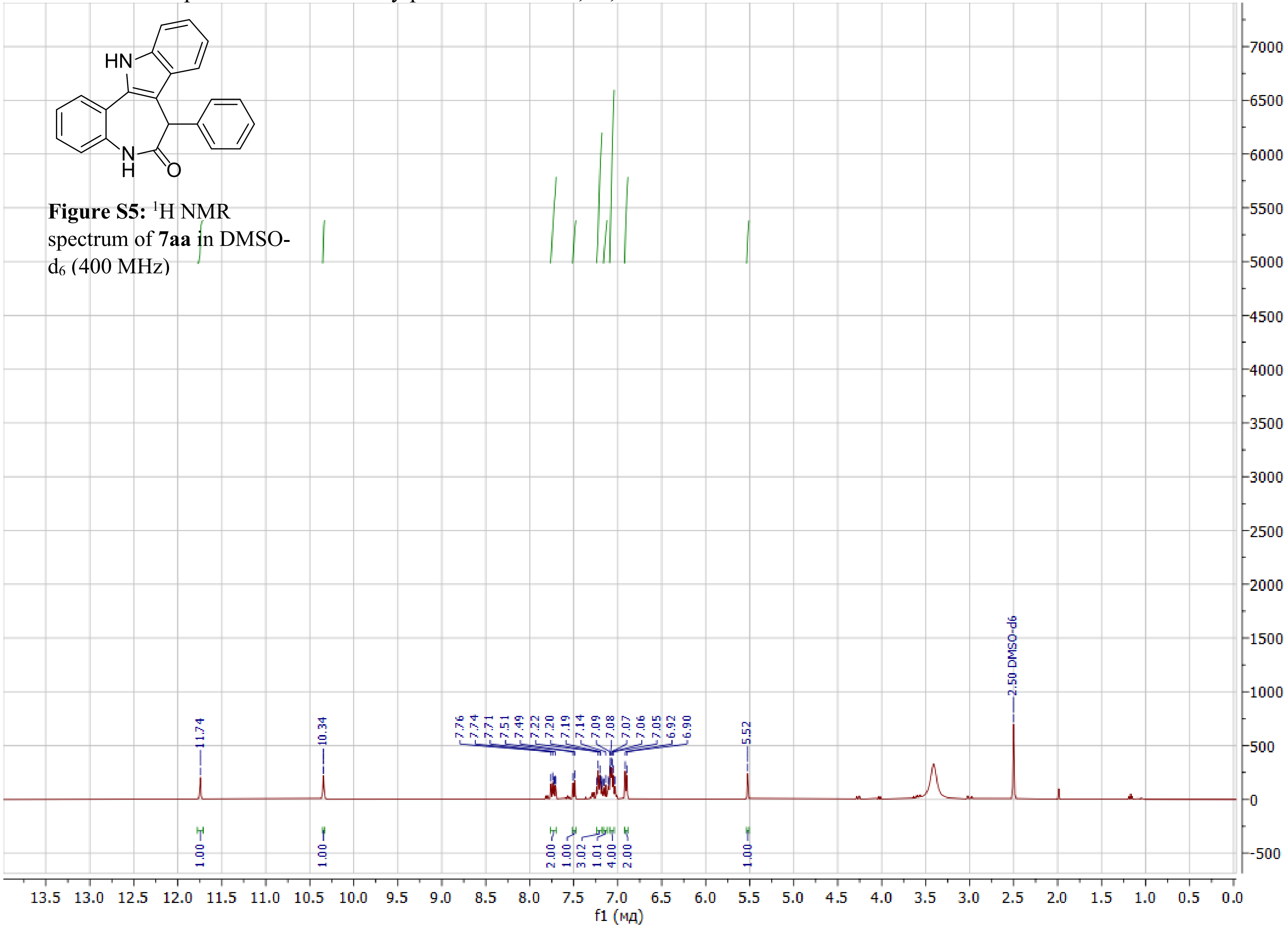

Figure S5: ^{1}H NMR spectrum of **7aa** in DMSO-d_6 (400 MHz)

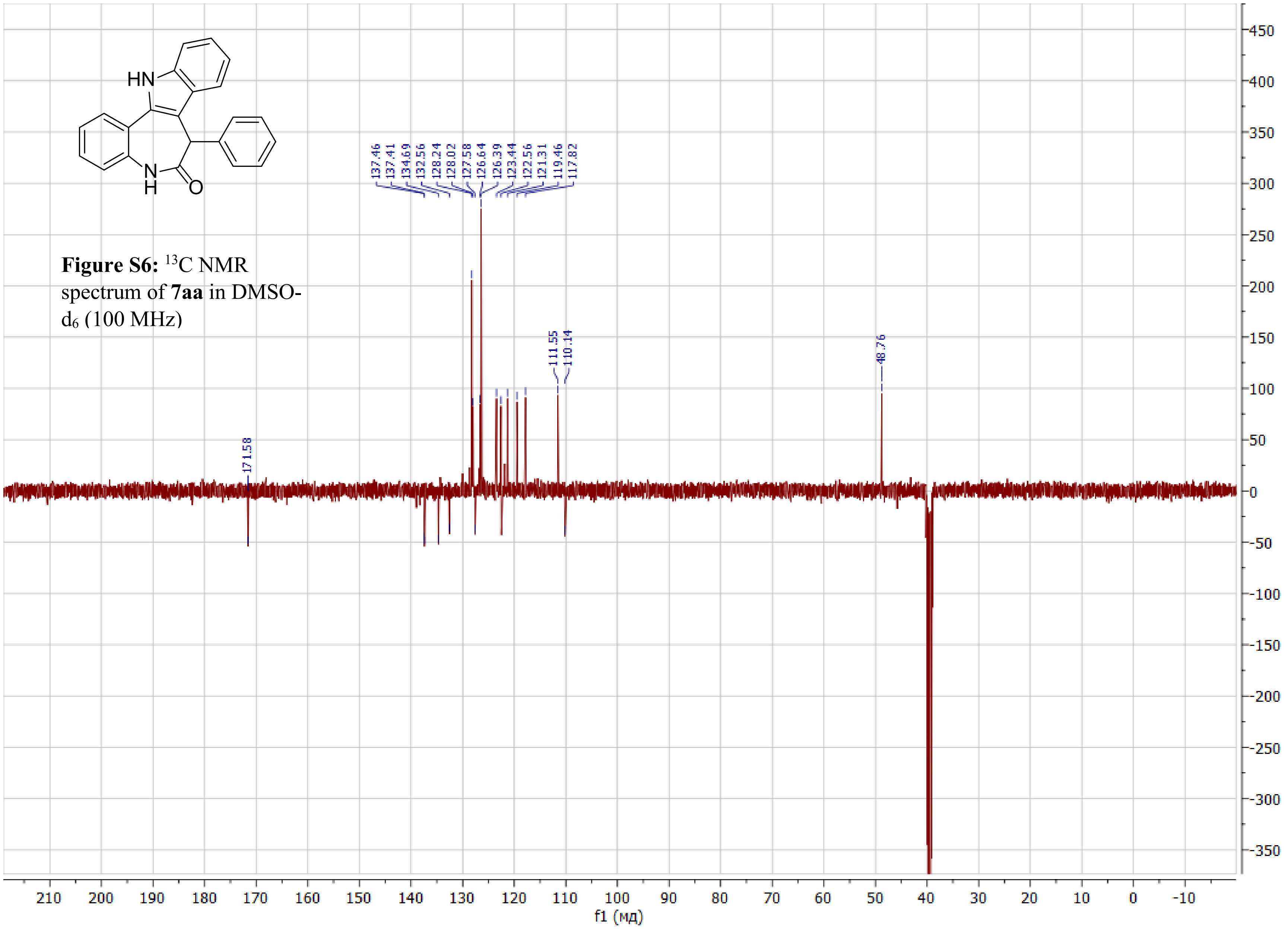

Figure S6: ^{13}C NMR spectrum of **7aa** in DMSO-d_6 (100 MHz)

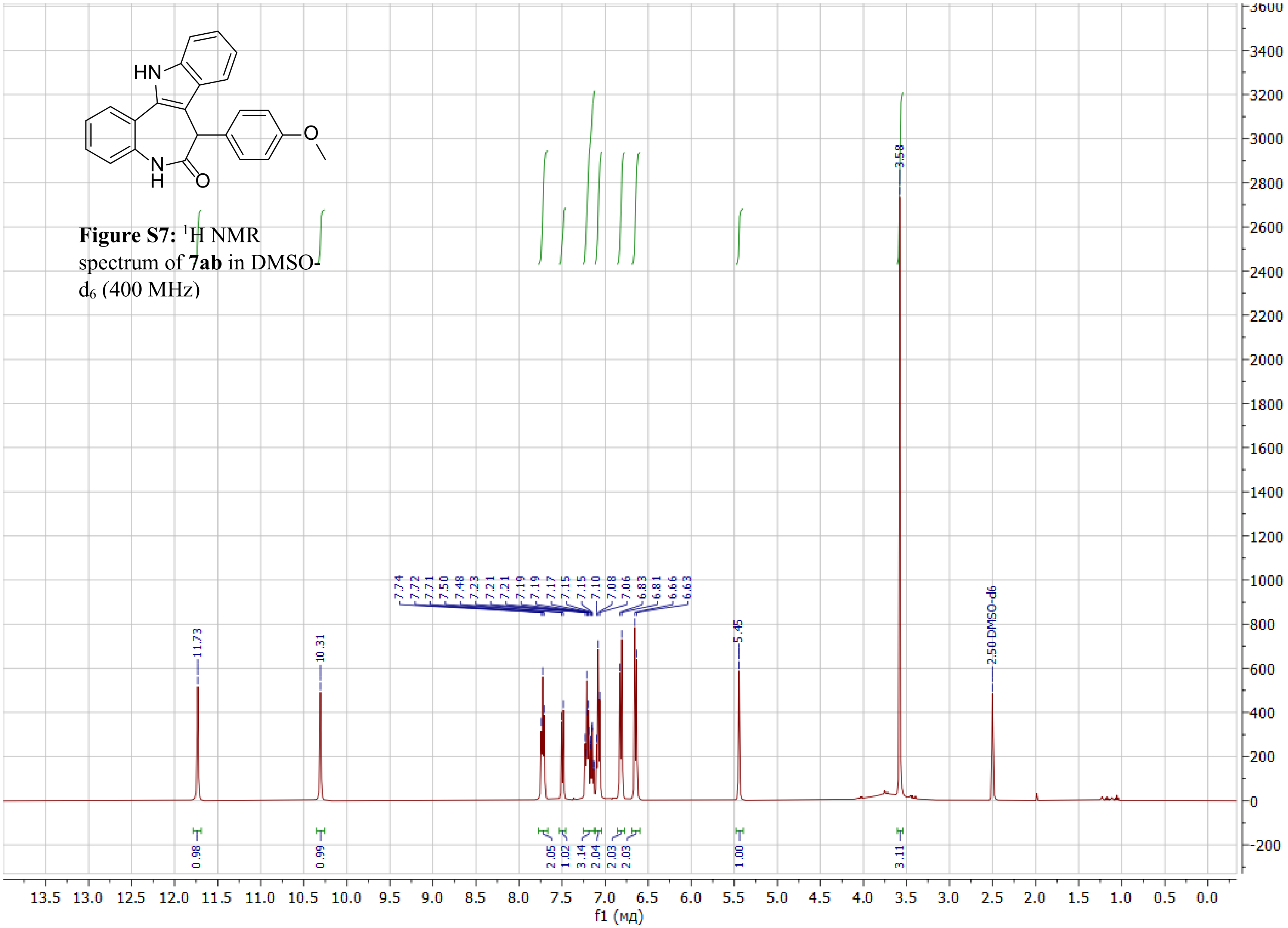

Figure S7: ^{1}H NMR spectrum of **7ab** in DMSO-d_6 (400 MHz)

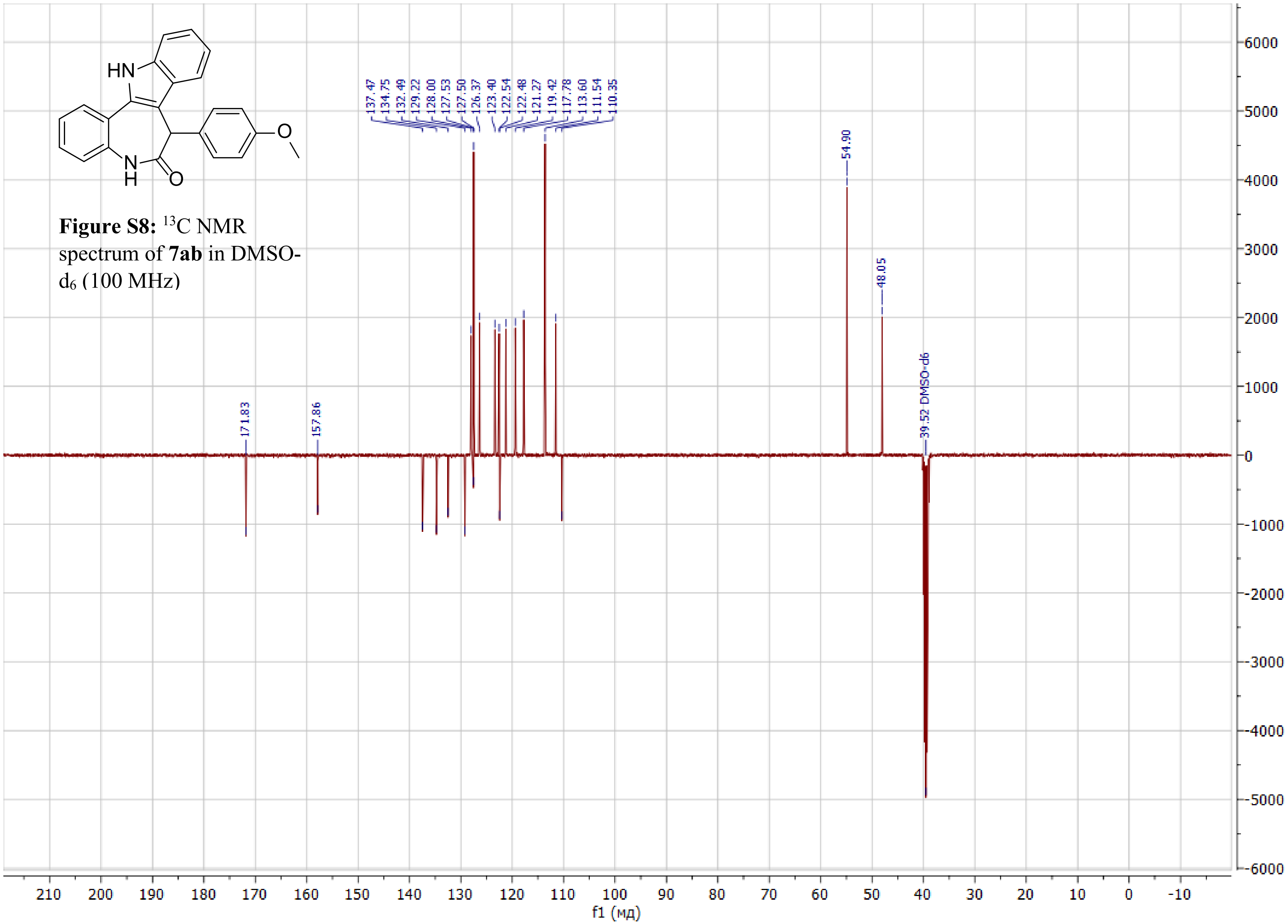

Figure S8: ^{13}C NMR spectrum of **7ab** in DMSO-d_6 (100 MHz)

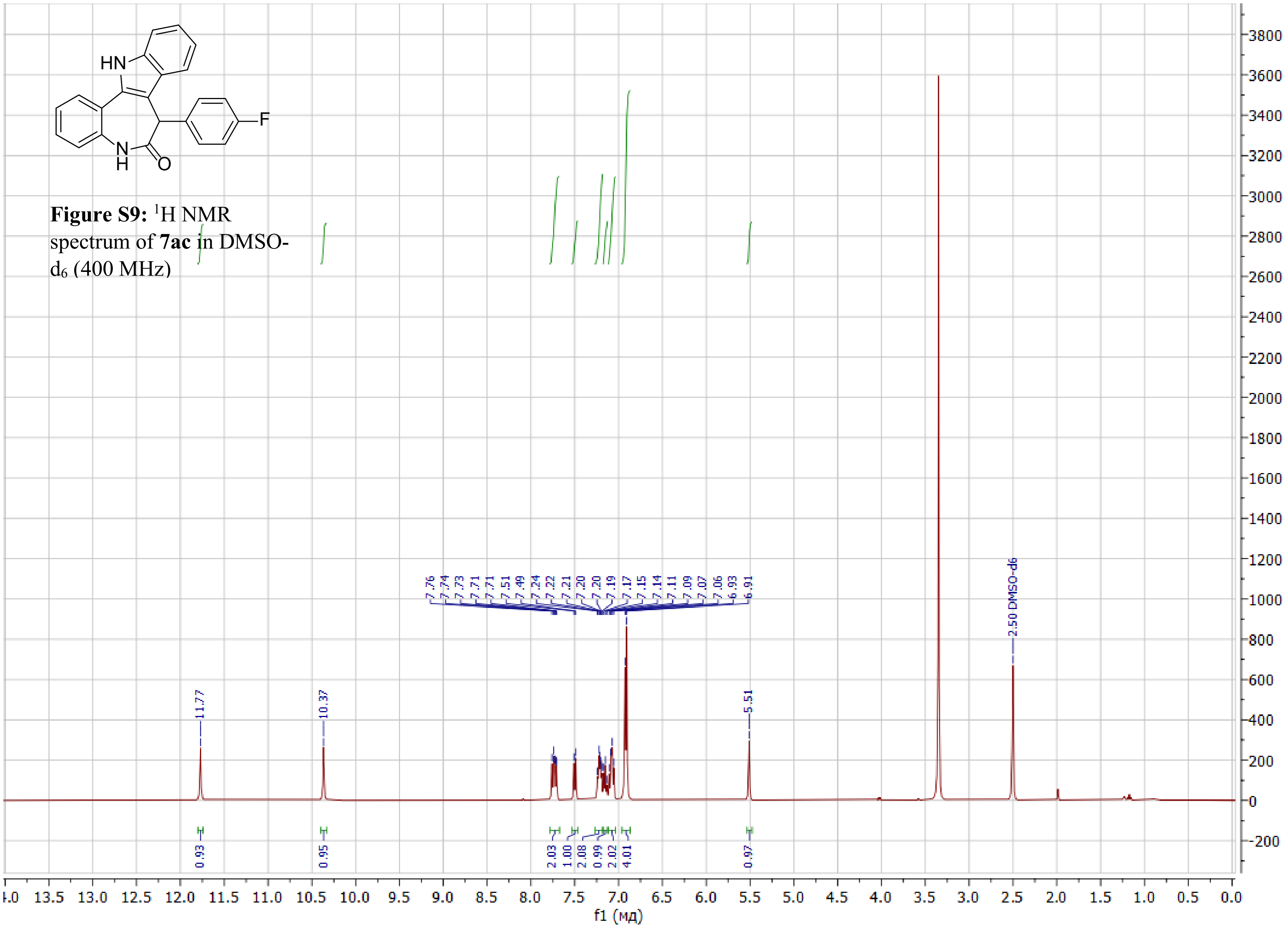

Figure S9: ^{1}H NMR spectrum of **7ac** in DMSO-d_6 (400 MHz)

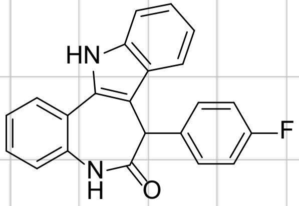

Figure S10: ^{13}C NMR spectrum of **7ac** in DMSO-d_6 (100 MHz)

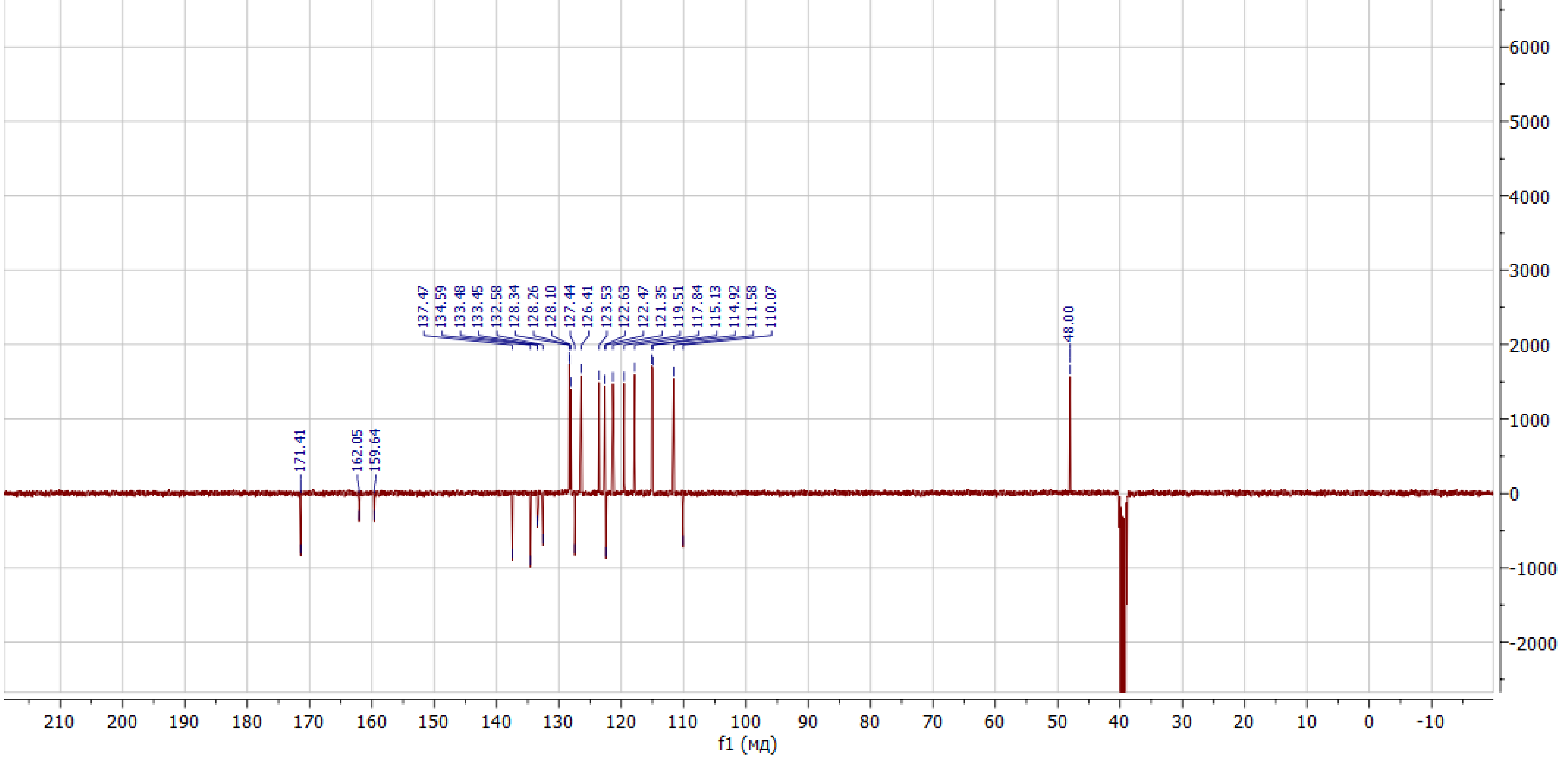

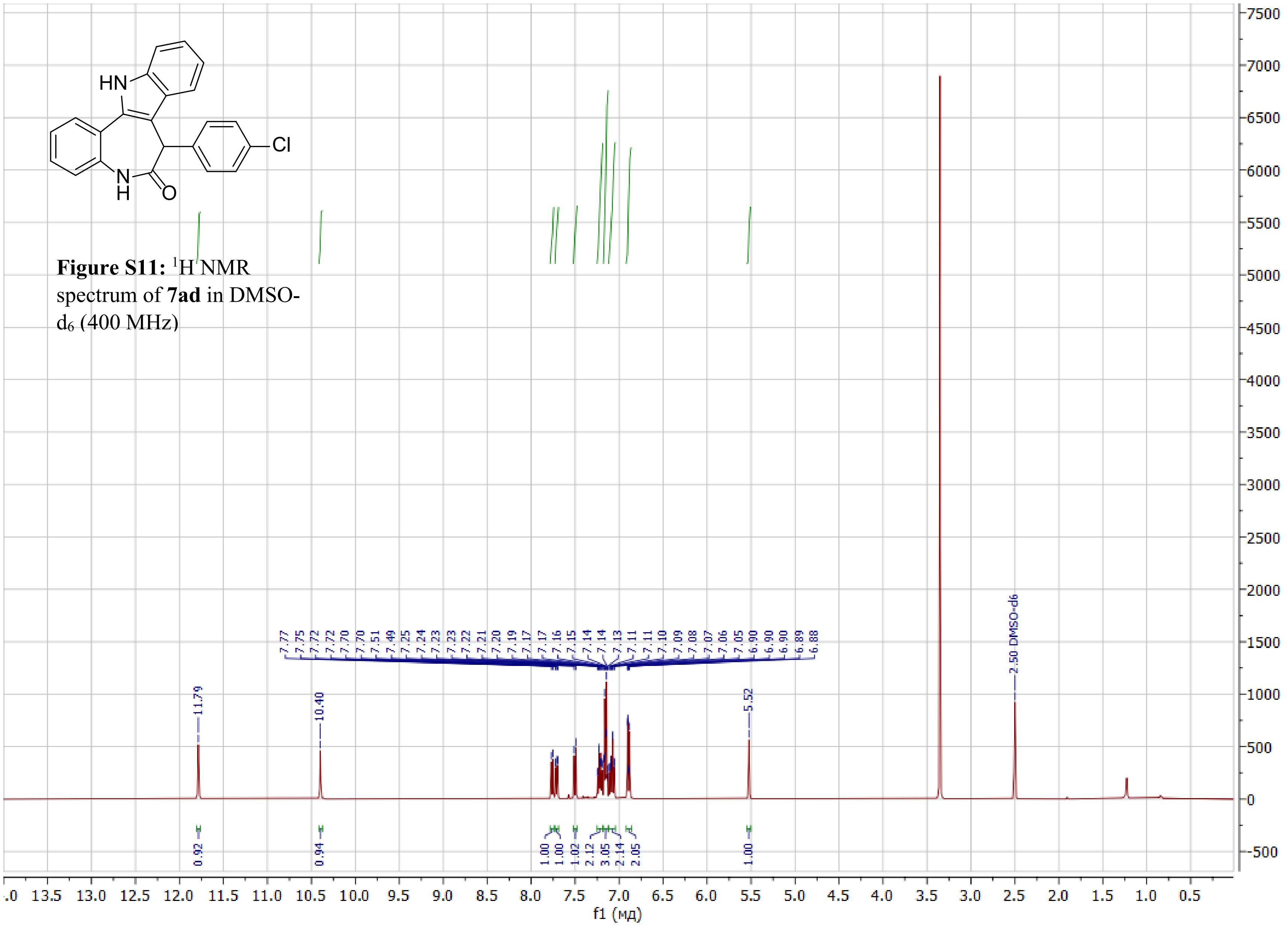

Figure S11: ^{1}H NMR spectrum of **7ad** in DMSO-d_6 (400 MHz)

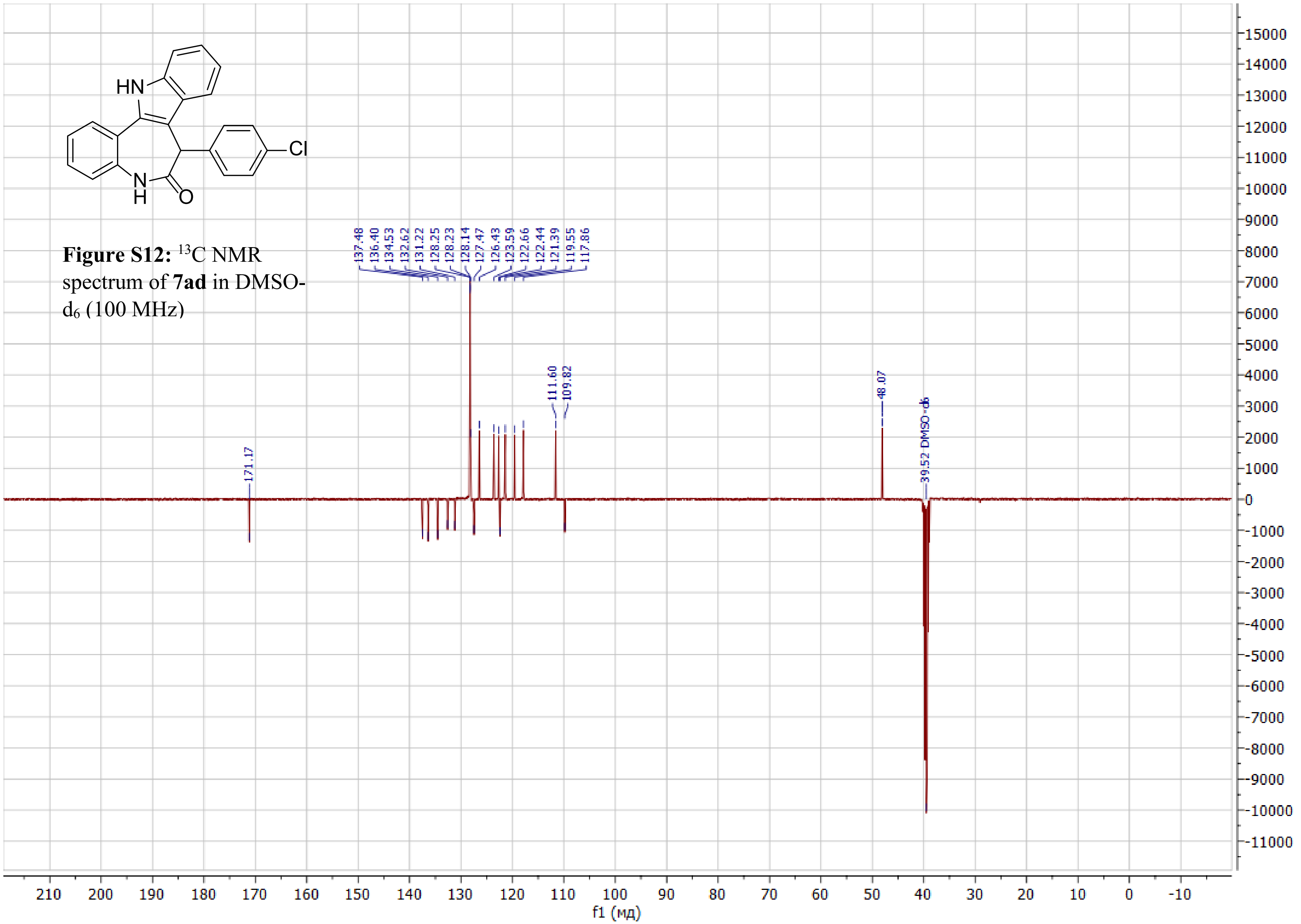

Figure S12: ^{13}C NMR spectrum of **7ad** in DMSO-d_6 (100 MHz)

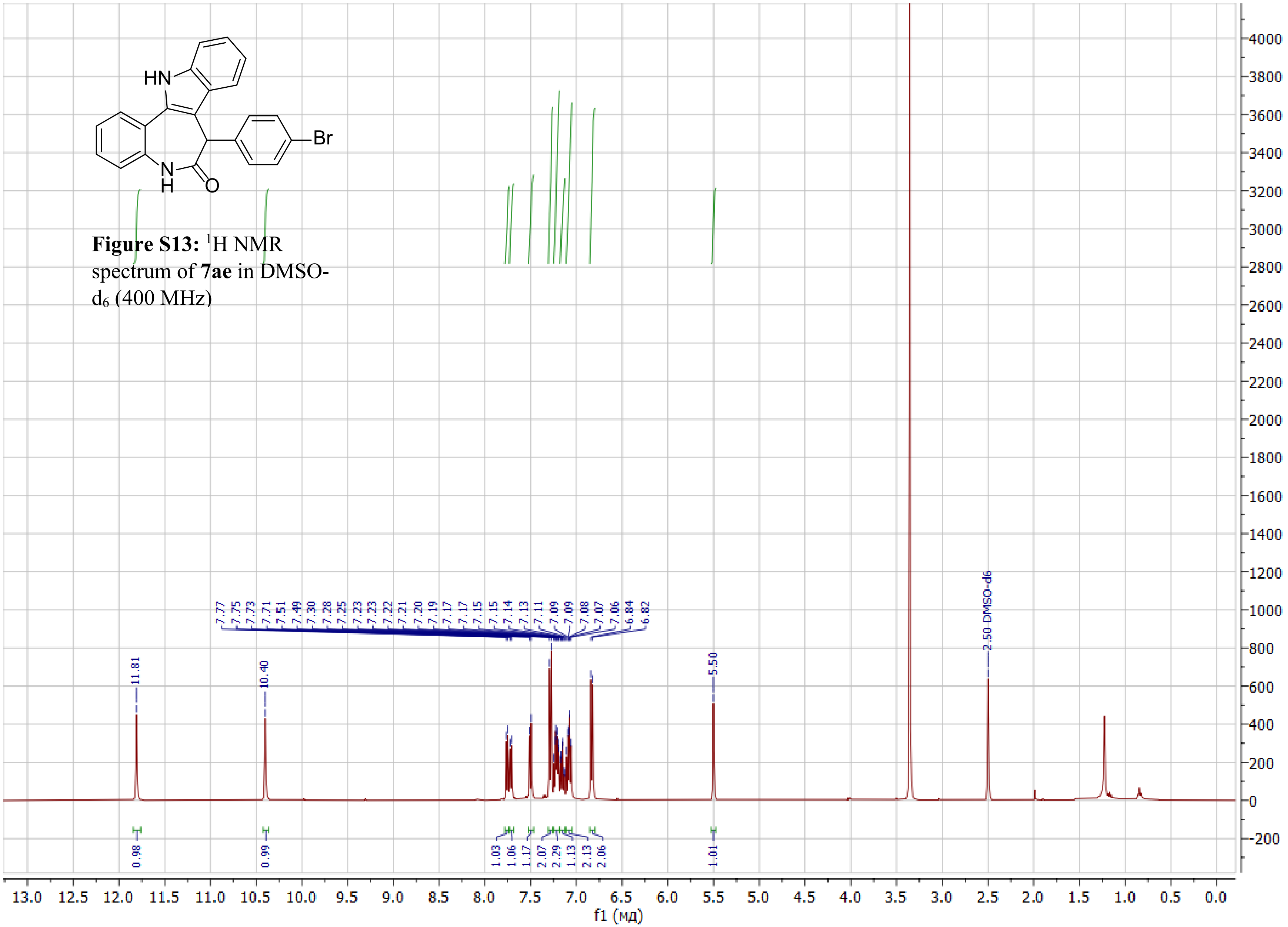

Figure S13: ^{1}H NMR spectrum of **7ae** in DMSO-d_6 (400 MHz)

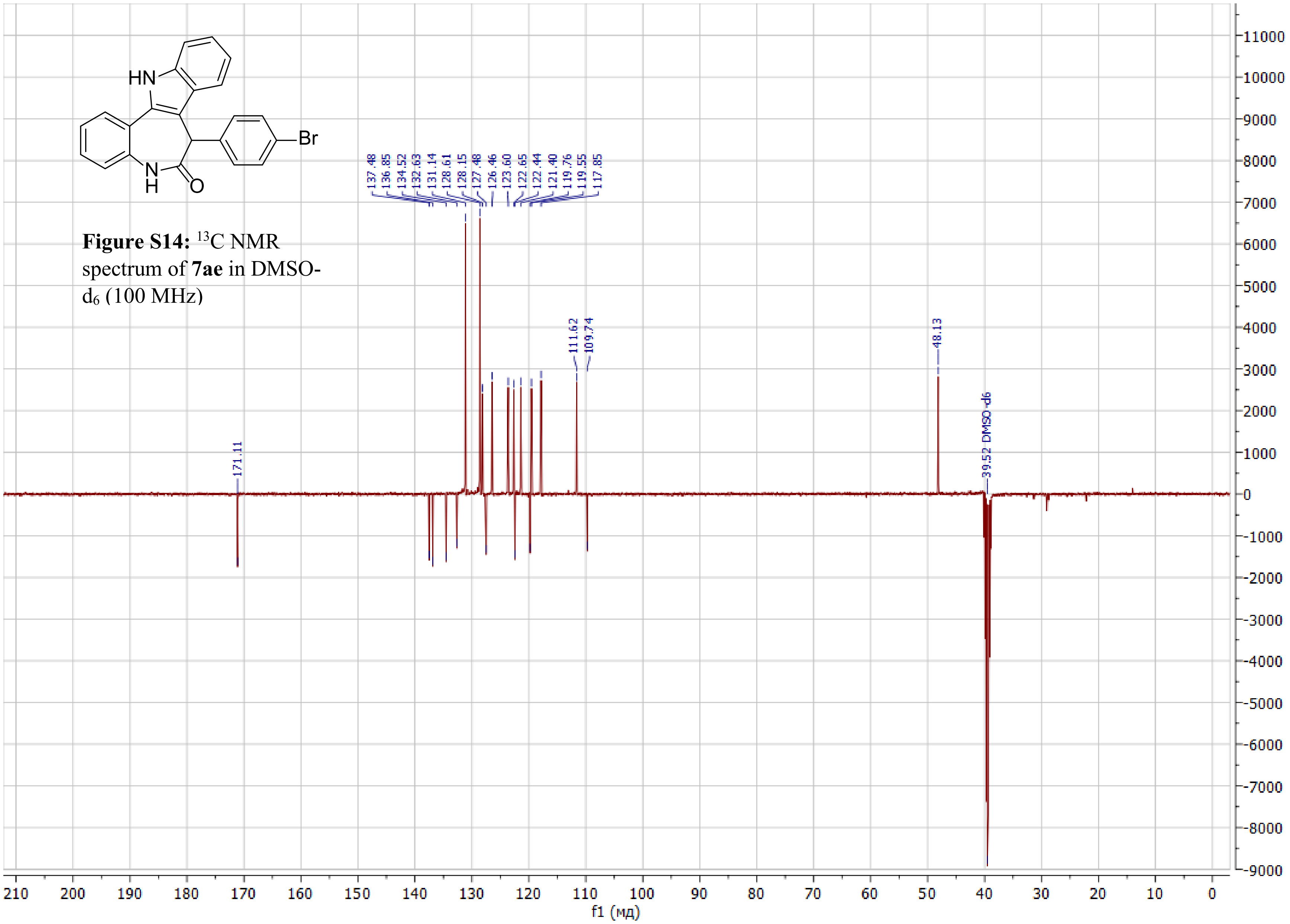

Figure S14: ^{13}C NMR spectrum of **7ae** in DMSO-d_6 (100 MHz)

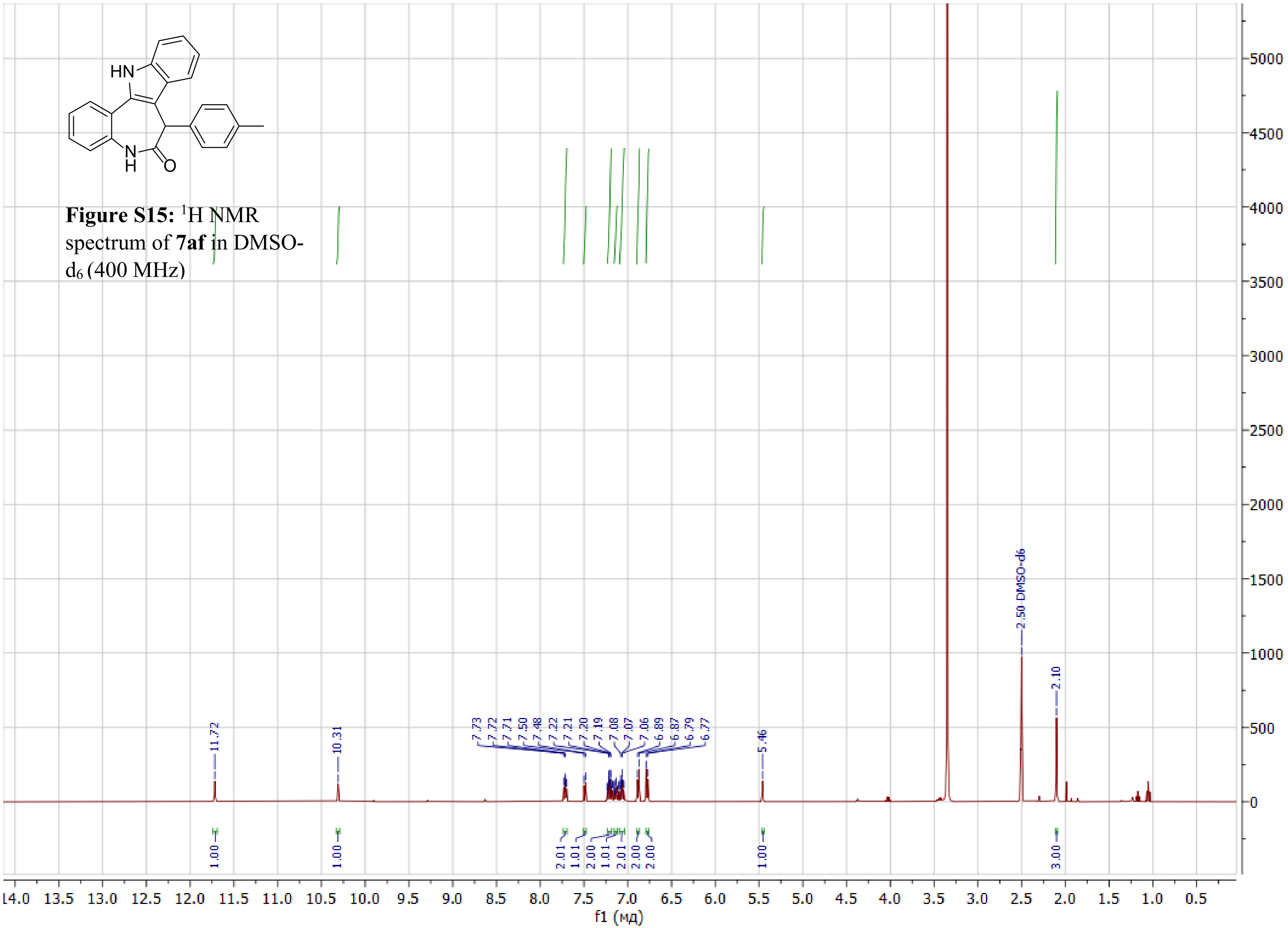

Figure S15: ^{1}H NMR spectrum of **7af** in DMSO-d_6 (400 MHz)

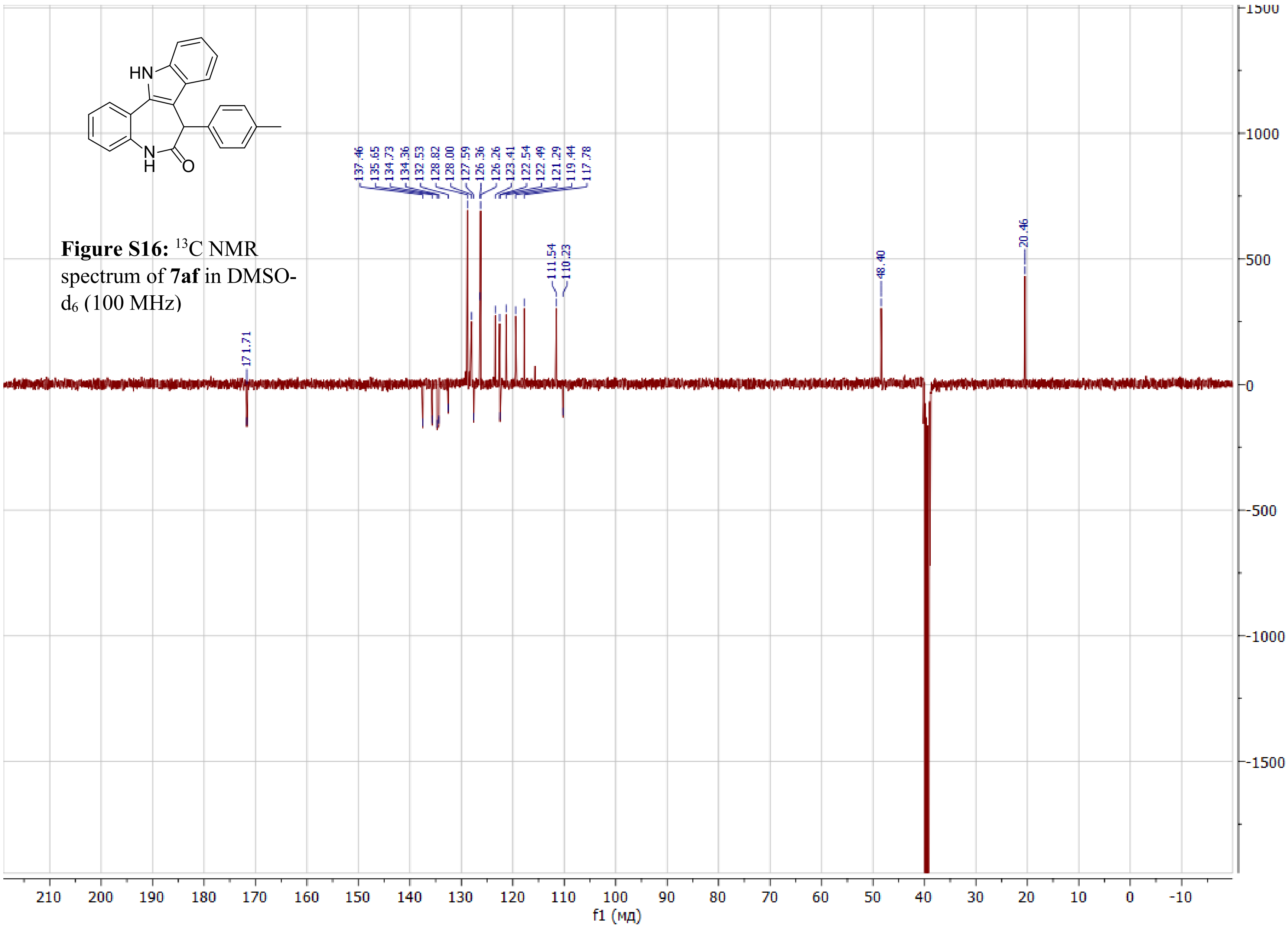

Figure S16: ^{13}C NMR spectrum of **7af** in DMSO-d_6 (100 MHz)

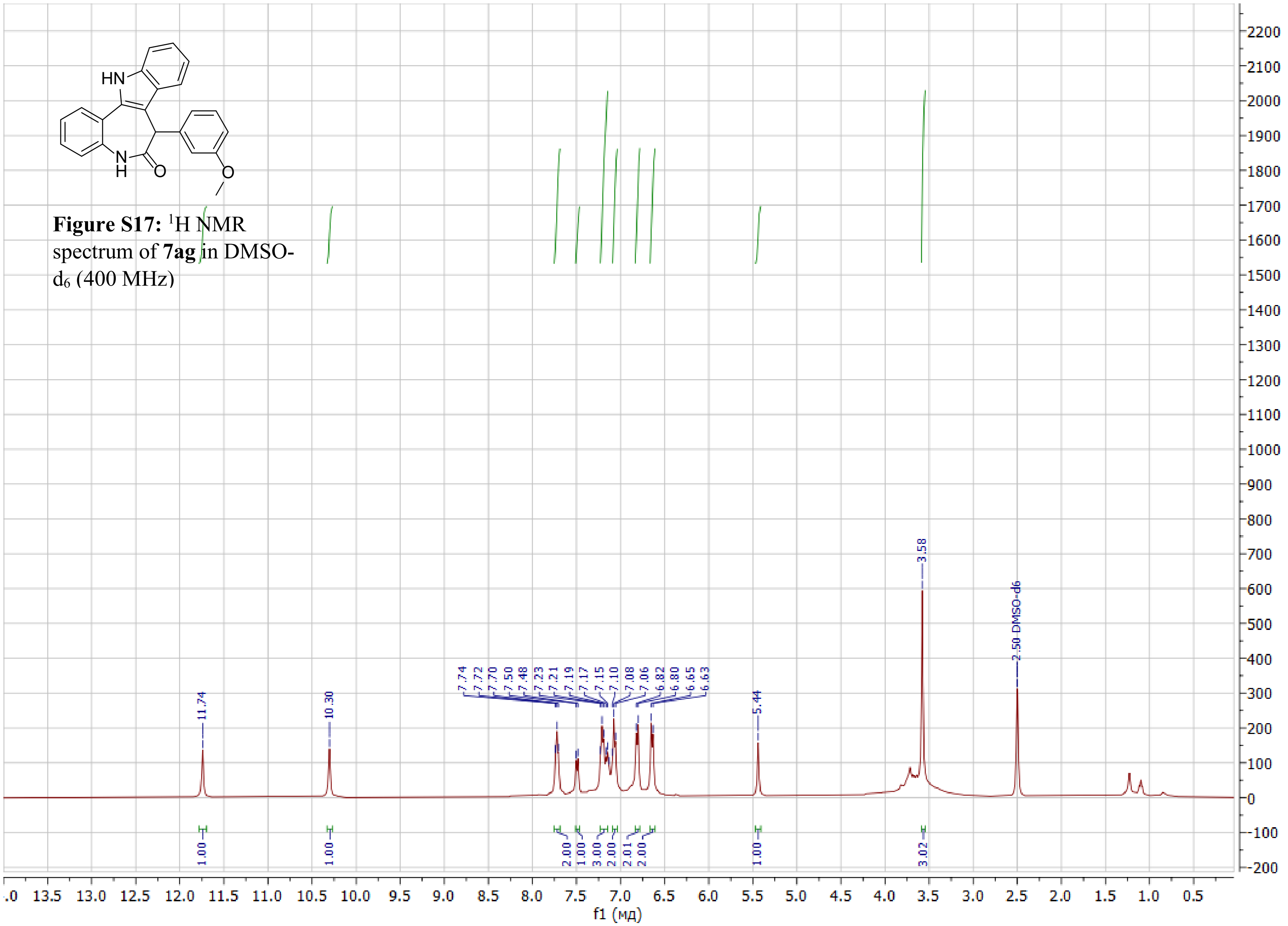

Figure S17: ^{1}H NMR spectrum of **7ag** in DMSO-d_6 (400 MHz)

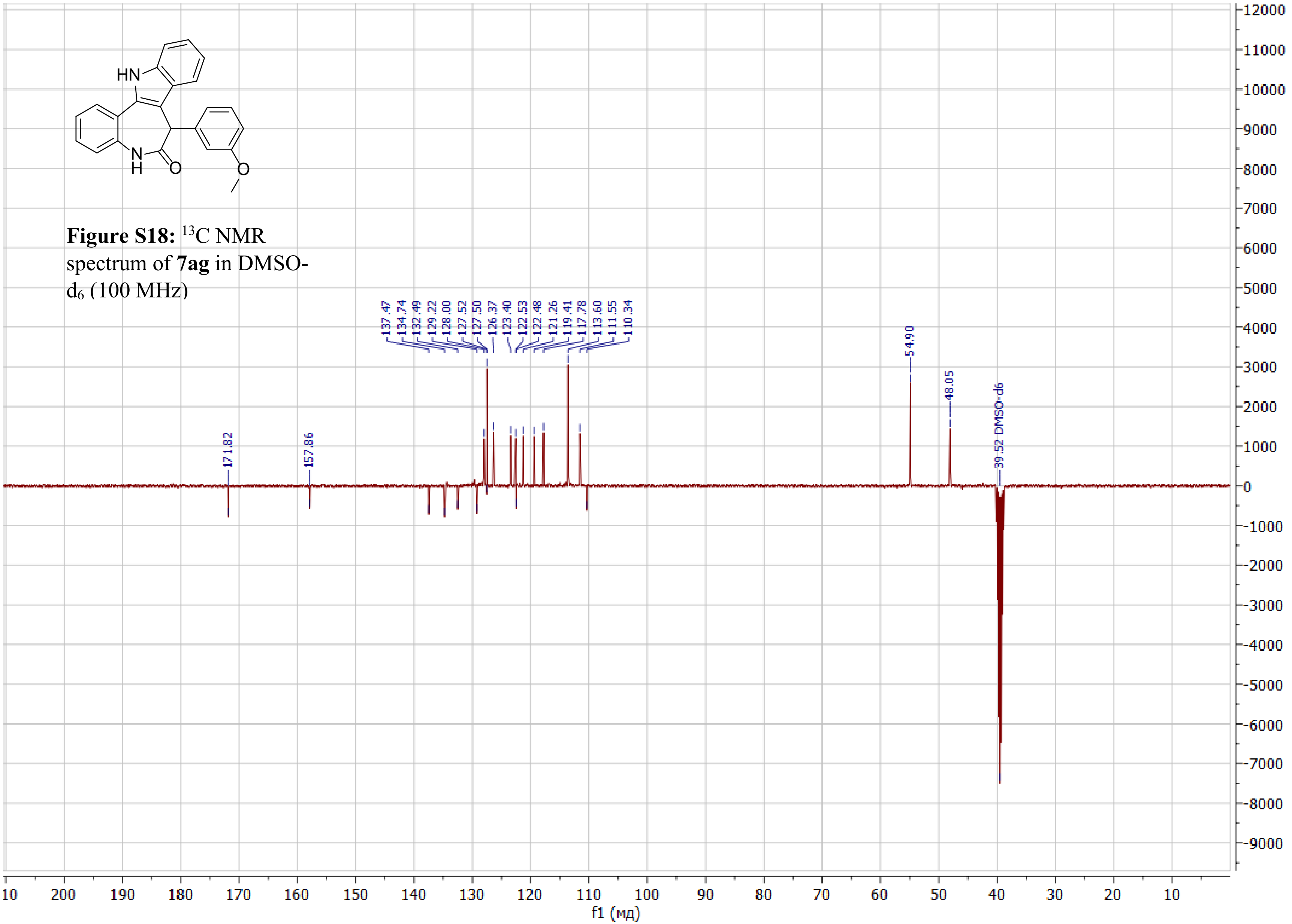

Figure S18: ^{13}C NMR spectrum of **7ag** in DMSO-d_6 (100 MHz)

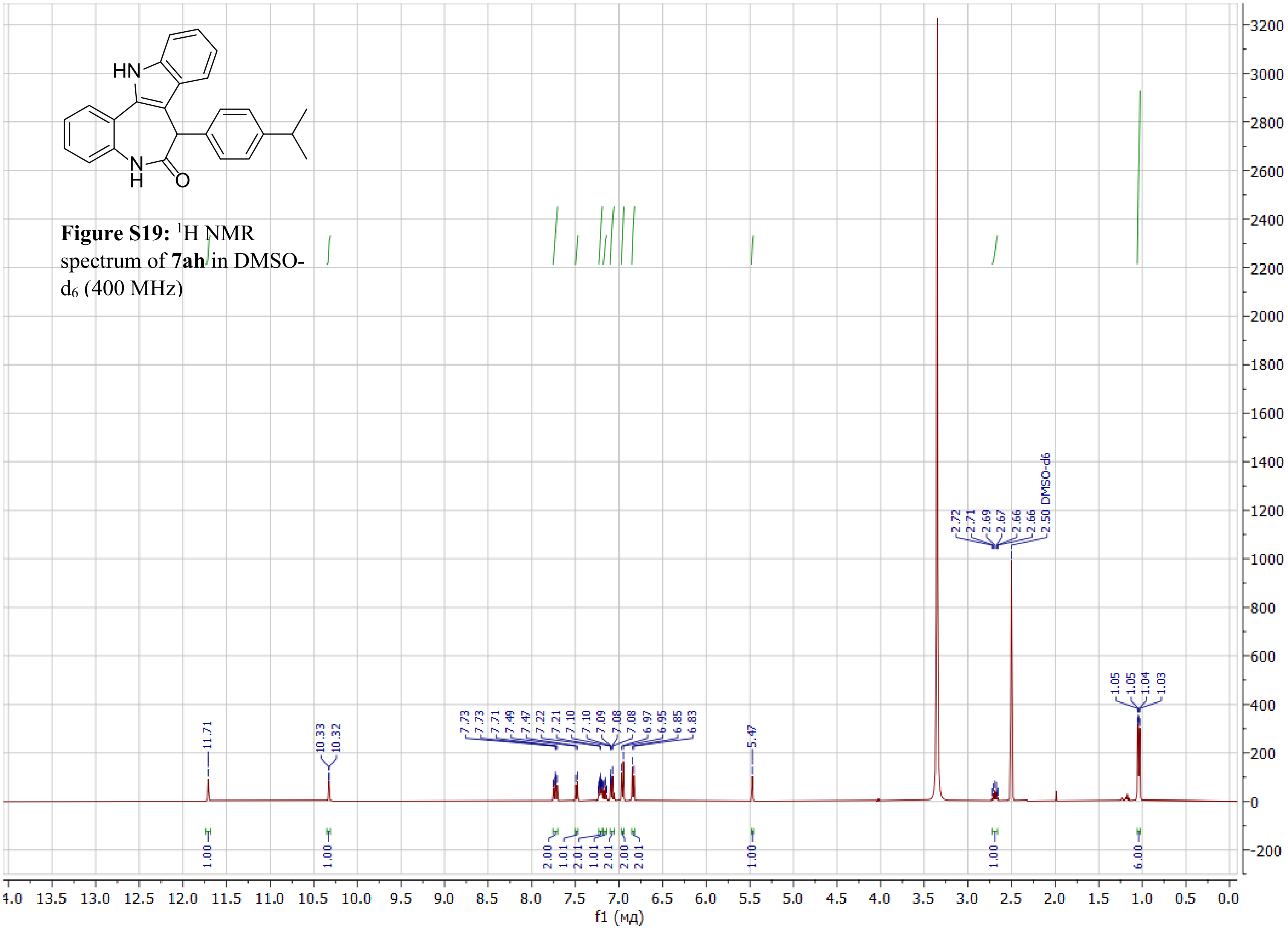

Figure S19: ^{1}H NMR spectrum of **7ah** in DMSO-d_6 (400 MHz)

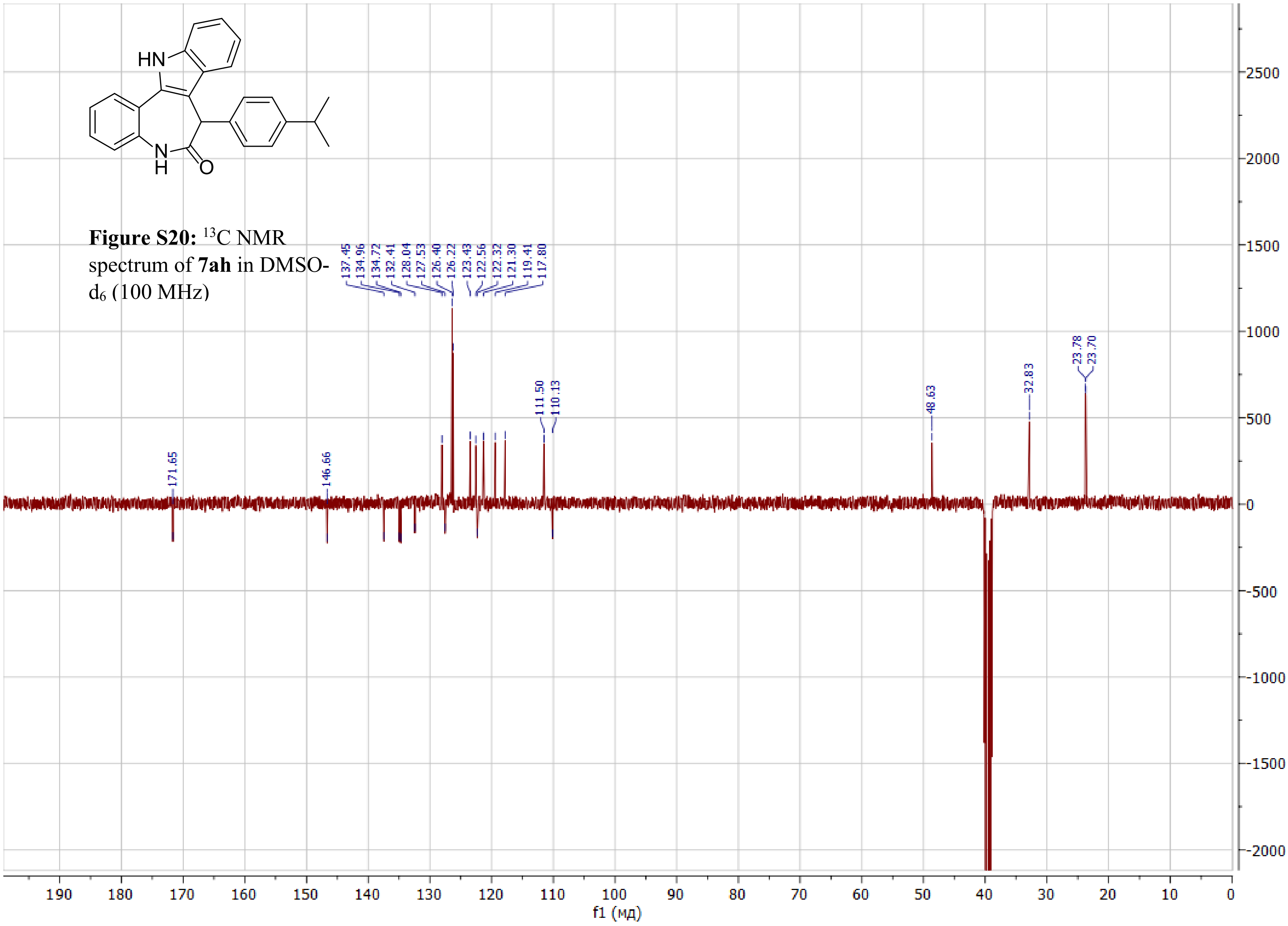

Figure S20: ^{13}C NMR spectrum of **7ah** in DMSO-d_6 (100 MHz)

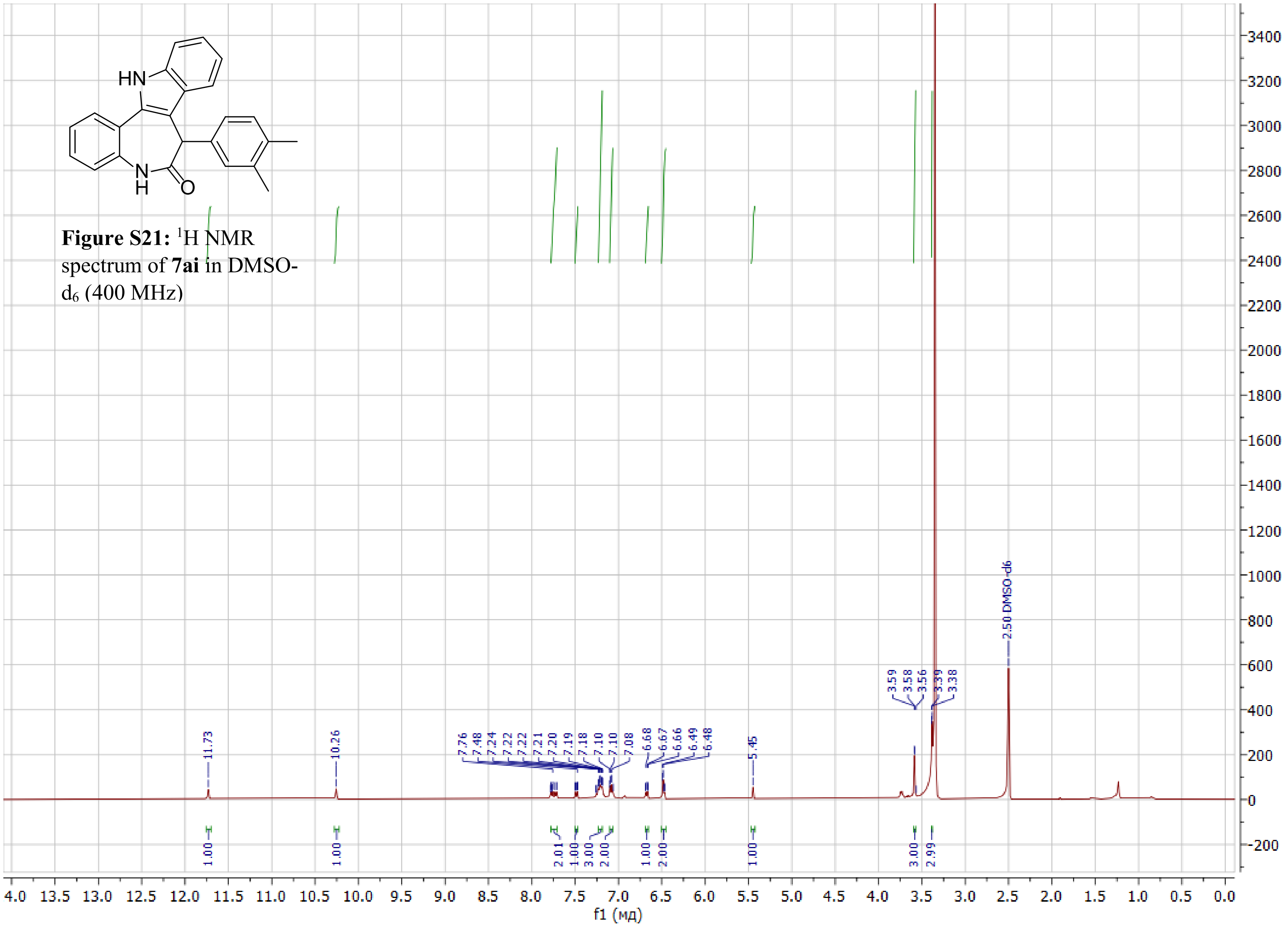

Figure S21: ^{1}H NMR spectrum of **7ai** in DMSO-d_6 (400 MHz)

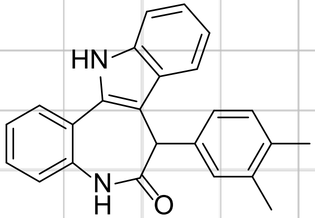

Figure S22: ^{13}C NMR spectrum of **7ai** in DMSO-d_6 (100 MHz)

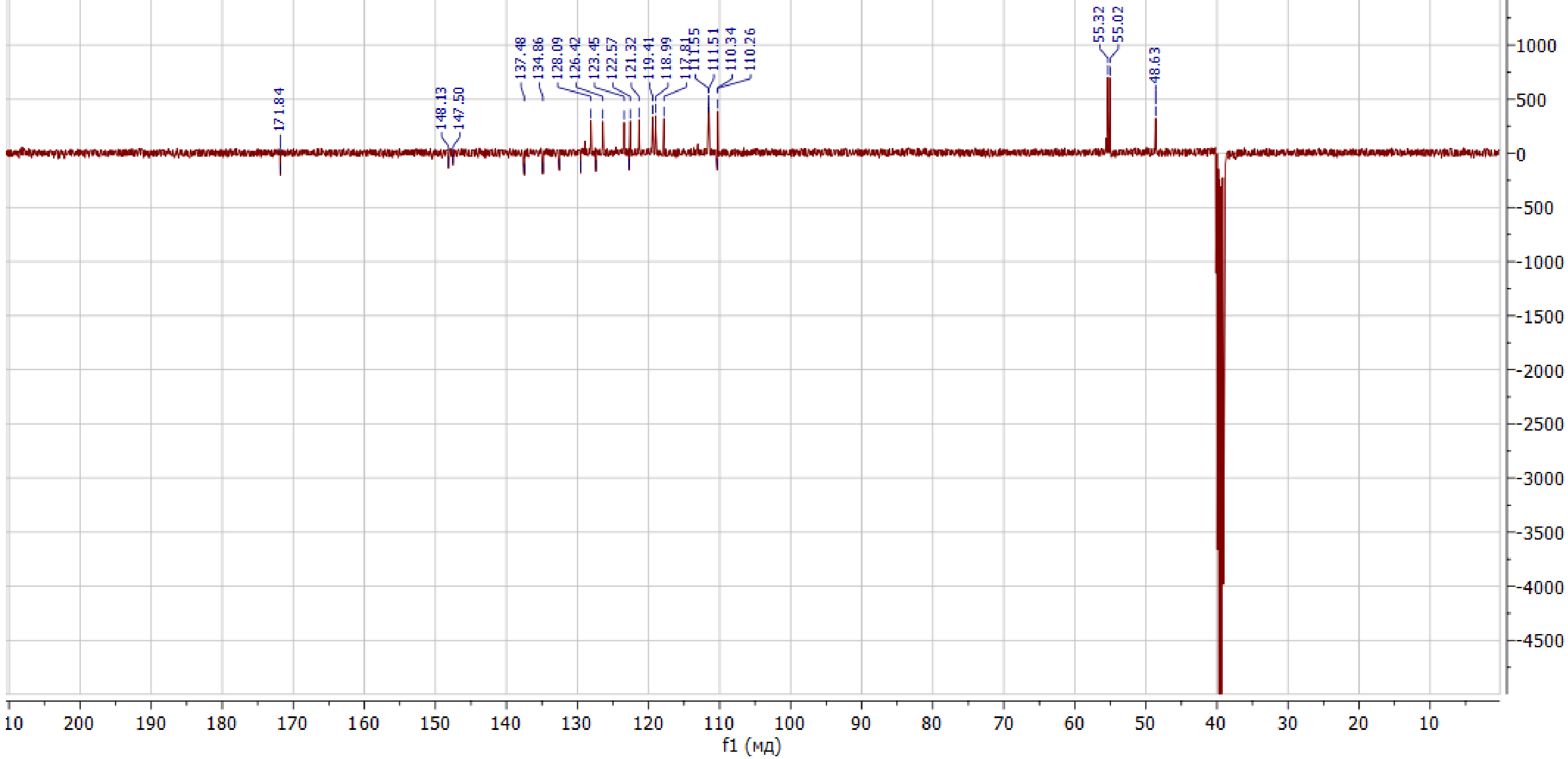

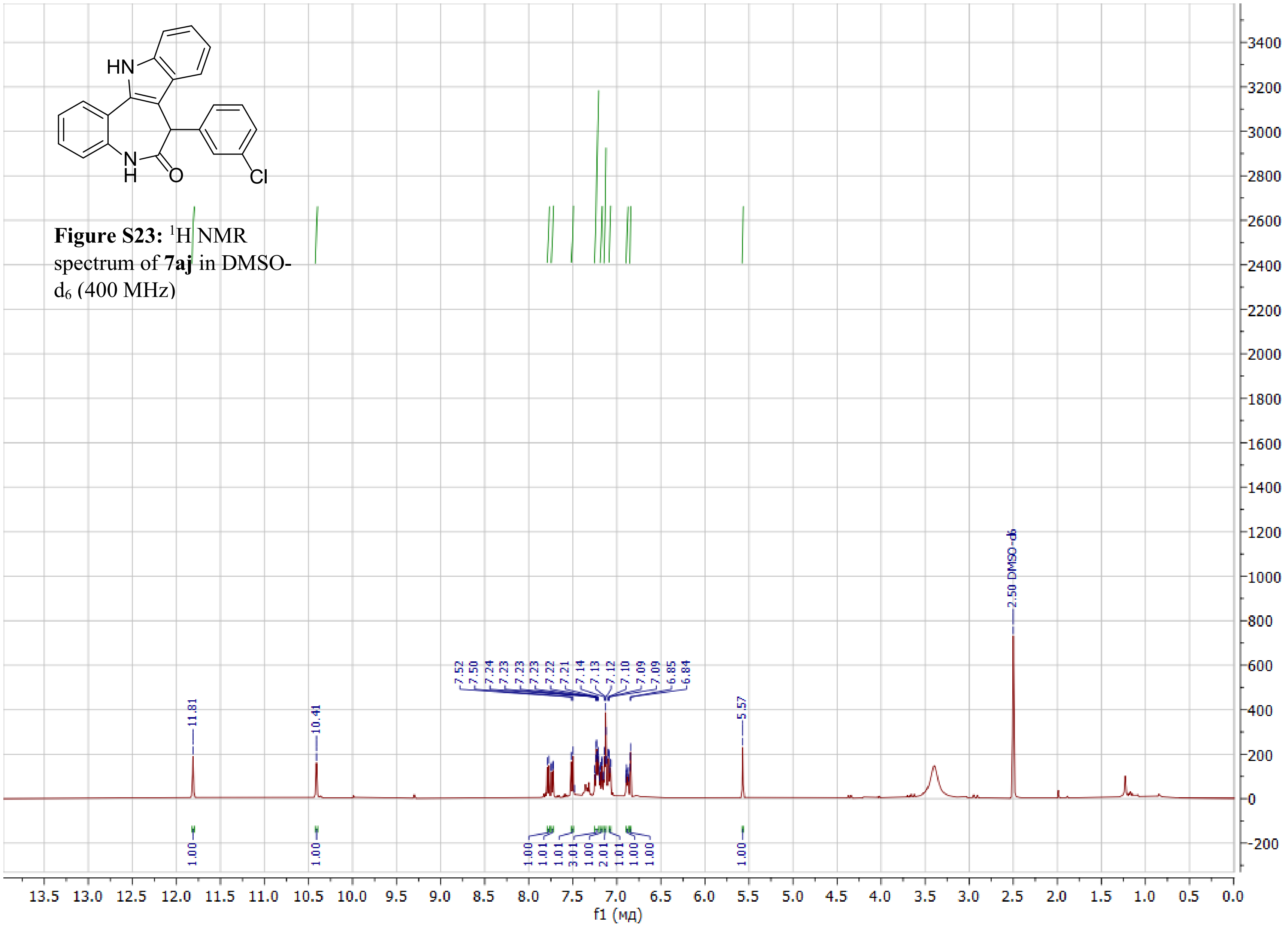

Figure S23: ^{1}H NMR spectrum of **7aj** in DMSO-d_6 (400 MHz)

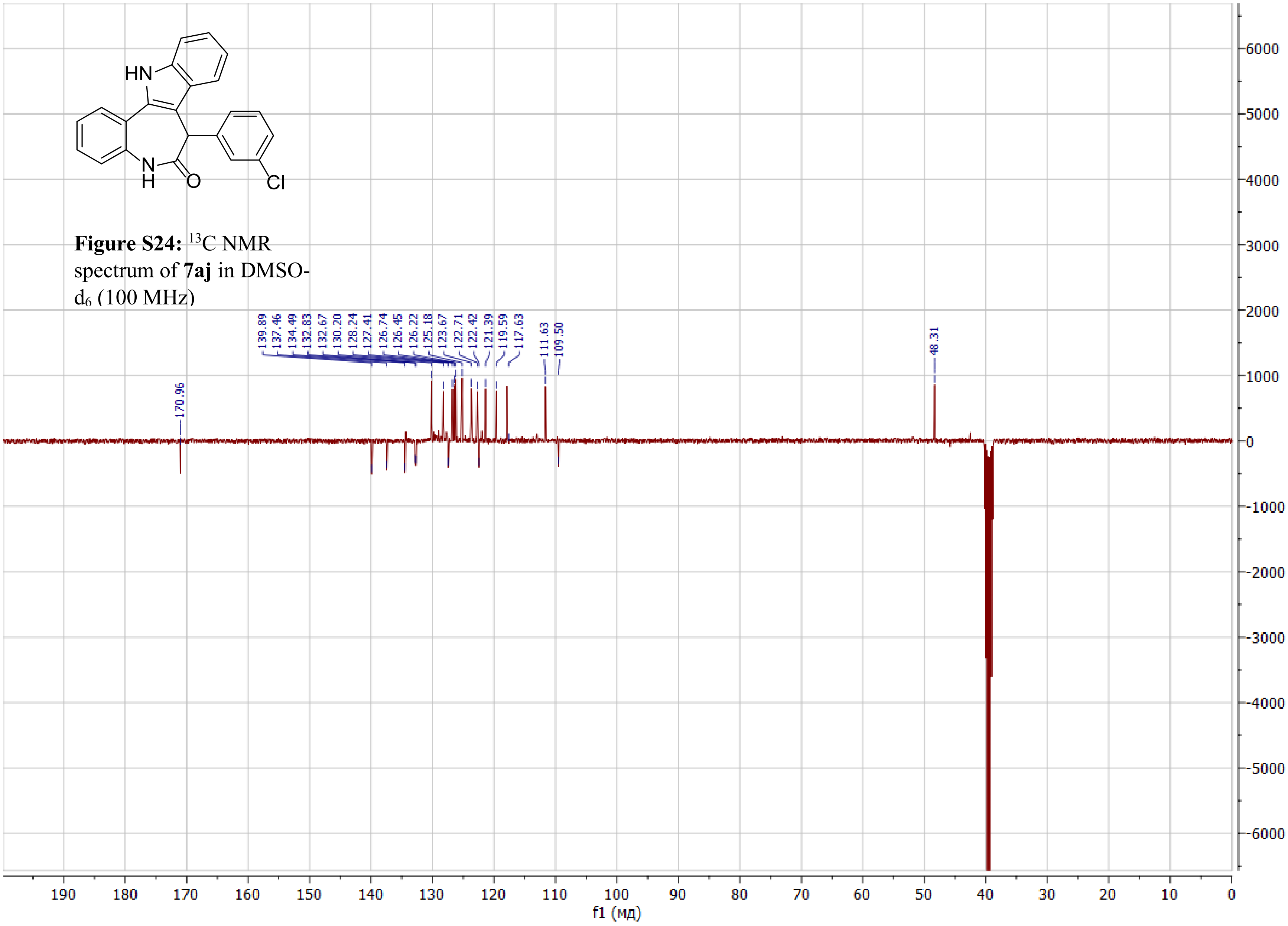

Figure S24: ^{13}C NMR spectrum of **7aj** in DMSO-d_6 (100 MHz)

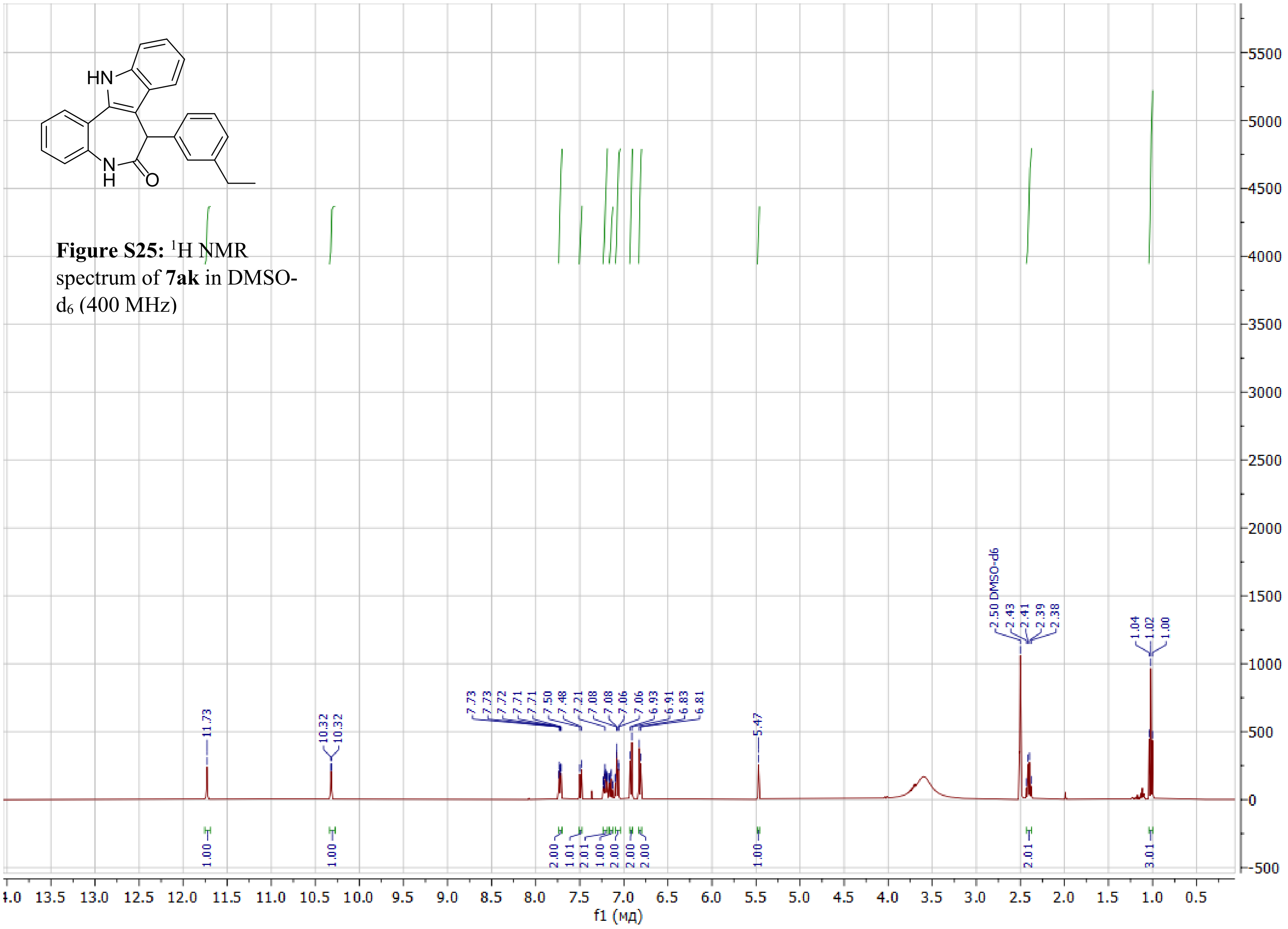

Figure S25: ^{1}H NMR spectrum of **7ak** in DMSO-d_6 (400 MHz)

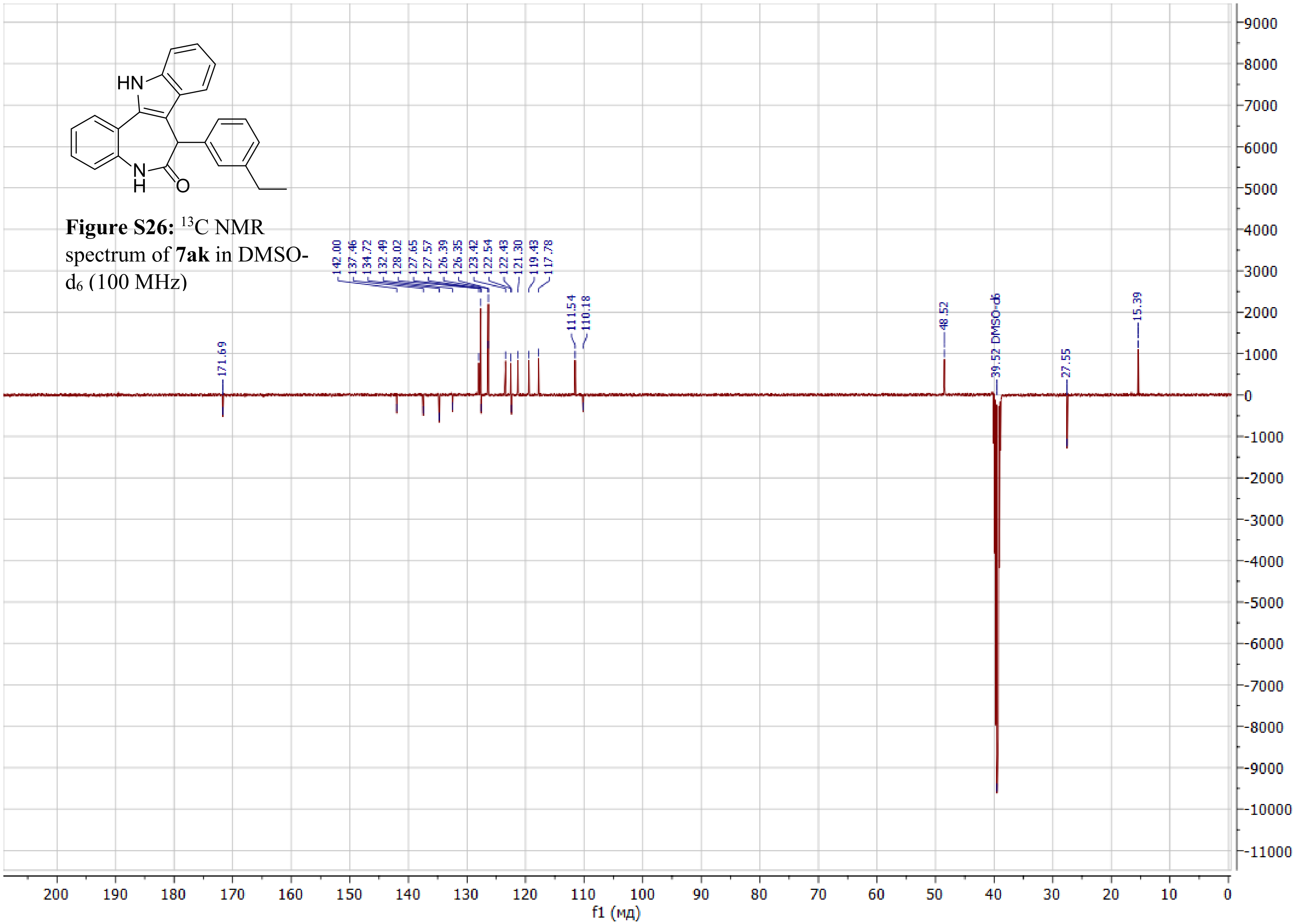

Figure S26: ^{13}C NMR spectrum of **7ak** in DMSO-d_6 (100 MHz)

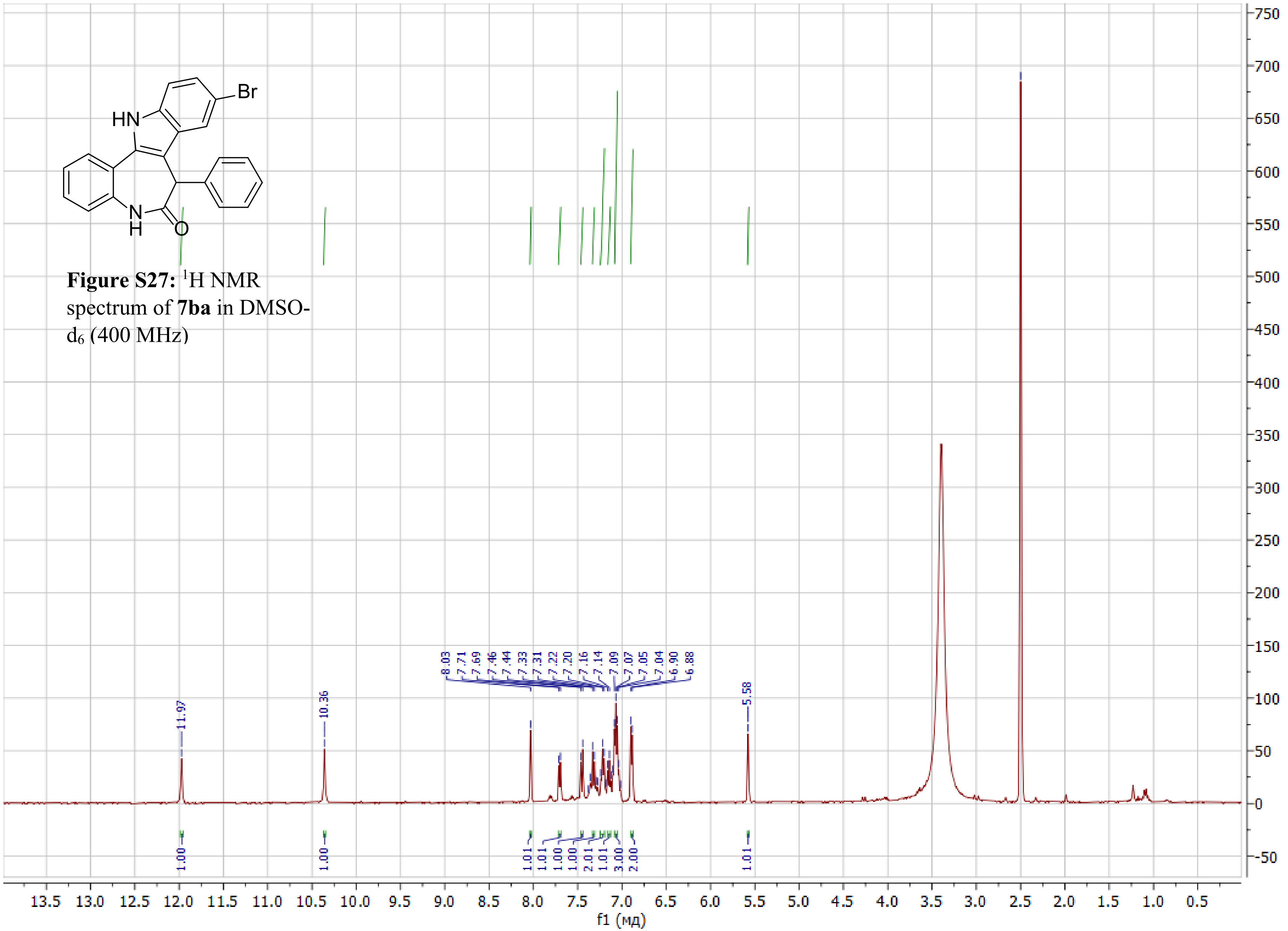

Figure S27: ^{1}H NMR spectrum of **7ba** in DMSO-d_6 (400 MHz)

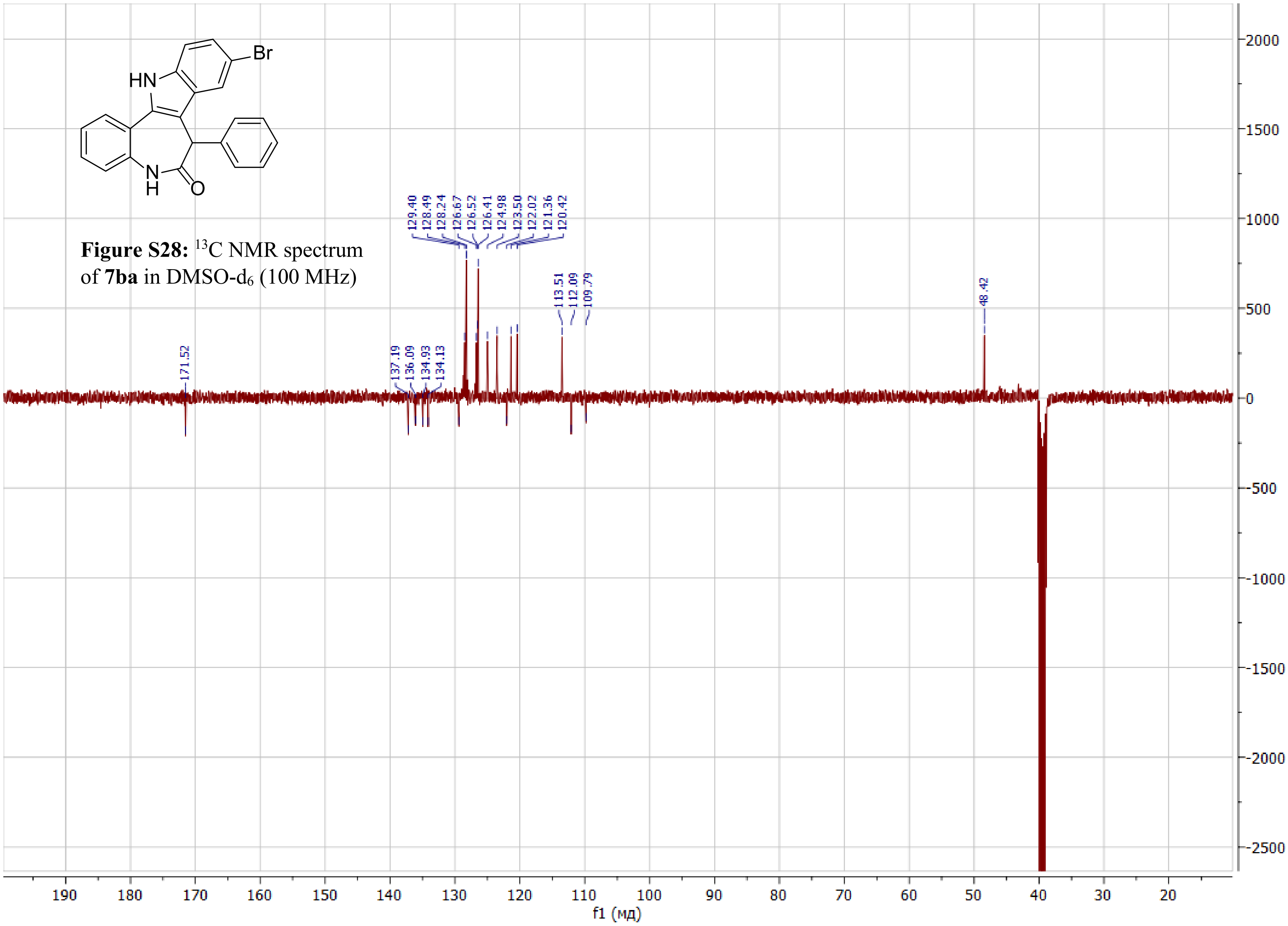

Figure S28: ^{13}C NMR spectrum of **7ba** in DMSO-d_6 (100 MHz)

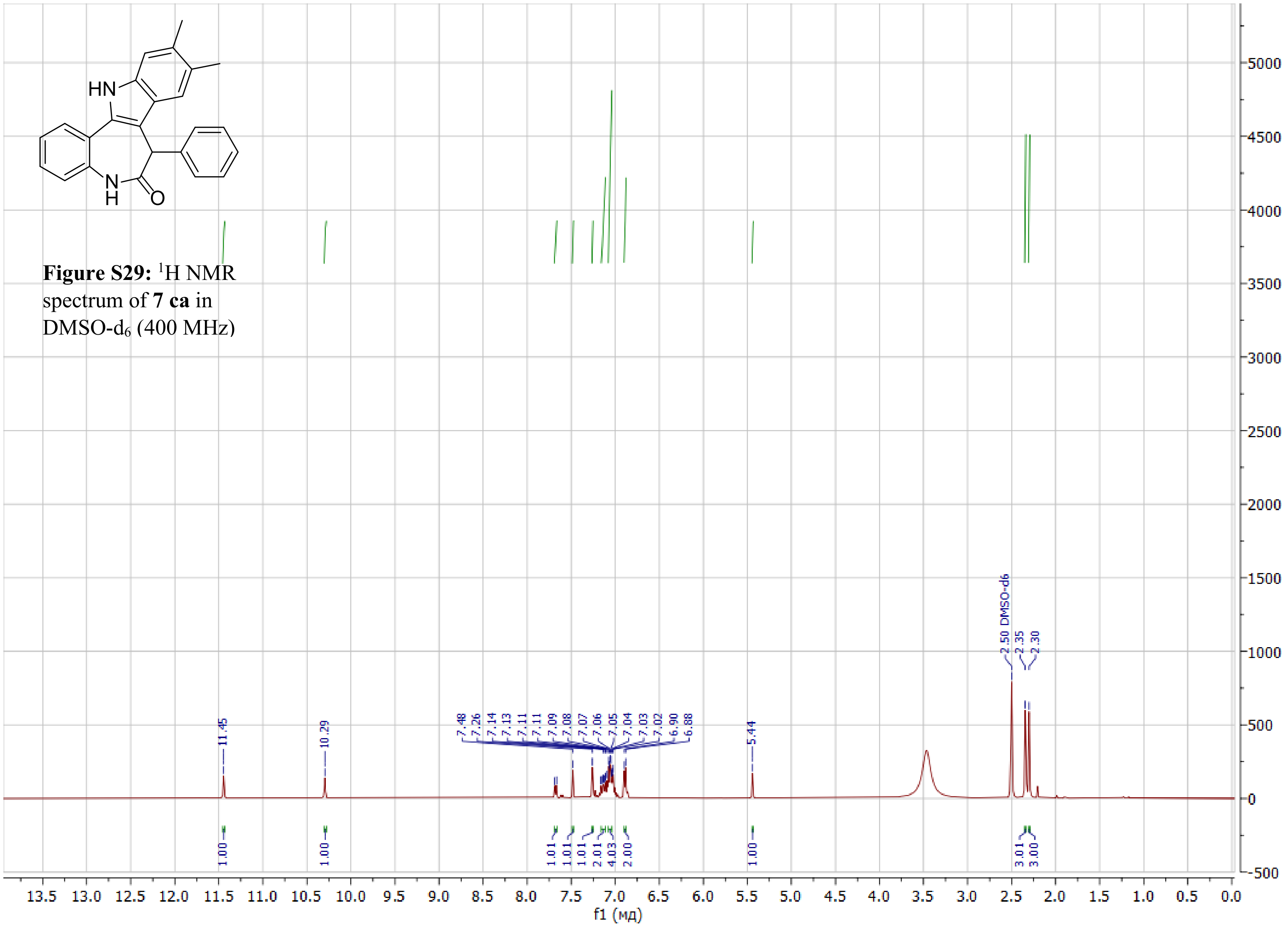

Figure S29: ^{1}H NMR spectrum of **7 ca** in DMSO-d_6 (400 MHz)

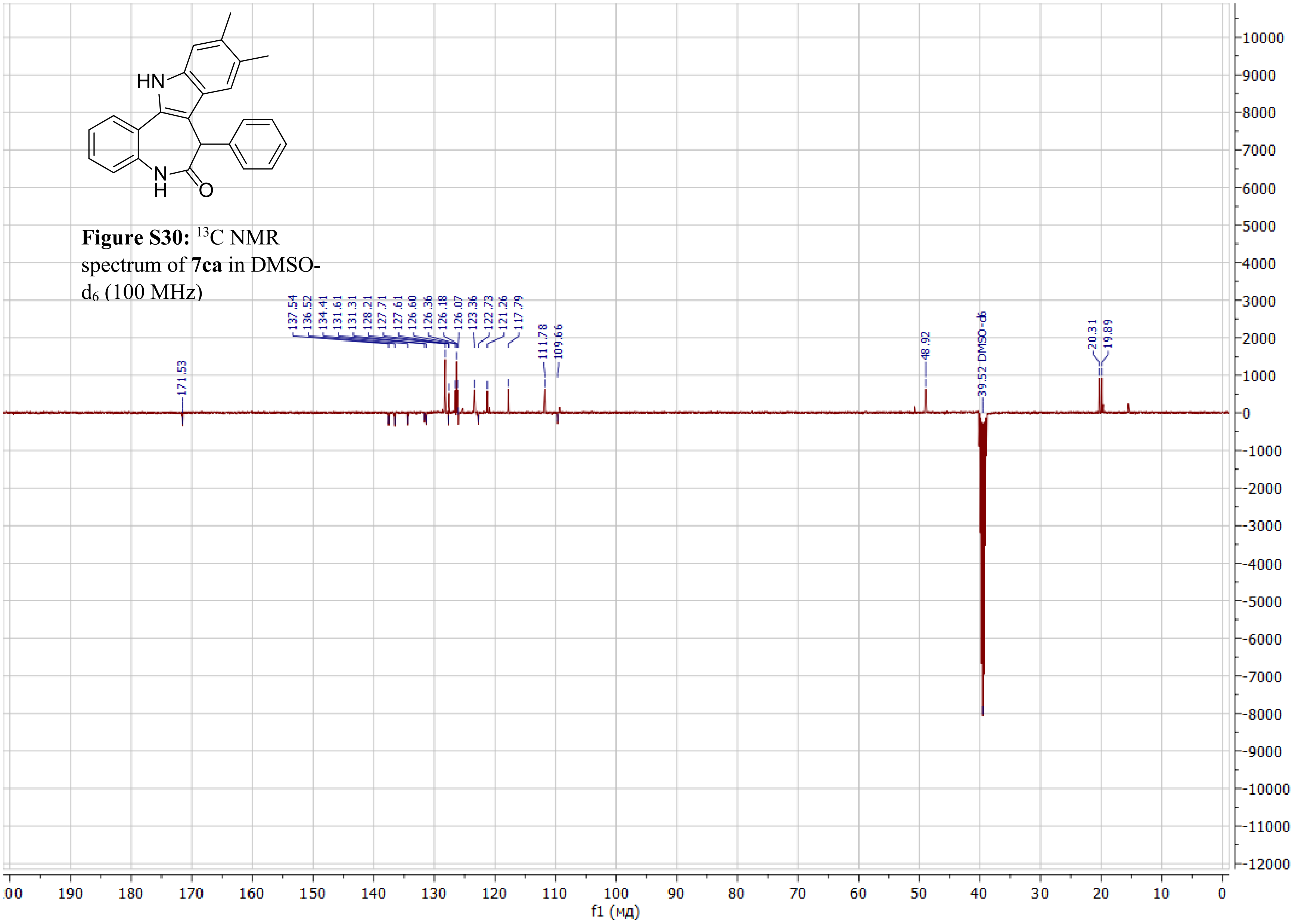

Figure S30: ^{13}C NMR spectrum of **7ca** in DMSO-d_6 (100 MHz)

^{1}H and ^{13}C NMR spectral charts for 4-(2-aminophenyl)-4-oxo-2-arylbutanoic acids **15a-k**

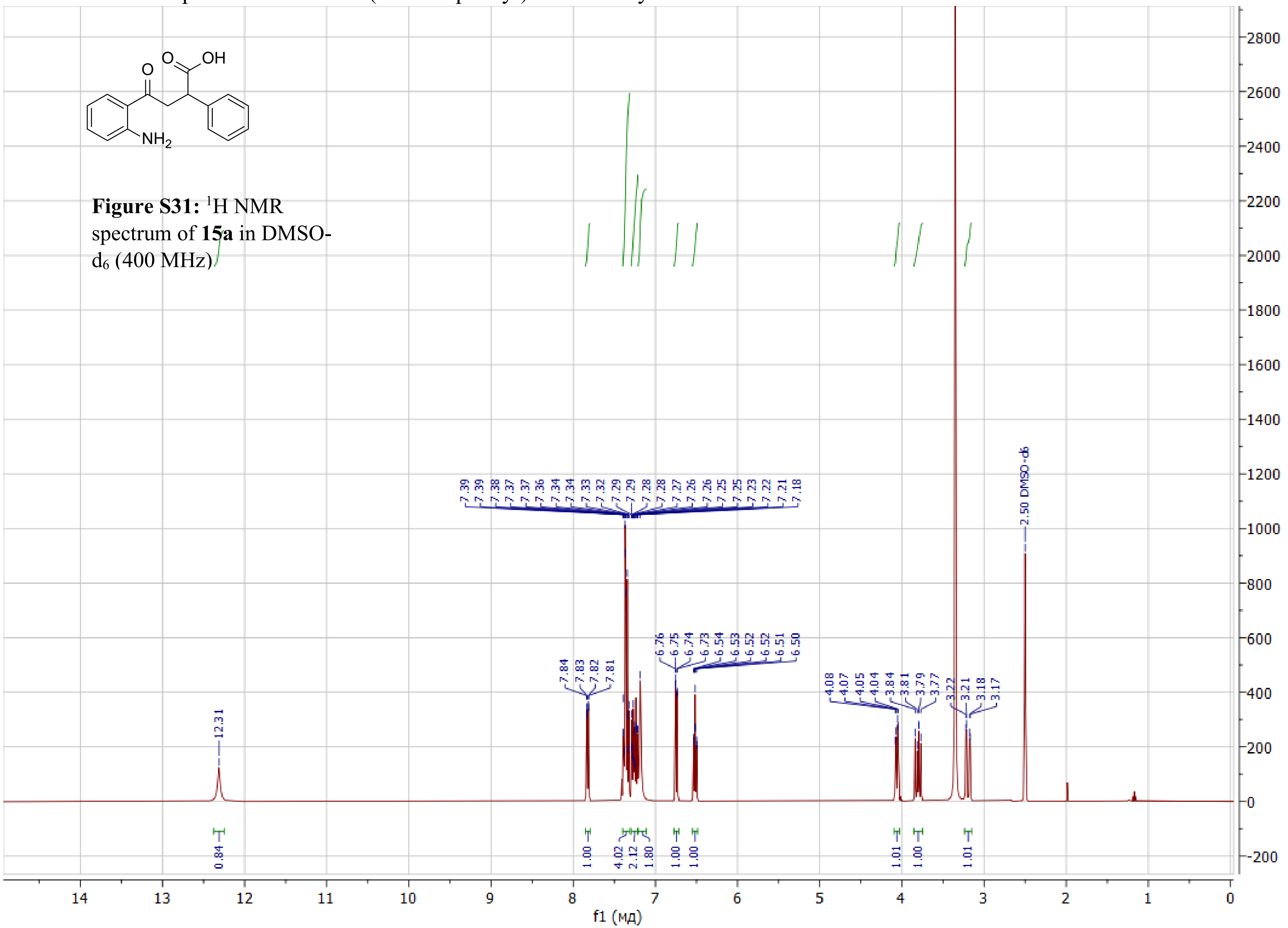

Figure S31: ^{1}H NMR spectrum of **15a** in DMSO-d_6 (400 MHz)

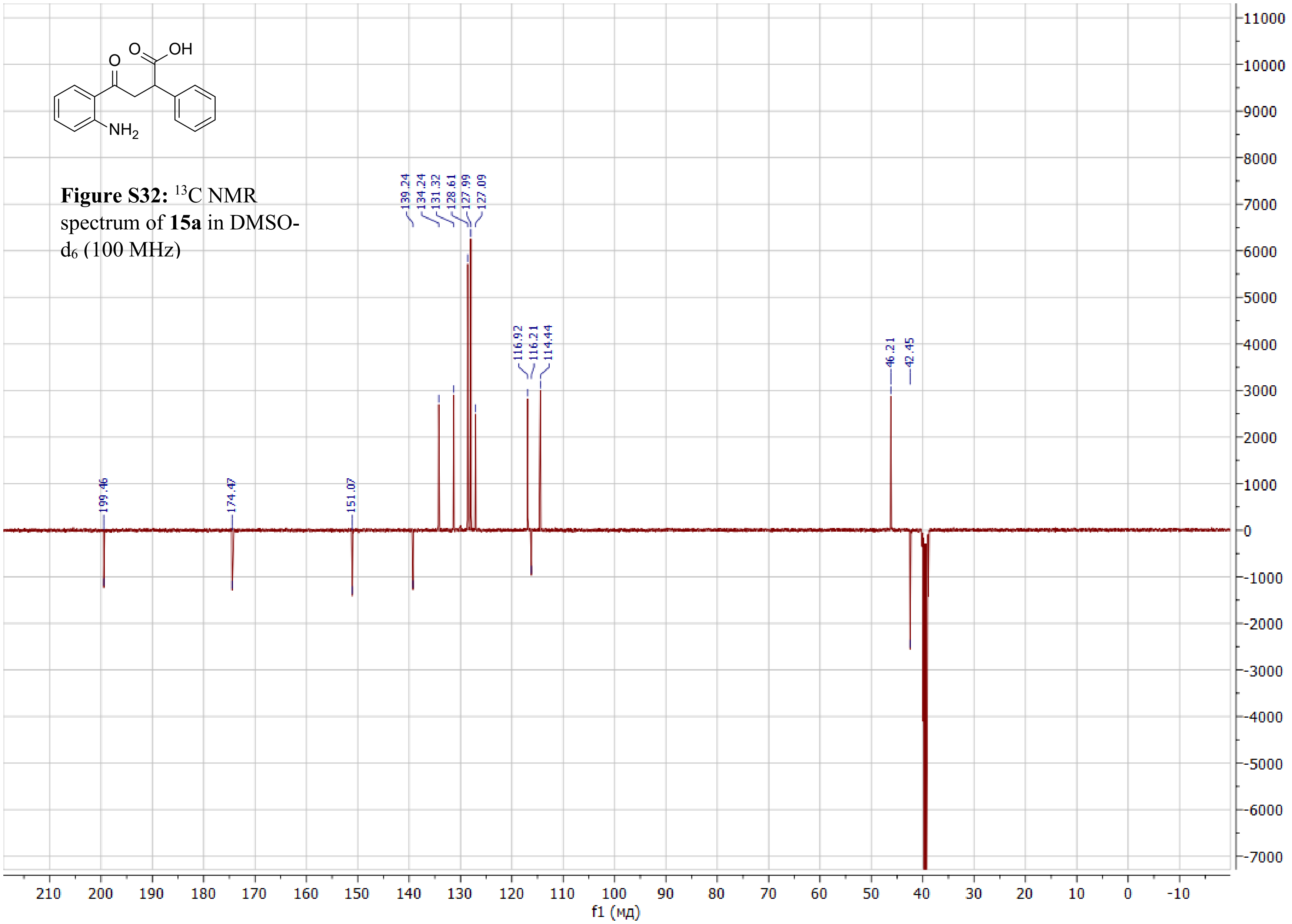

Figure S32: ^{13}C NMR spectrum of **15a** in DMSO-d_6 (100 MHz)

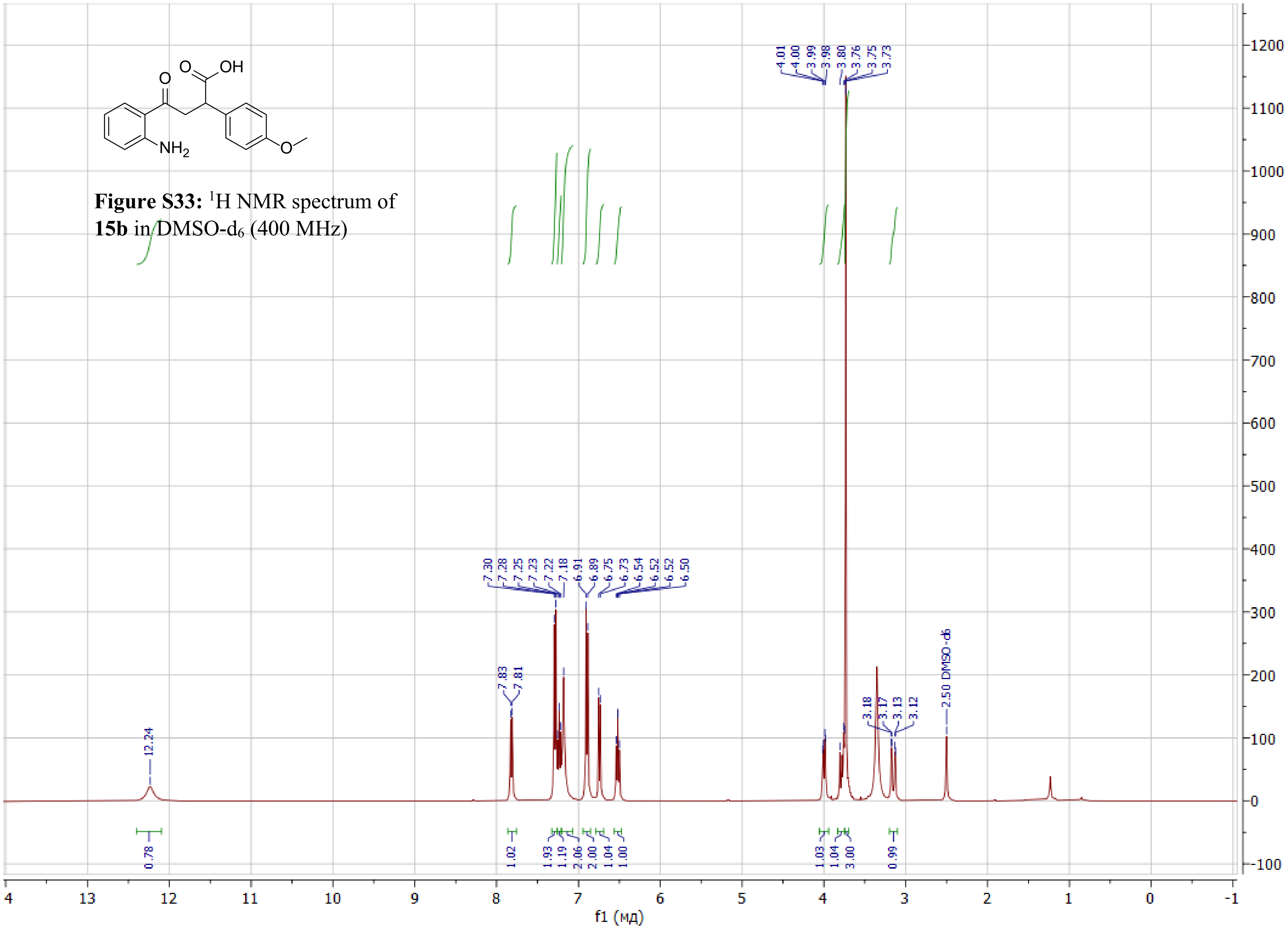

Figure S33: ^{1}H NMR spectrum of **15b** in DMSO-d_6 (400 MHz)

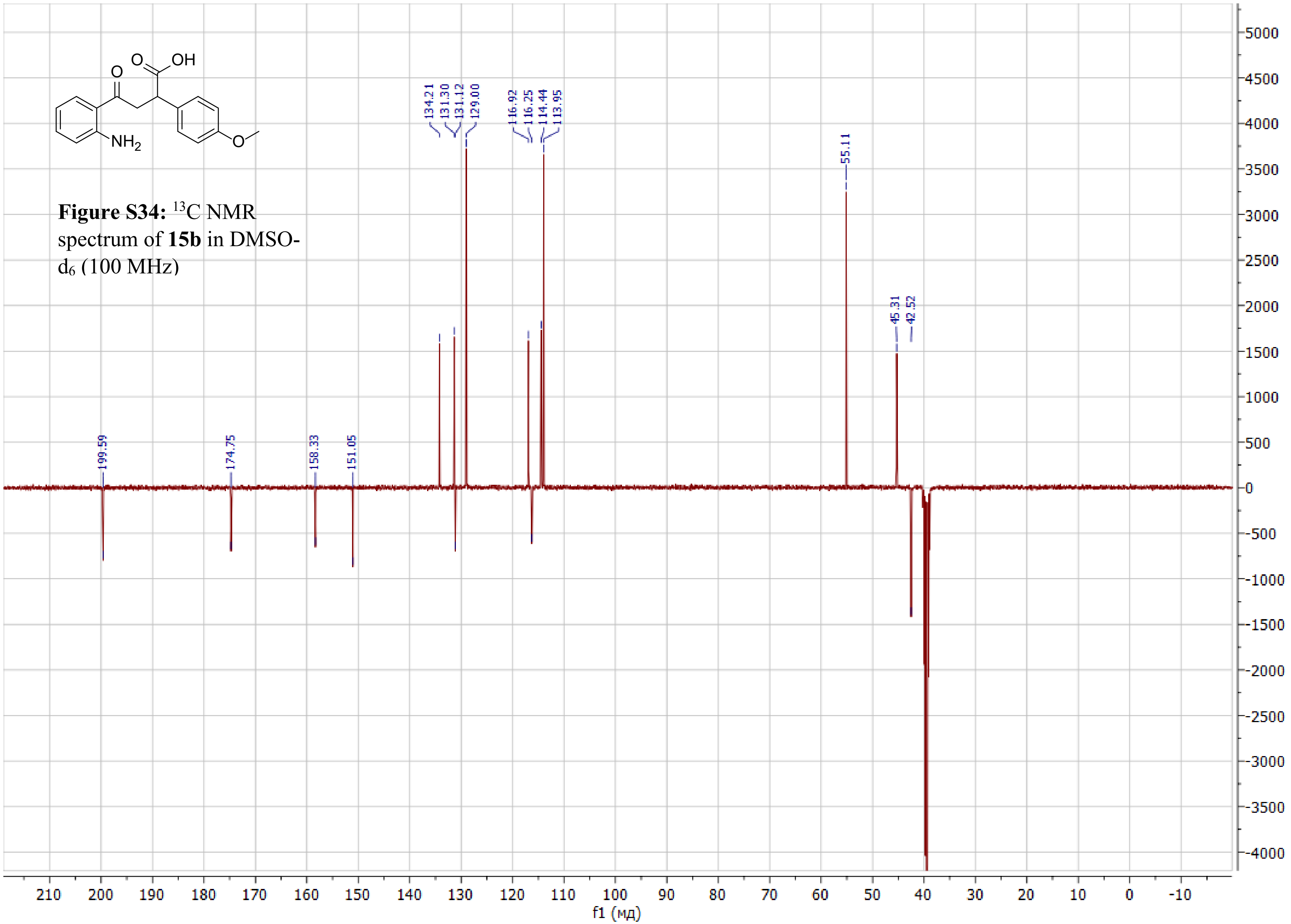

Figure S34: ^{13}C NMR spectrum of **15b** in DMSO-d_6 (100 MHz)

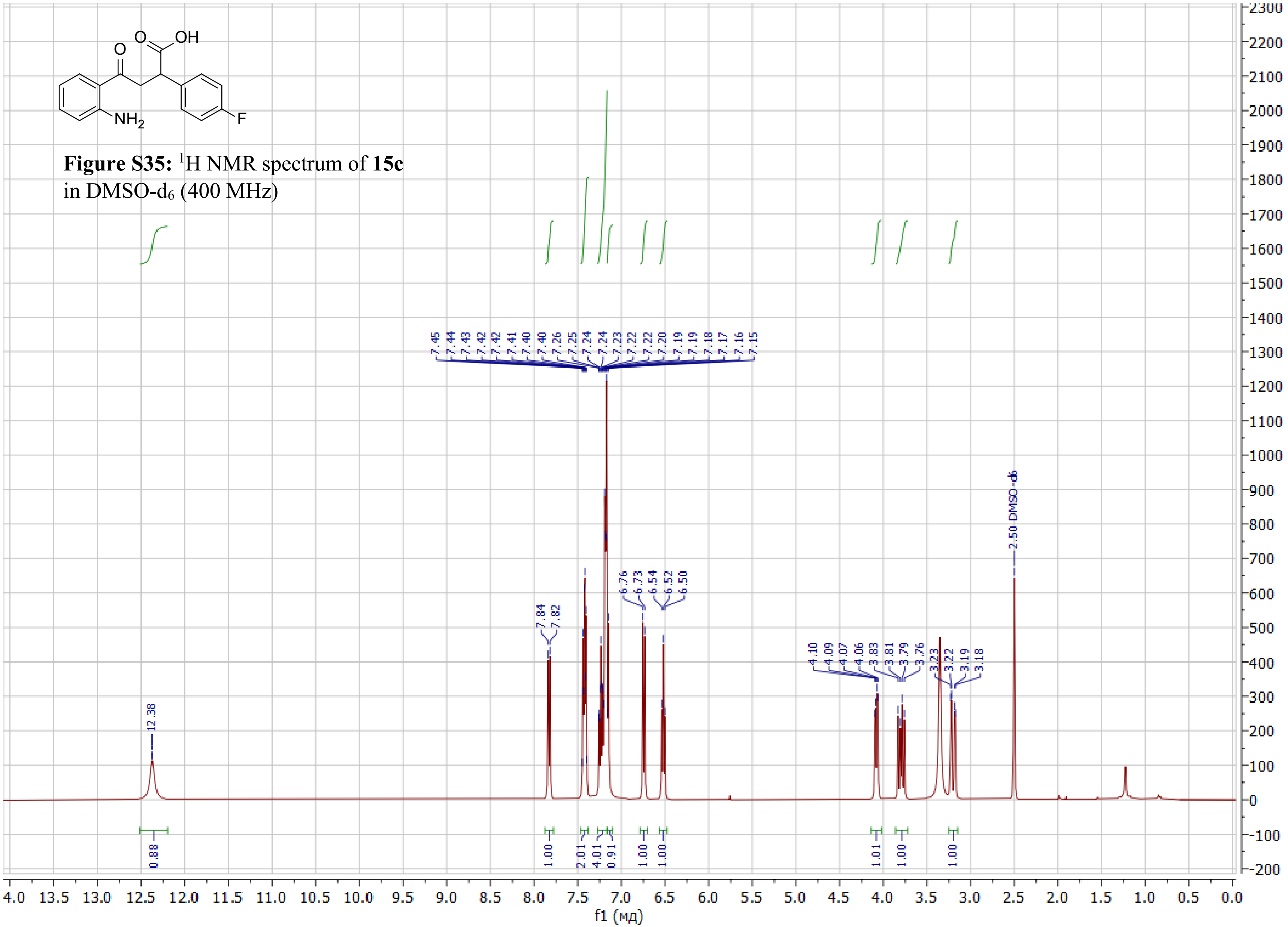

Figure S35: ^{1}H NMR spectrum of **15c** in DMSO-d_6 (400 MHz)

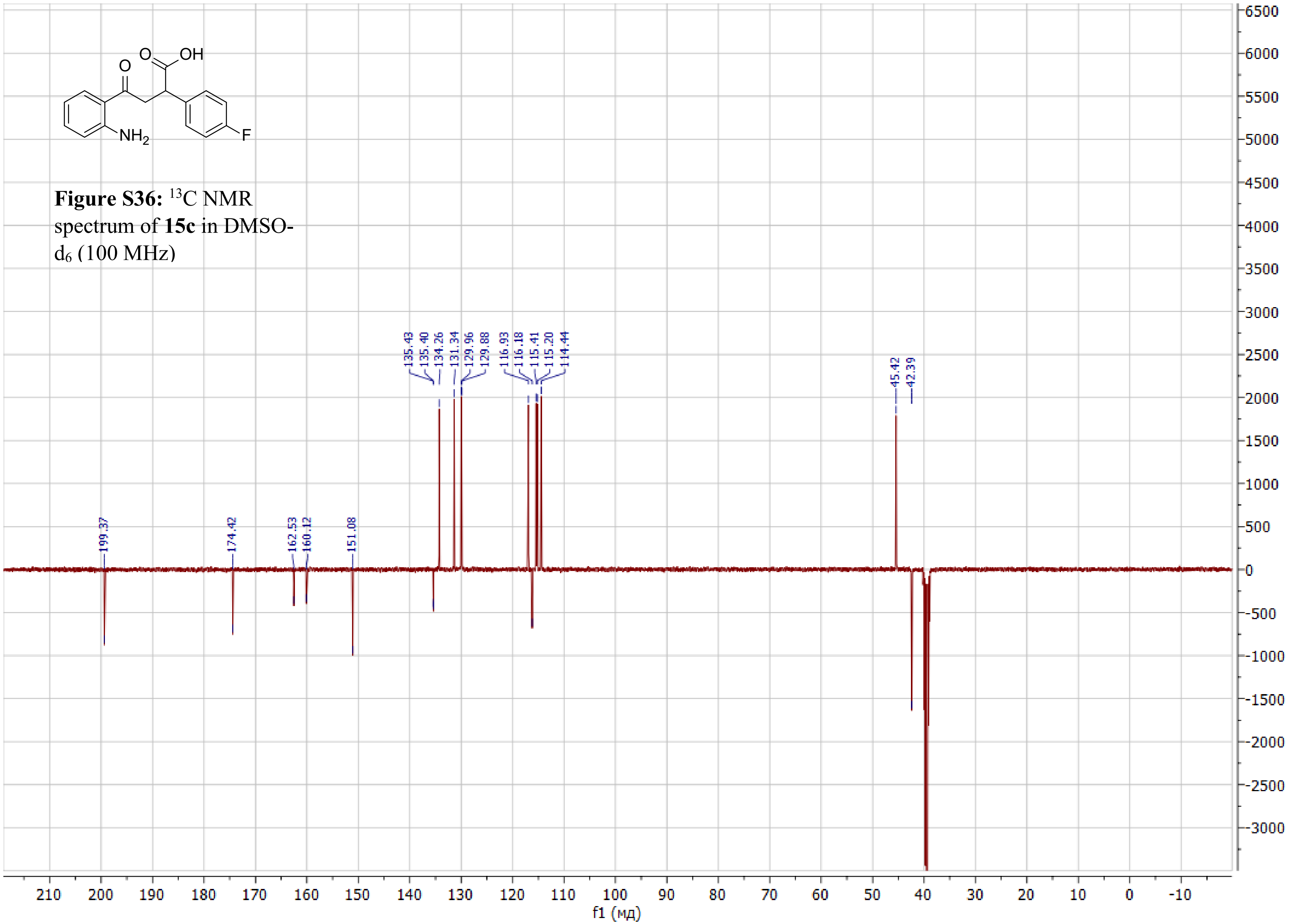

Figure S36: ^{13}C NMR spectrum of **15c** in DMSO-d$_6$ (100 MHz)

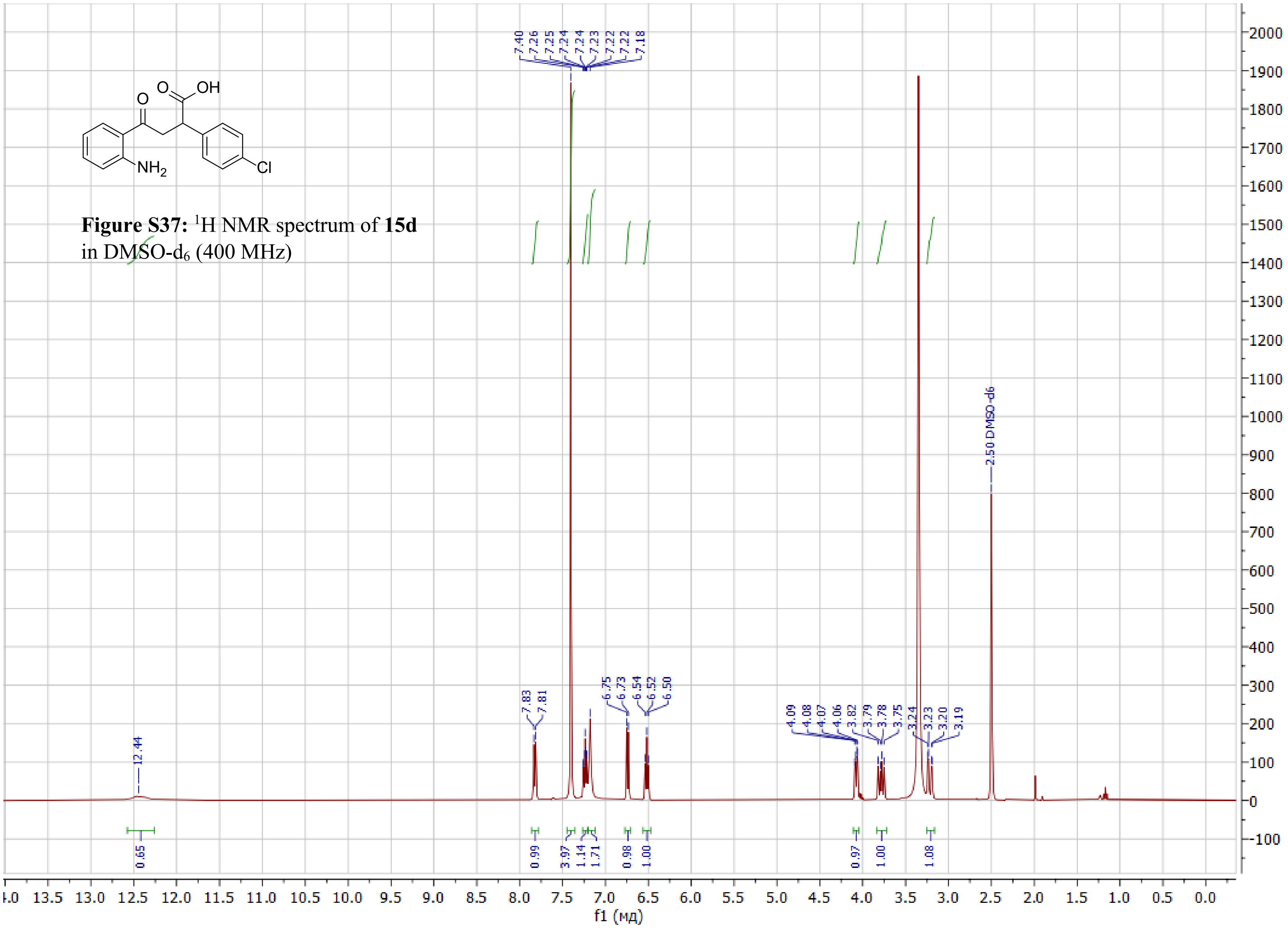

Figure S37: ^{1}H NMR spectrum of **15d** in DMSO-d_6 (400 MHz)

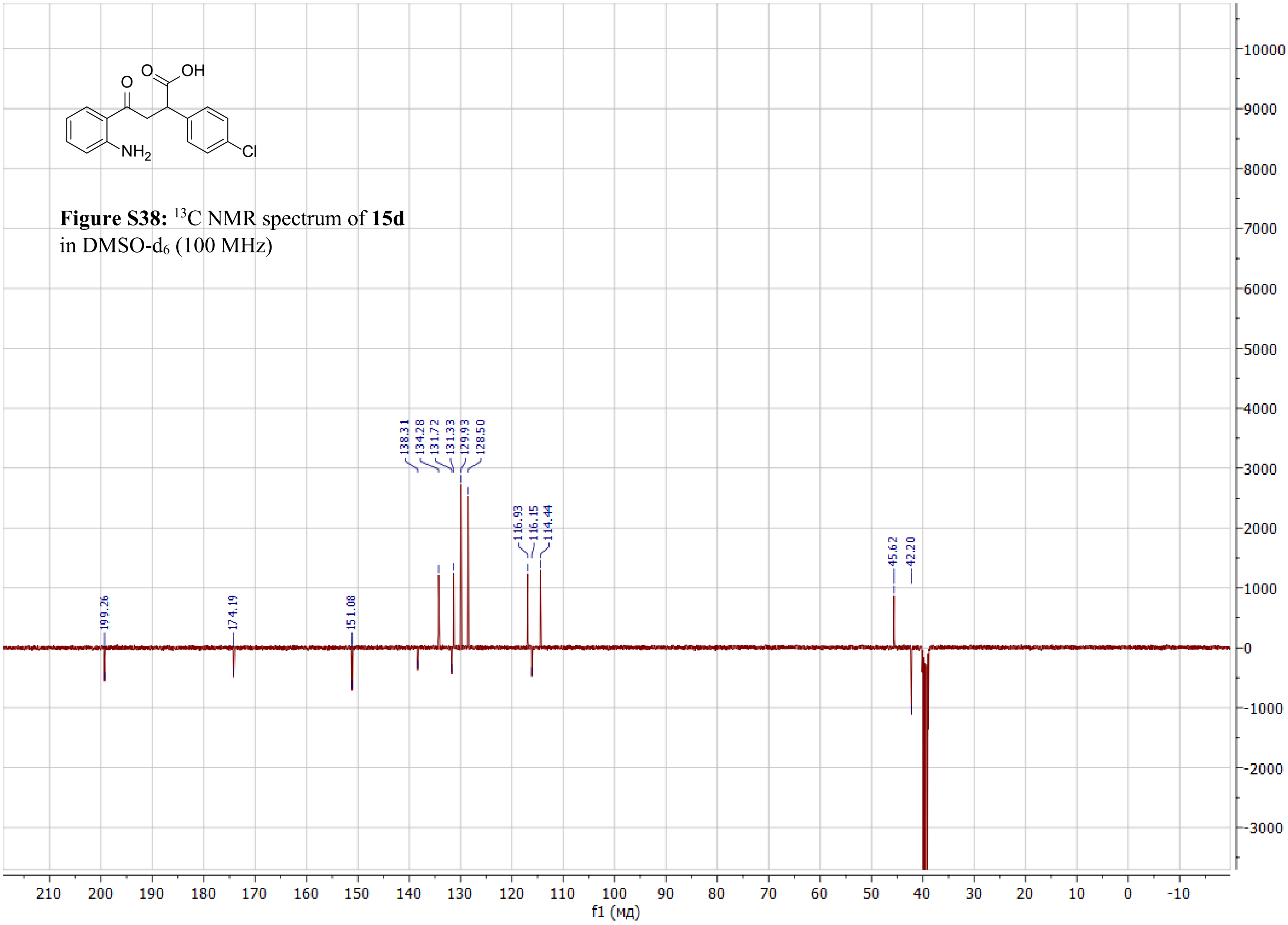

Figure S38: ^{13}C NMR spectrum of **15d** in DMSO-d_6 (100 MHz)

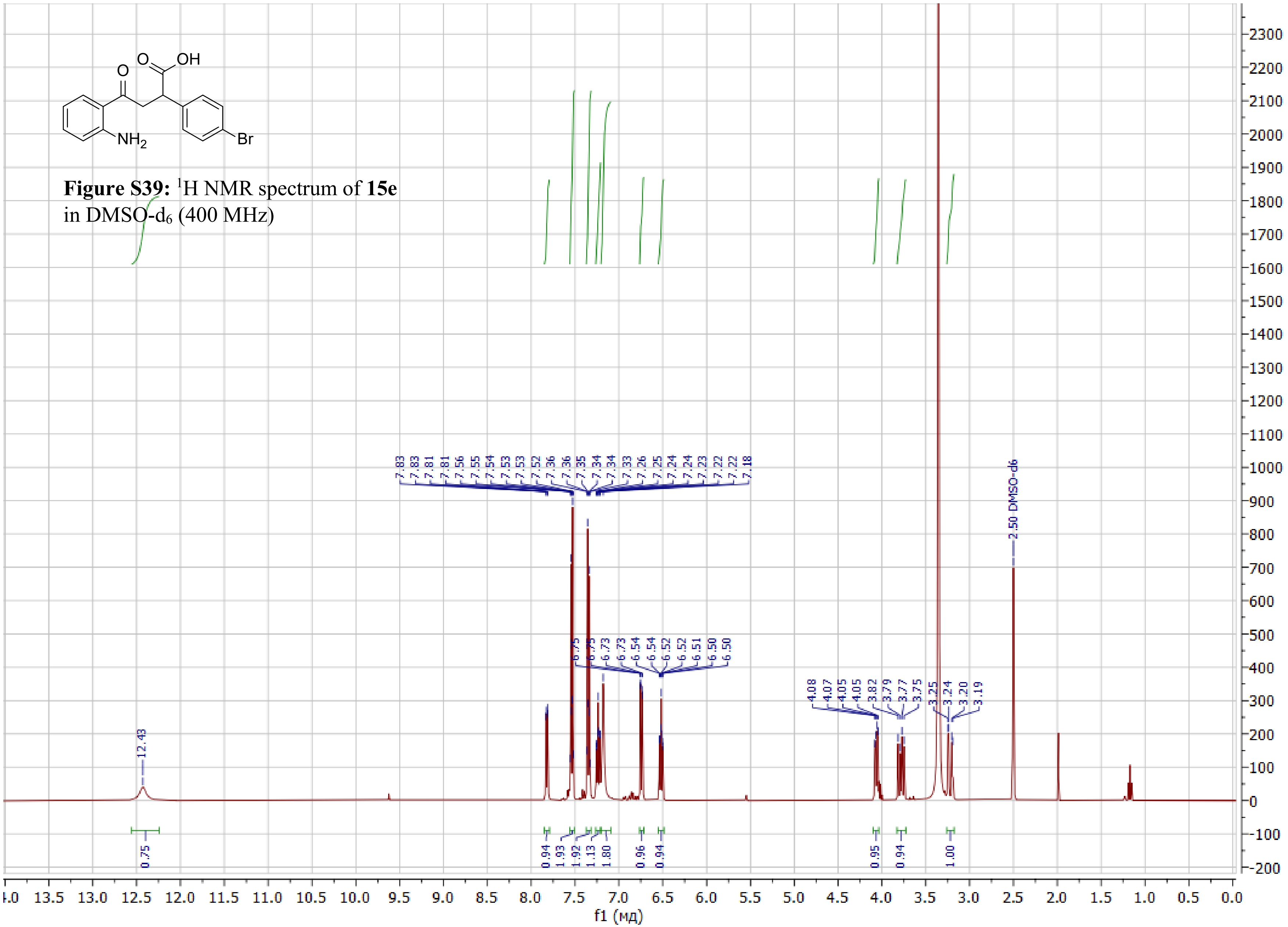

Figure S39: ^{1}H NMR spectrum of **15e** in DMSO-d$_6$ (400 MHz)

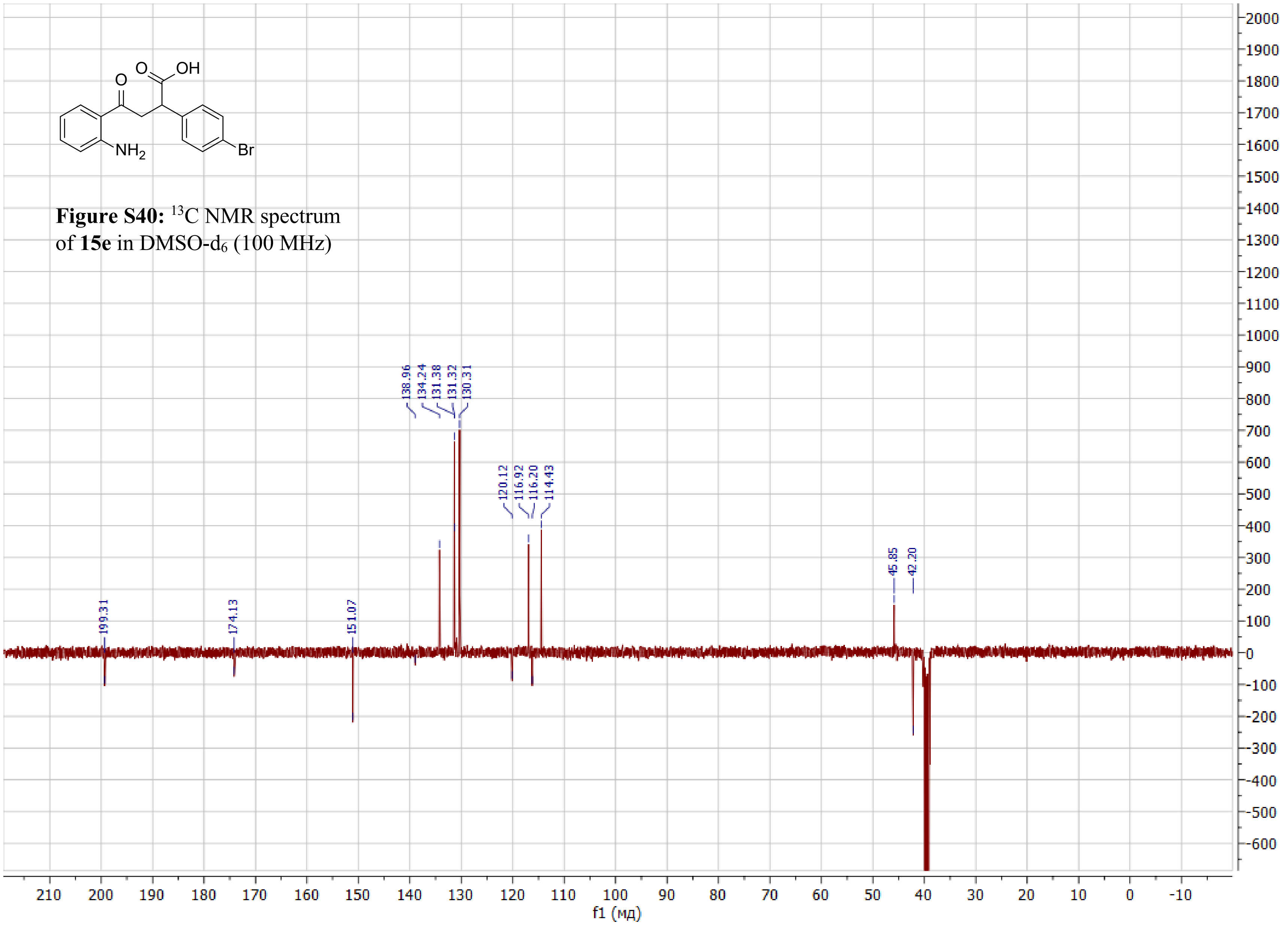

Figure S40: ^{13}C NMR spectrum of **15e** in DMSO-d_6 (100 MHz)

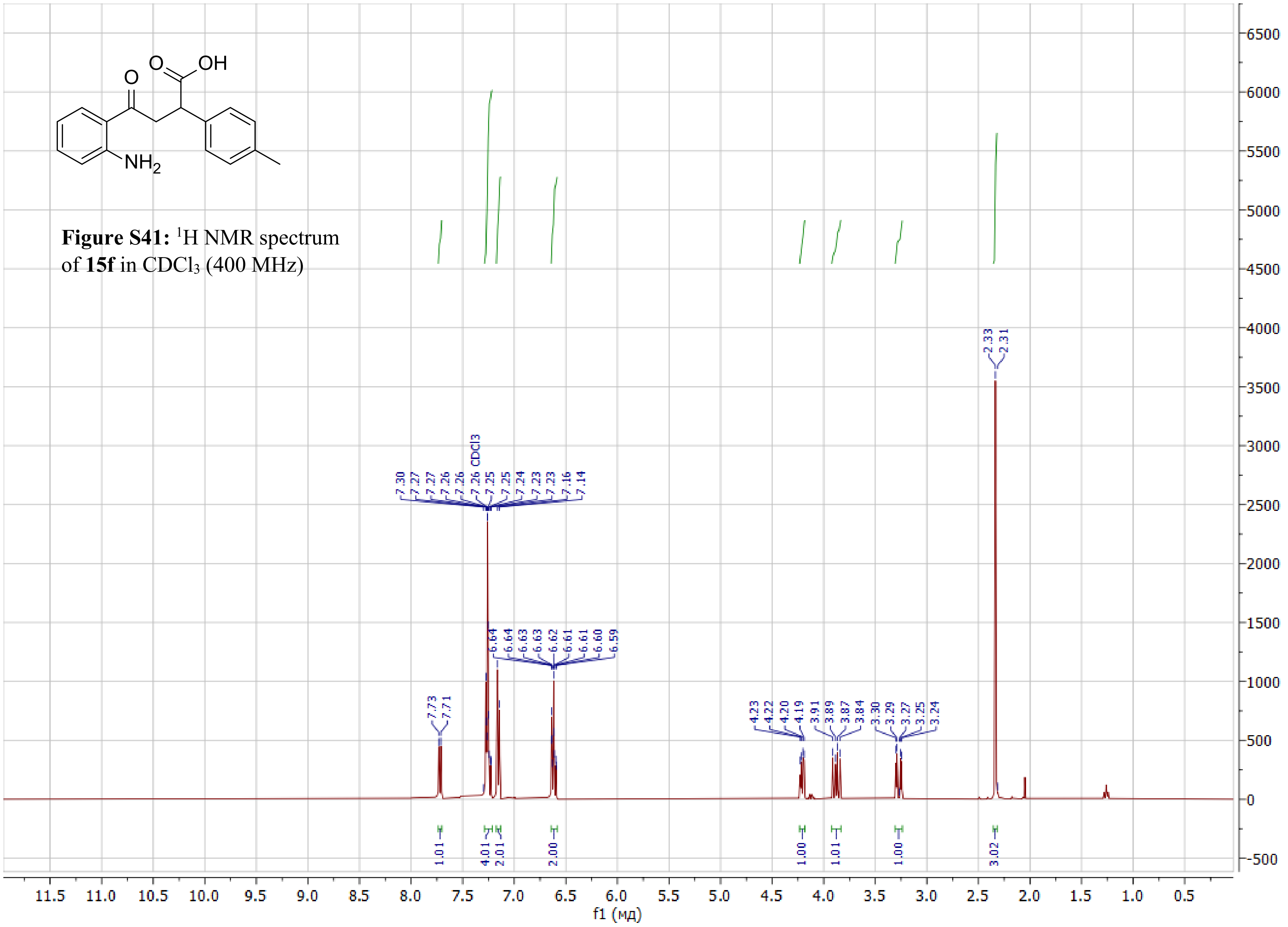

Figure S41: ^{1}H NMR spectrum of **15f** in $CDCl_3$ (400 MHz)

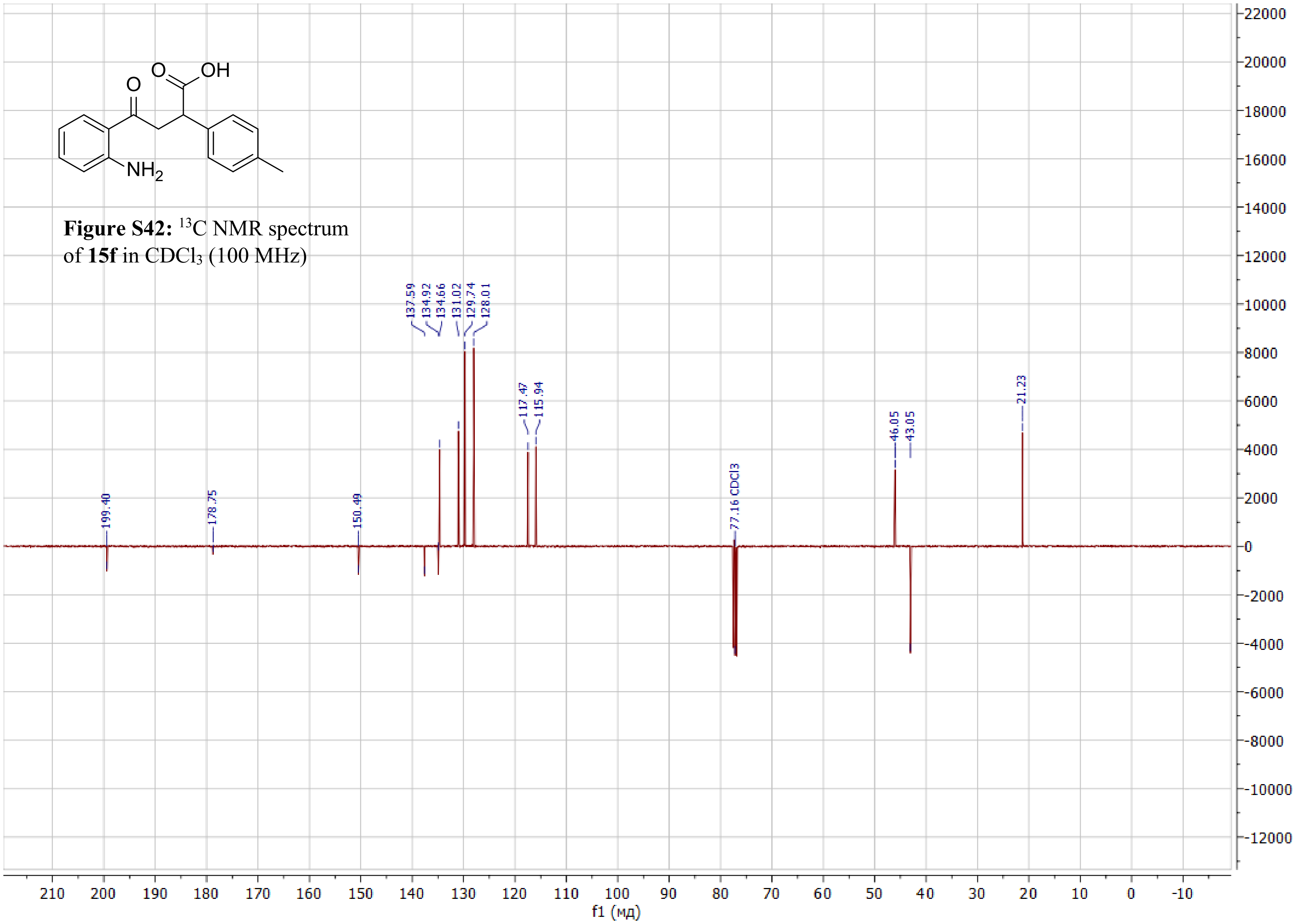

Figure S42: ^{13}C NMR spectrum of **15f** in $CDCl_3$ (100 MHz)

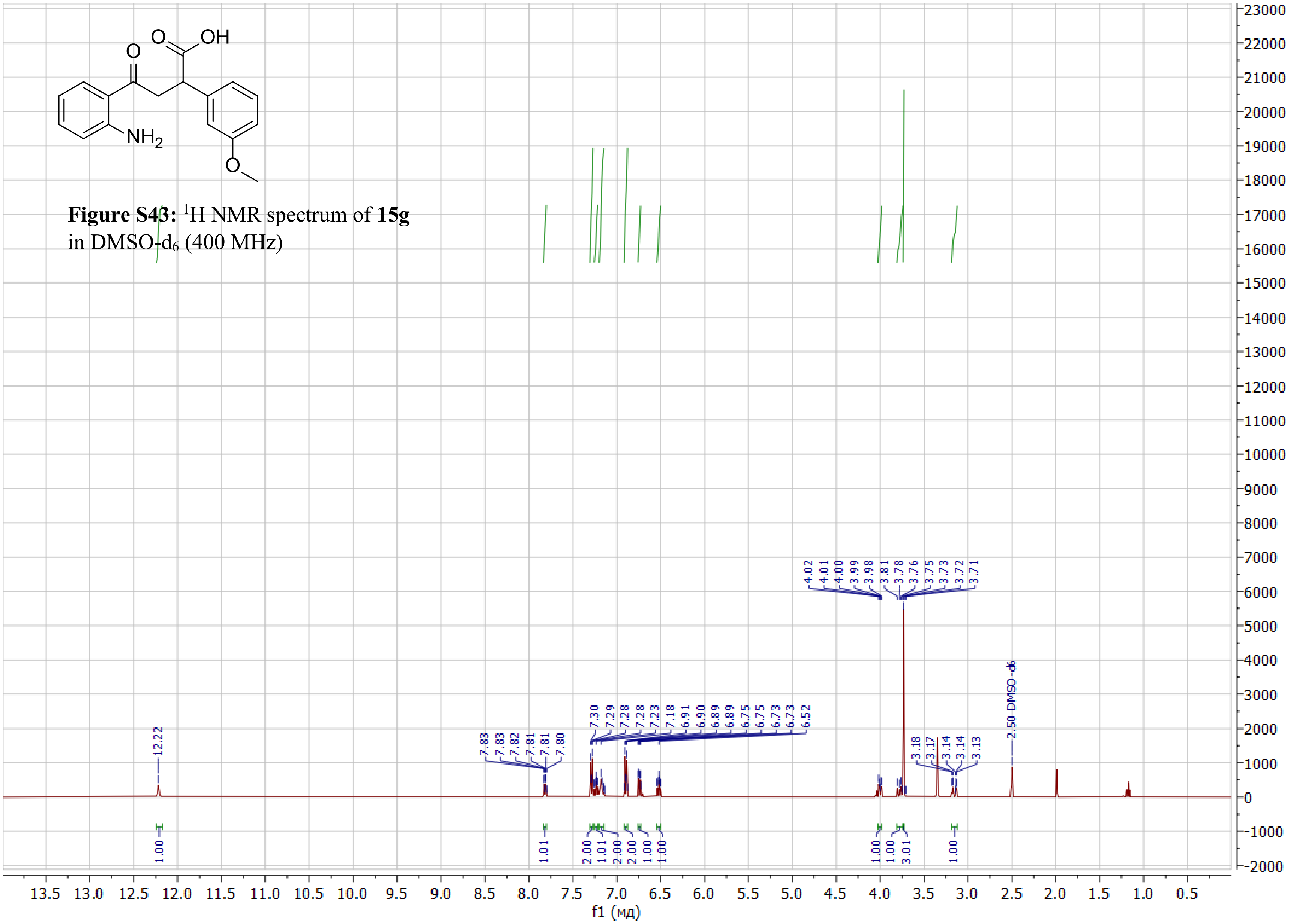

Figure S43: ^{1}H NMR spectrum of **15g** in DMSO-d$_6$ (400 MHz)

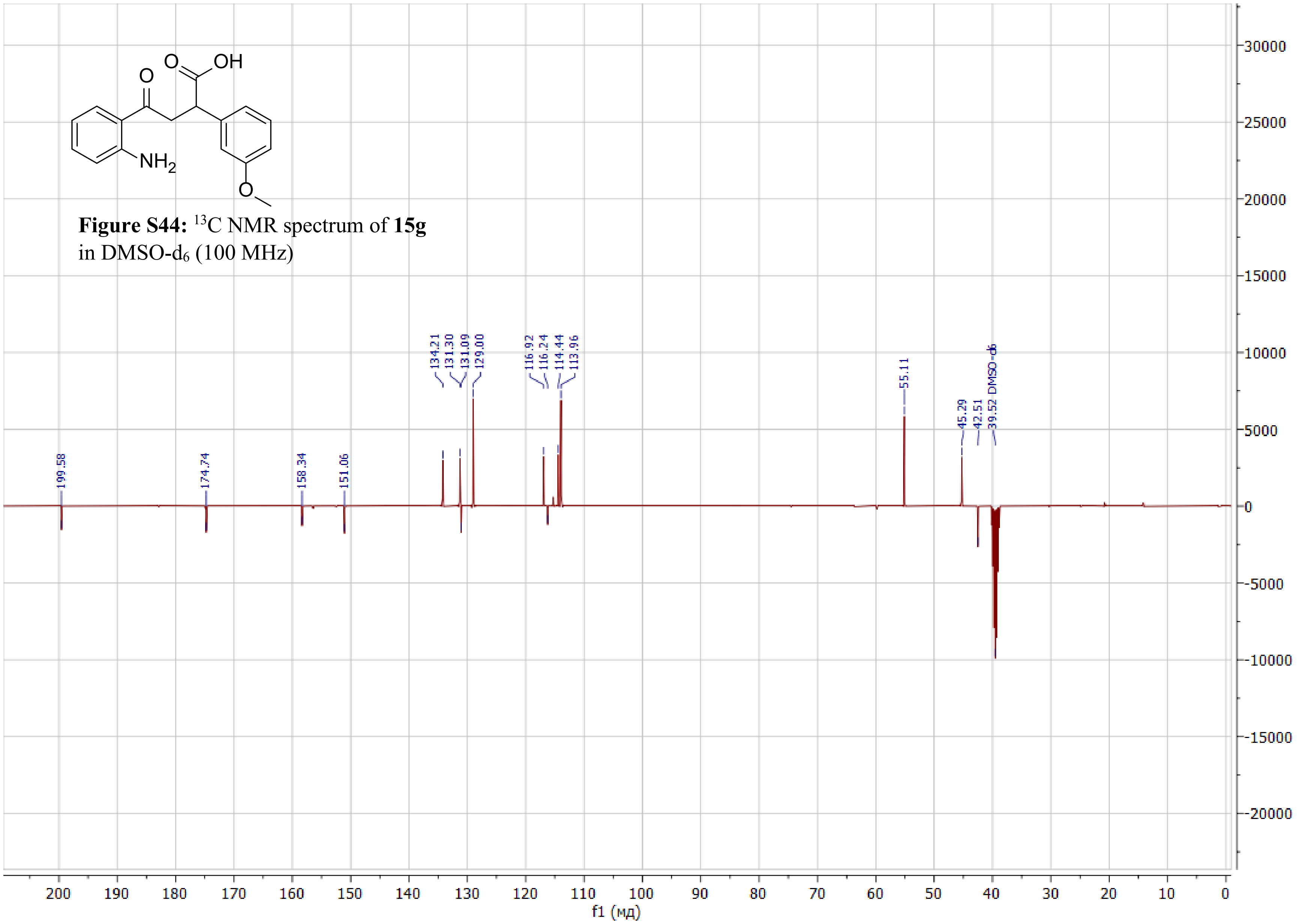

Figure S44: ^{13}C NMR spectrum of **15g** in DMSO-d_6 (100 MHz)

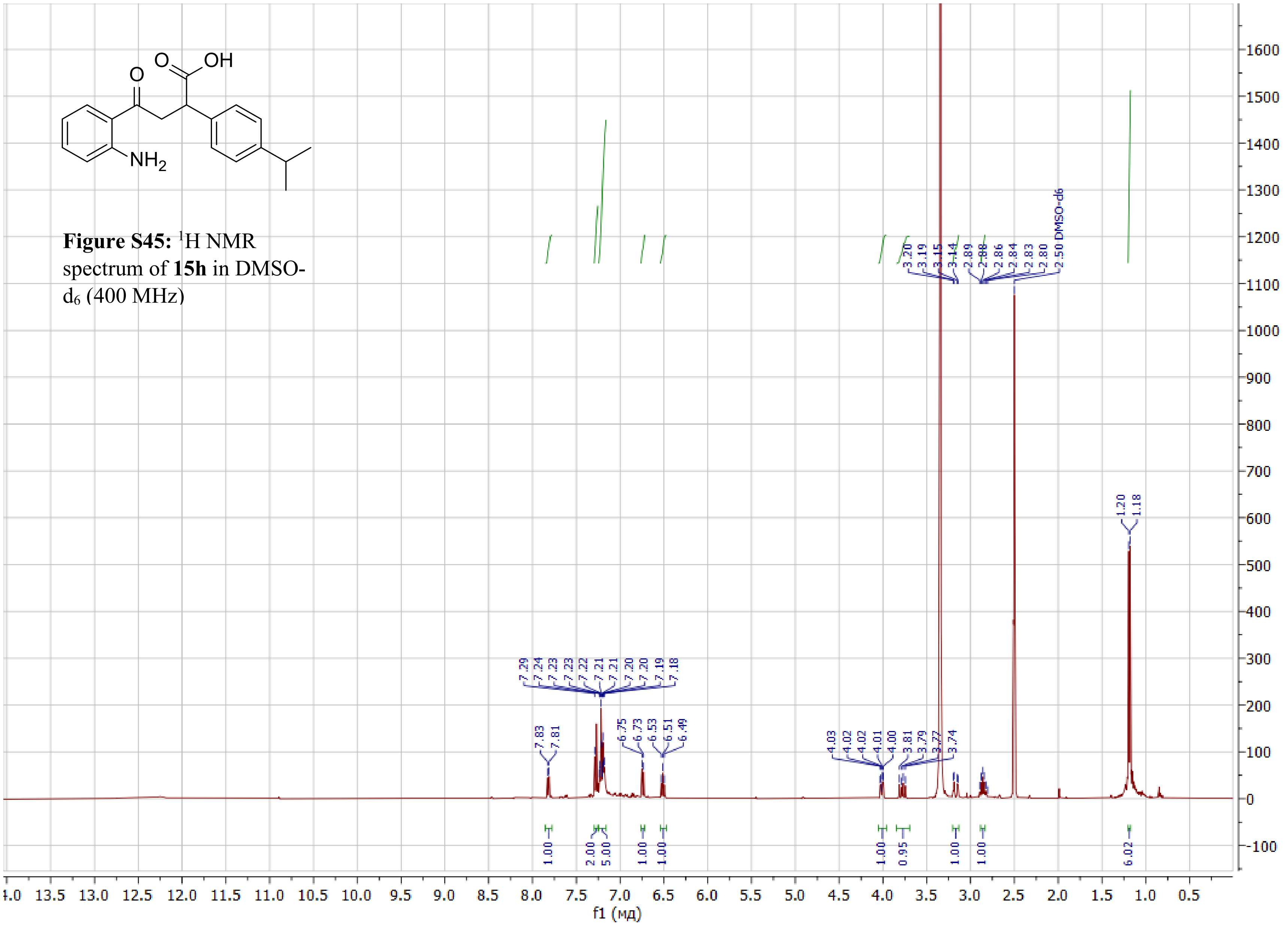

Figure S45: ^{1}H NMR spectrum of **15h** in DMSO-d_6 (400 MHz)

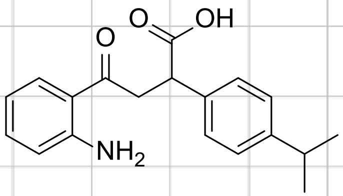

Figure S46: ^{13}C NMR spectrum of **15h** in DMSO-d_6 (100 MHz)

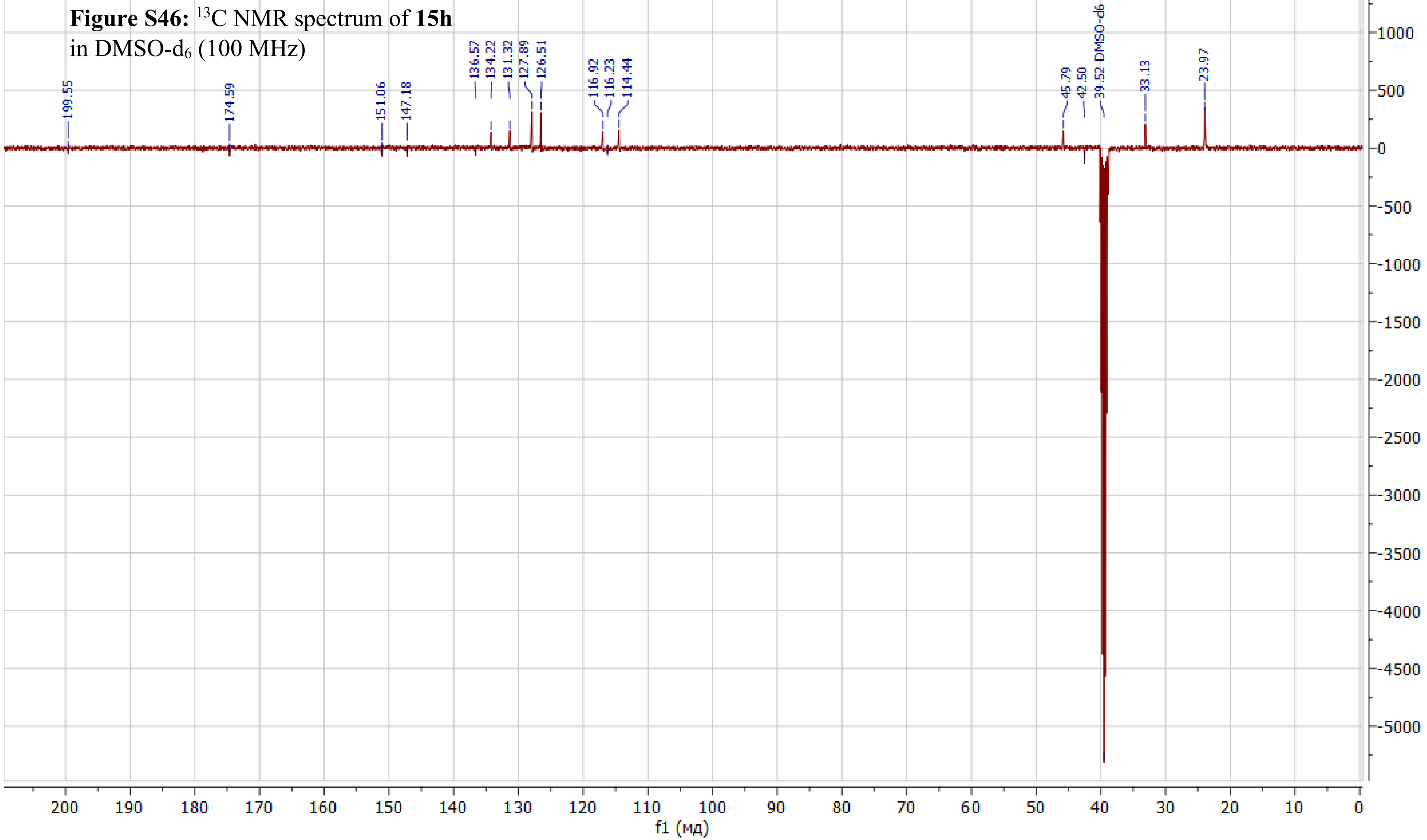

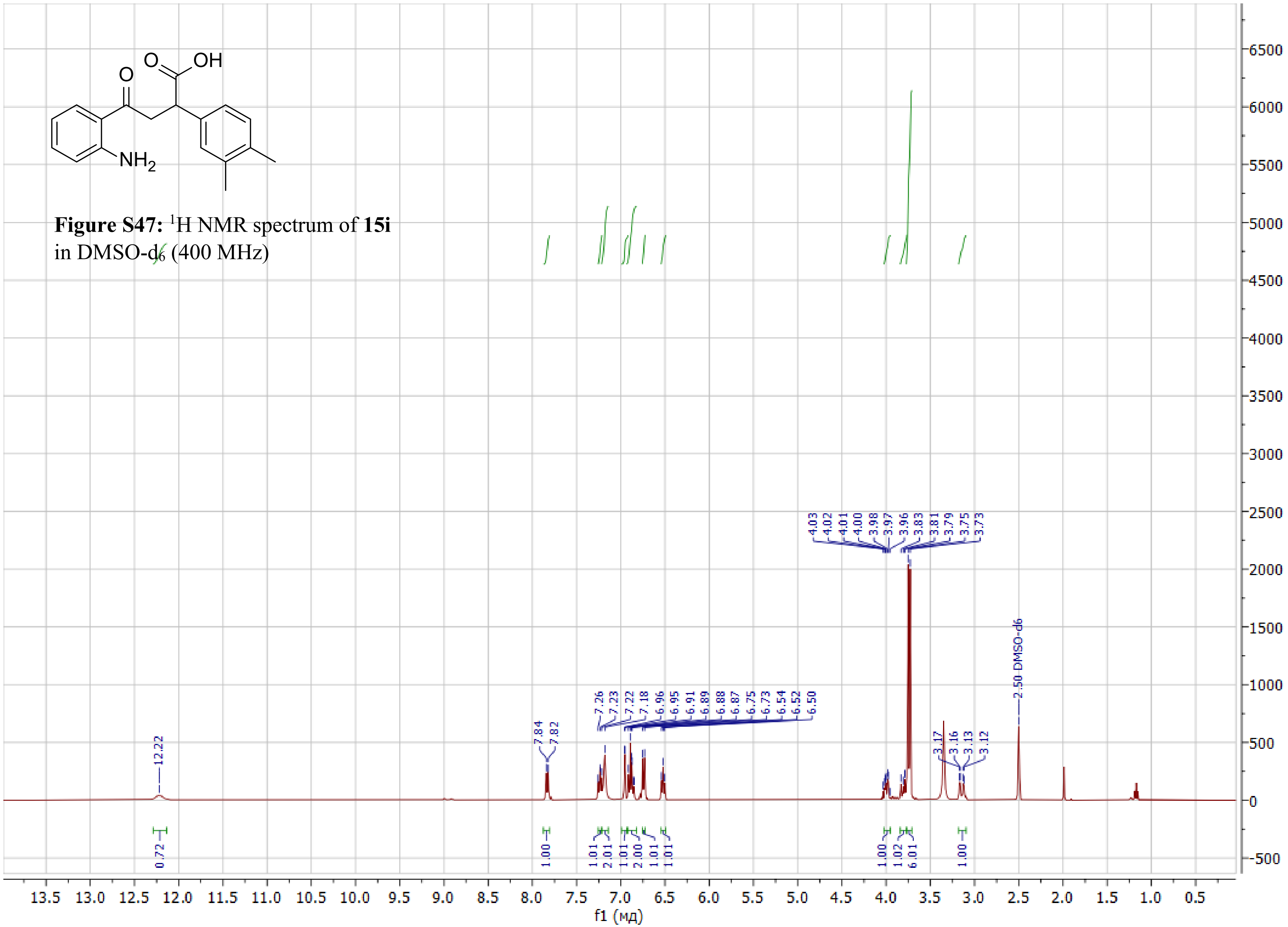

Figure S47: ^{1}H NMR spectrum of **15i** in DMSO-d_6 (400 MHz)

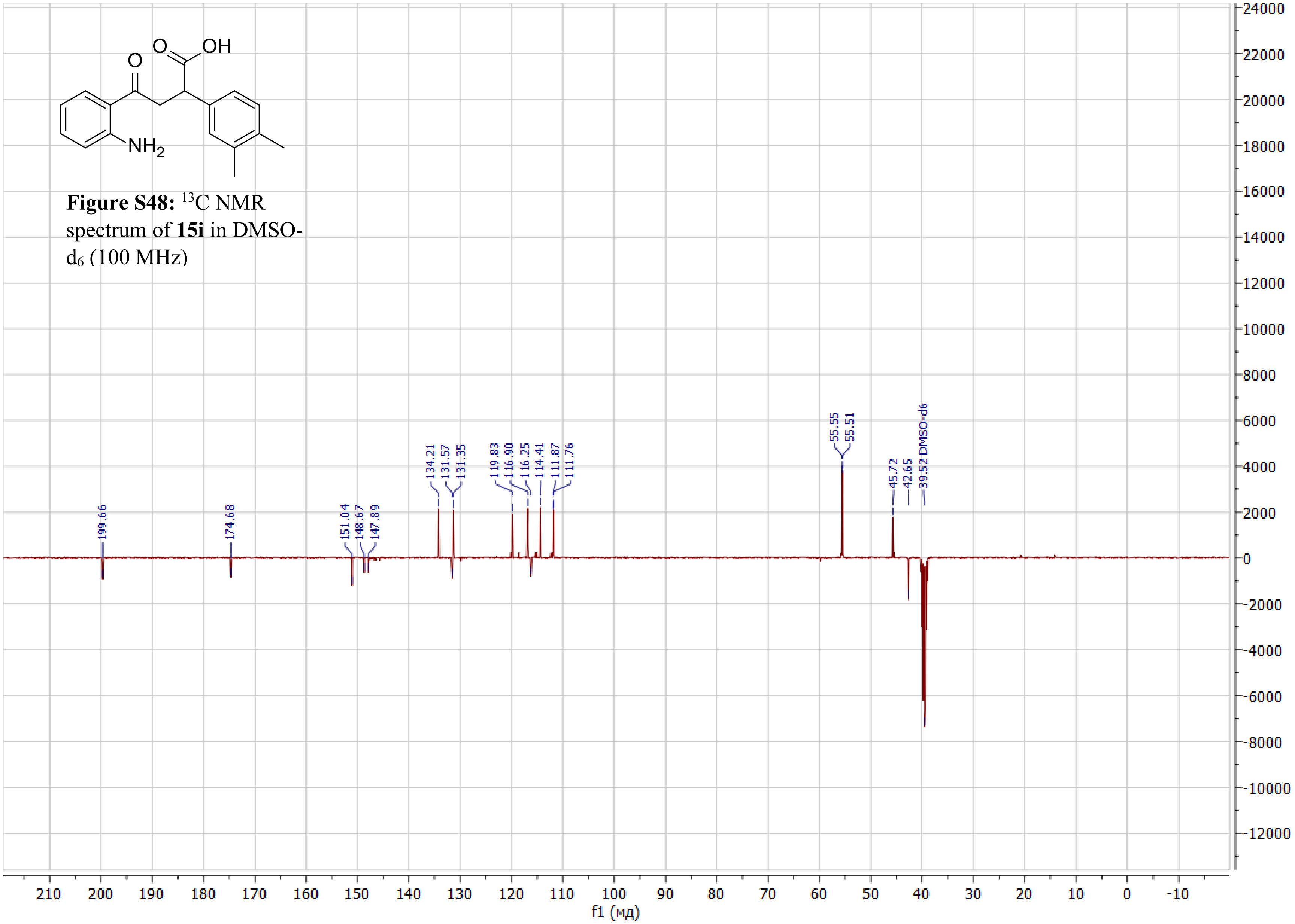

Figure S48: ^{13}C NMR spectrum of **15i** in DMSO-d_6 (100 MHz)

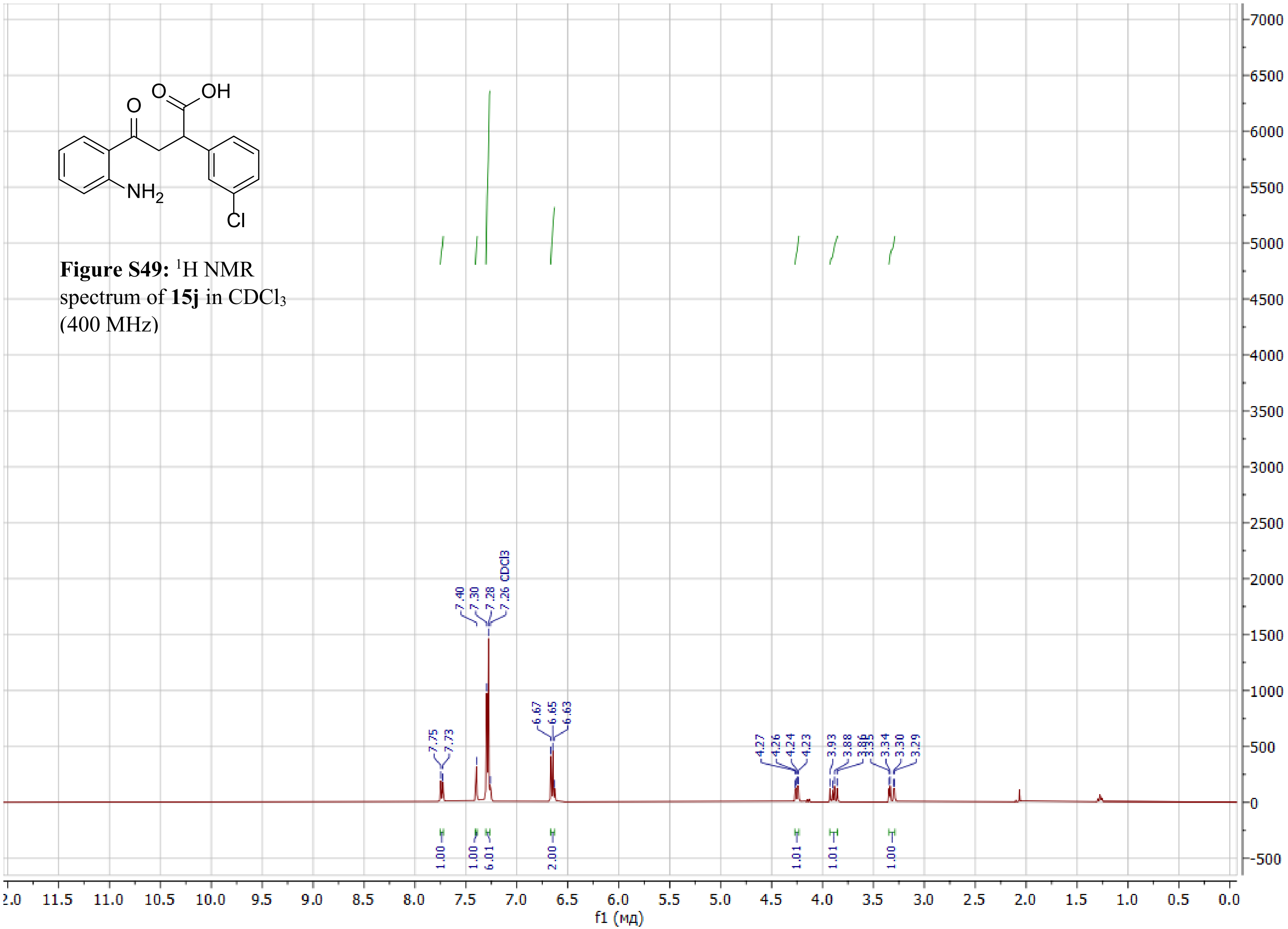

Figure S49: ^{1}H NMR spectrum of **15j** in $CDCl_3$ (400 MHz)

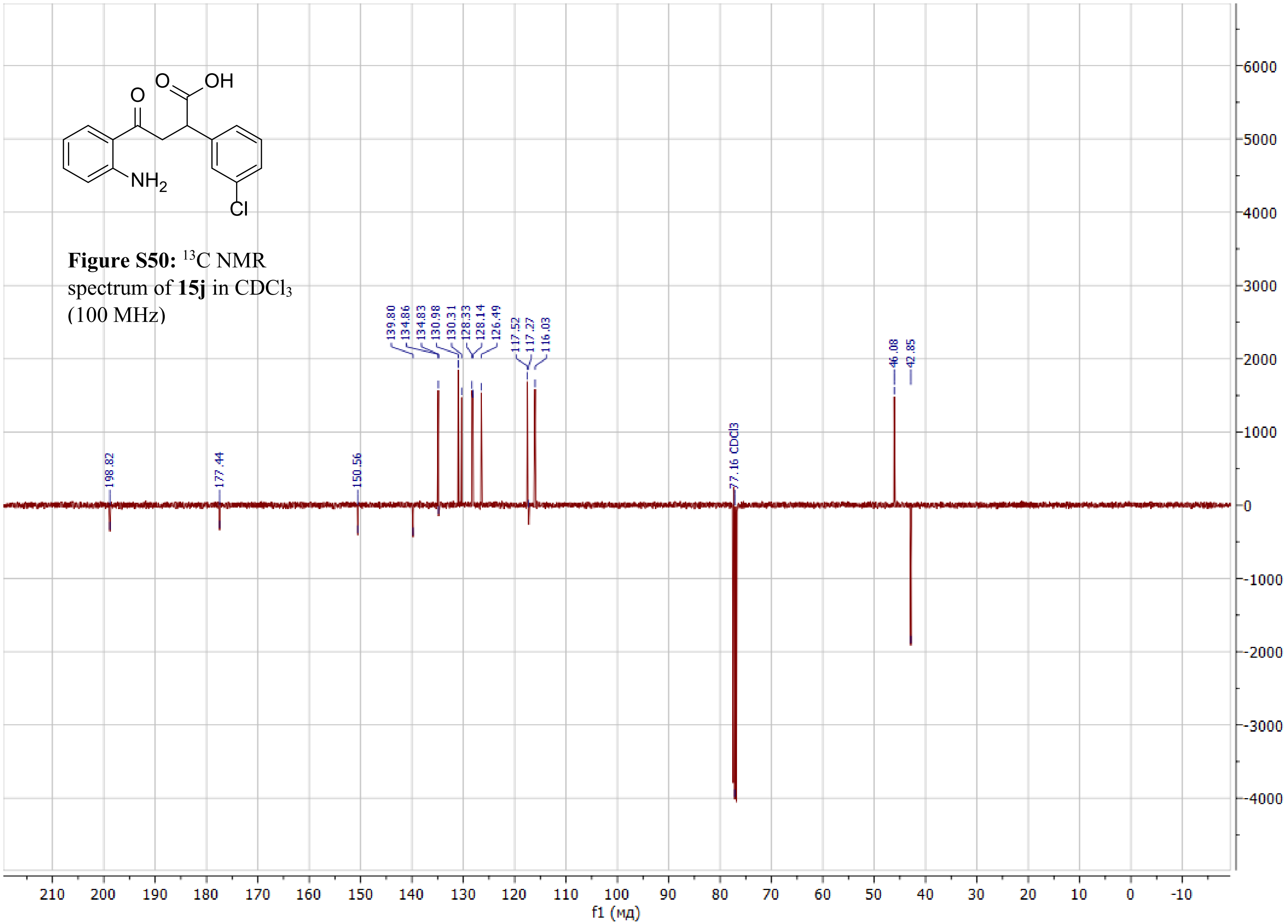

Figure S50: ^{13}C NMR spectrum of **15j** in $CDCl_3$ (100 MHz)

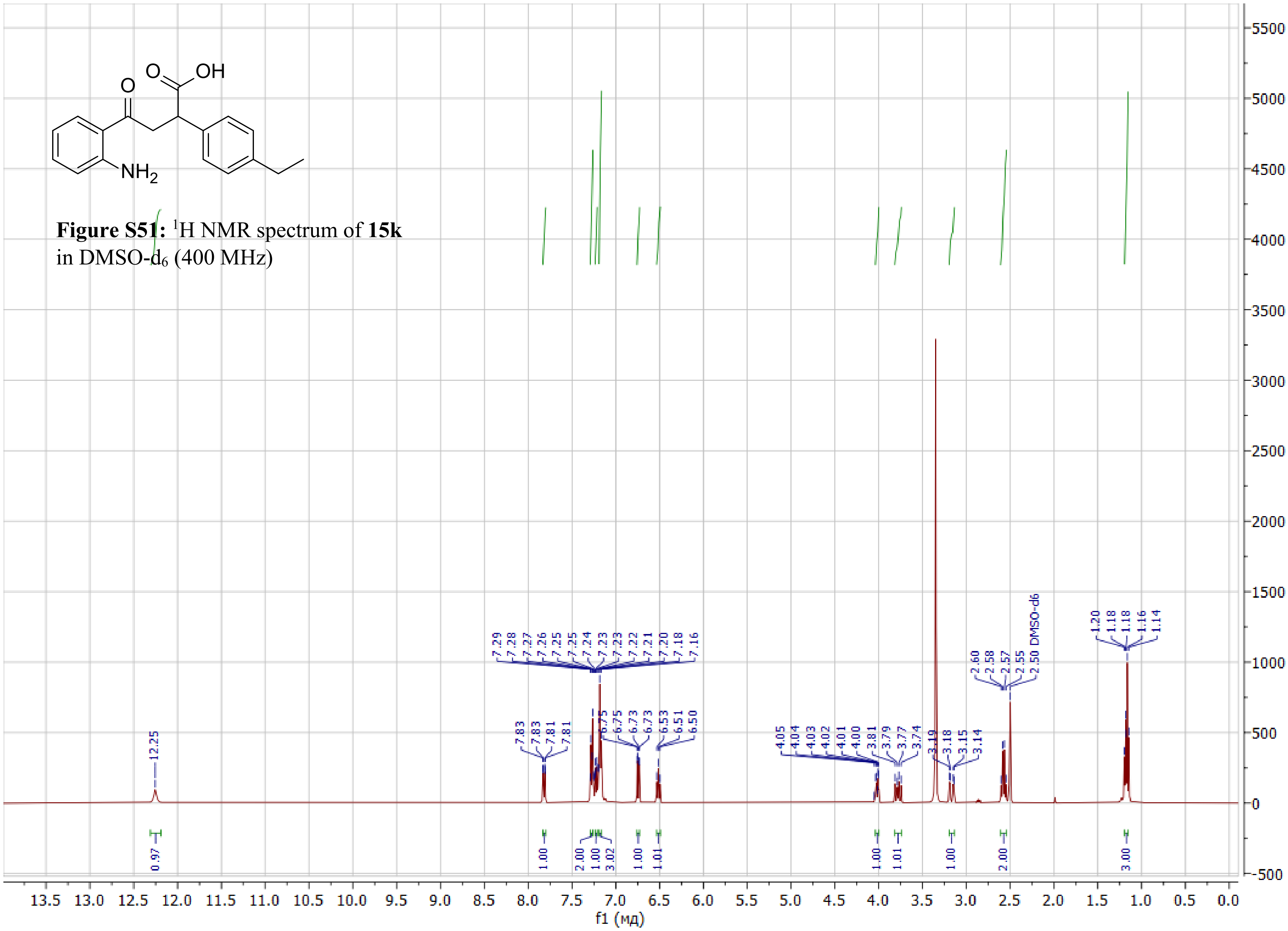

Figure S51: ^{1}H NMR spectrum of **15k** in DMSO-d_6 (400 MHz)

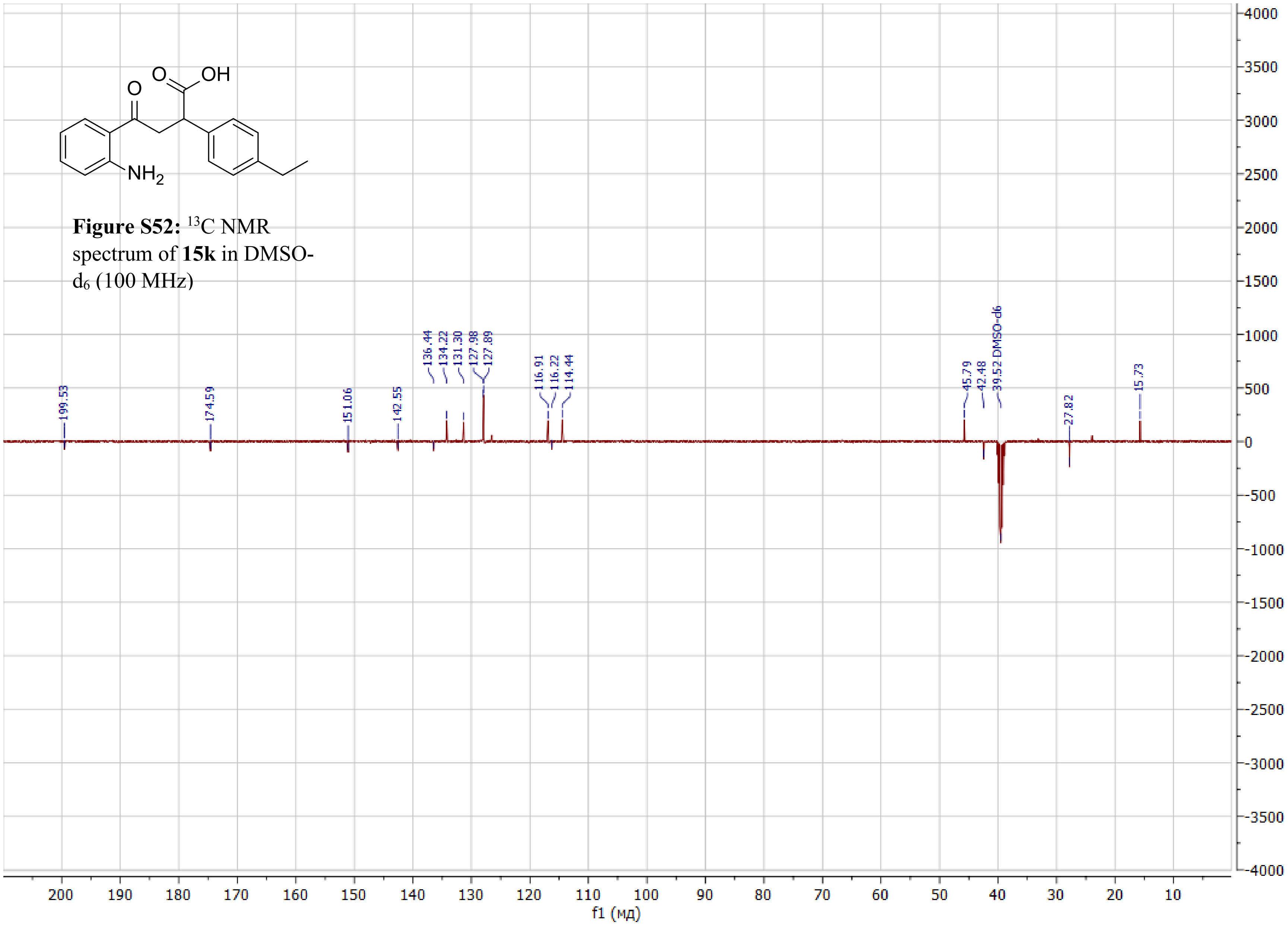

Figure S52: ^{13}C NMR spectrum of **15k** in DMSO-d_6 (100 MHz)

^{1}H and ^{13}C NMR spectral charts for 3-aryl-3,4-dihydro-1H-benzo[b]azepine-2,5-diones **16a-k**

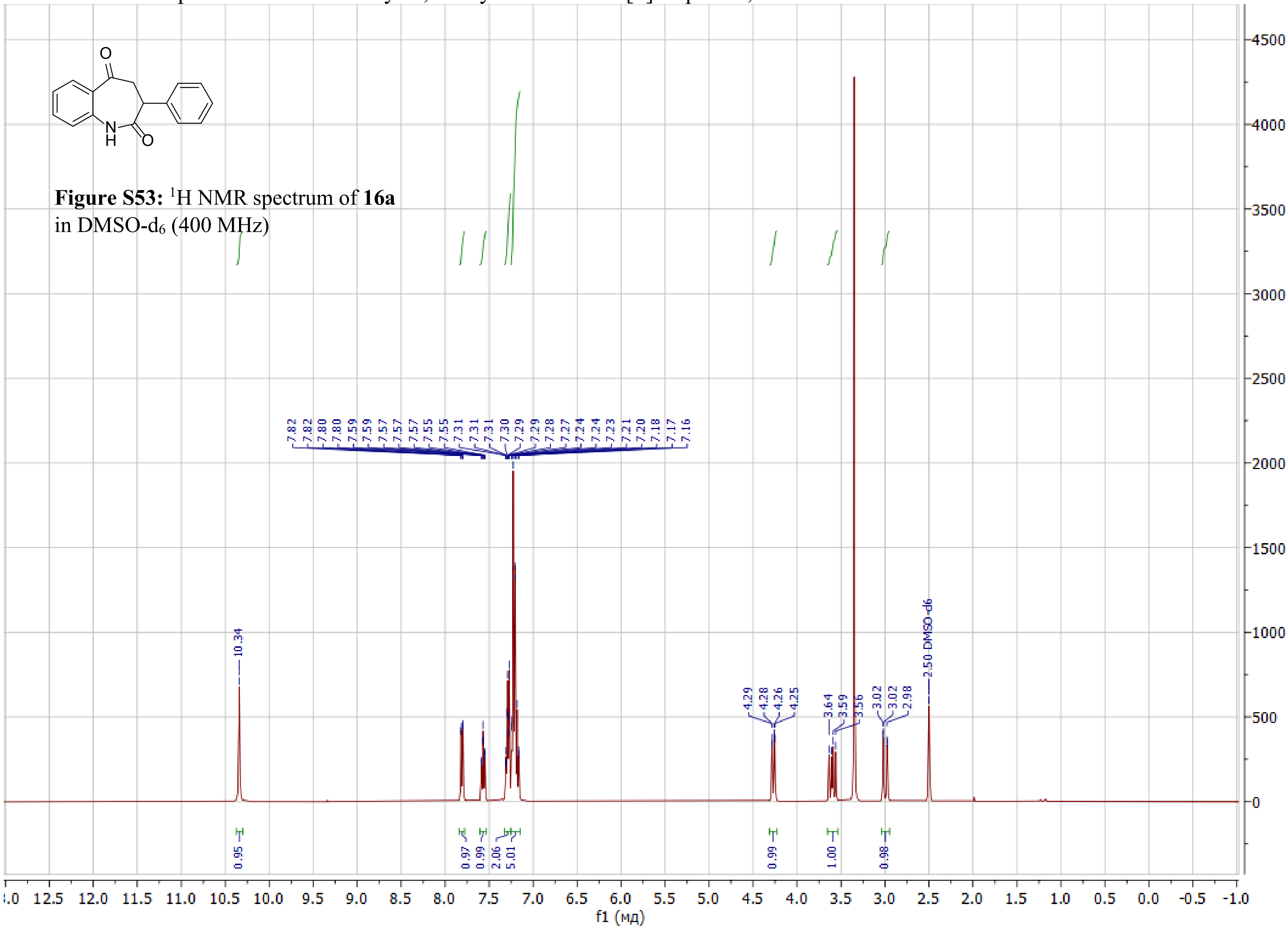

Figure S53: ^{1}H NMR spectrum of **16a** in DMSO-d_6 (400 MHz)

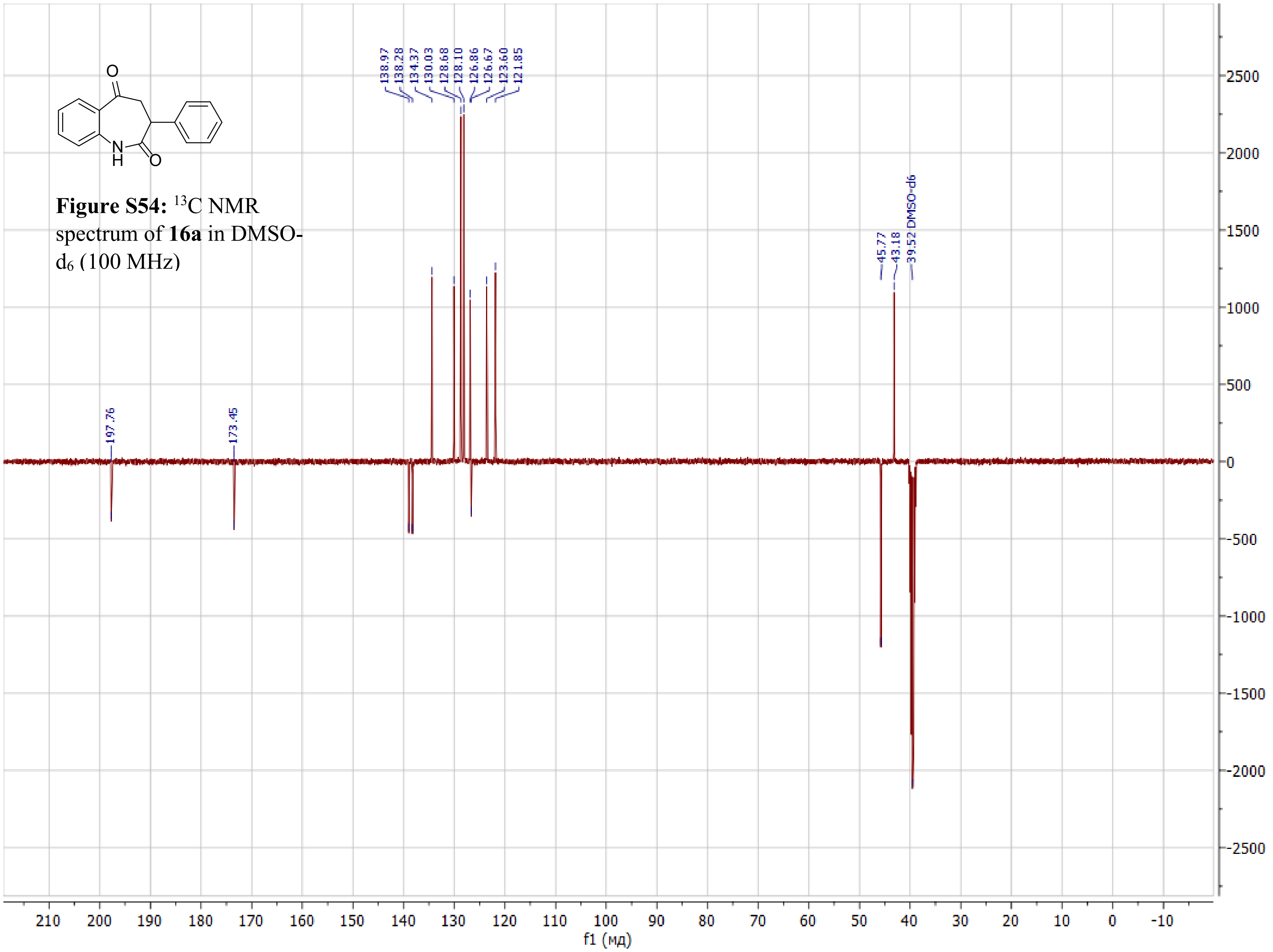

Figure S54: ^{13}C NMR spectrum of **16a** in DMSO-d$_6$ (100 MHz)

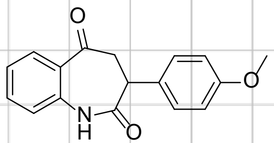

Figure S55: ^{1}H NMR spectrum of **16b** in $CDCl_3$ (400 MHz)

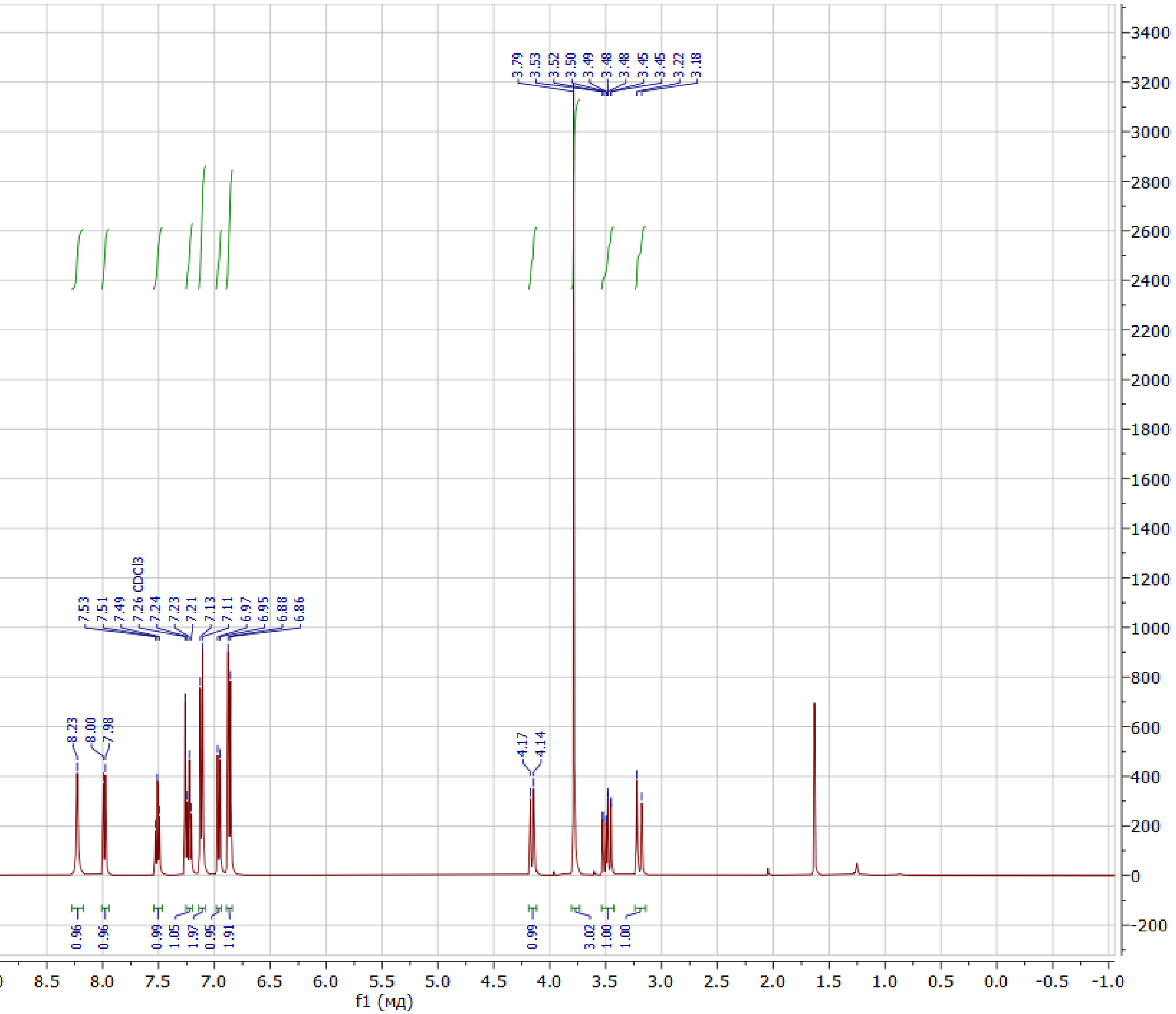

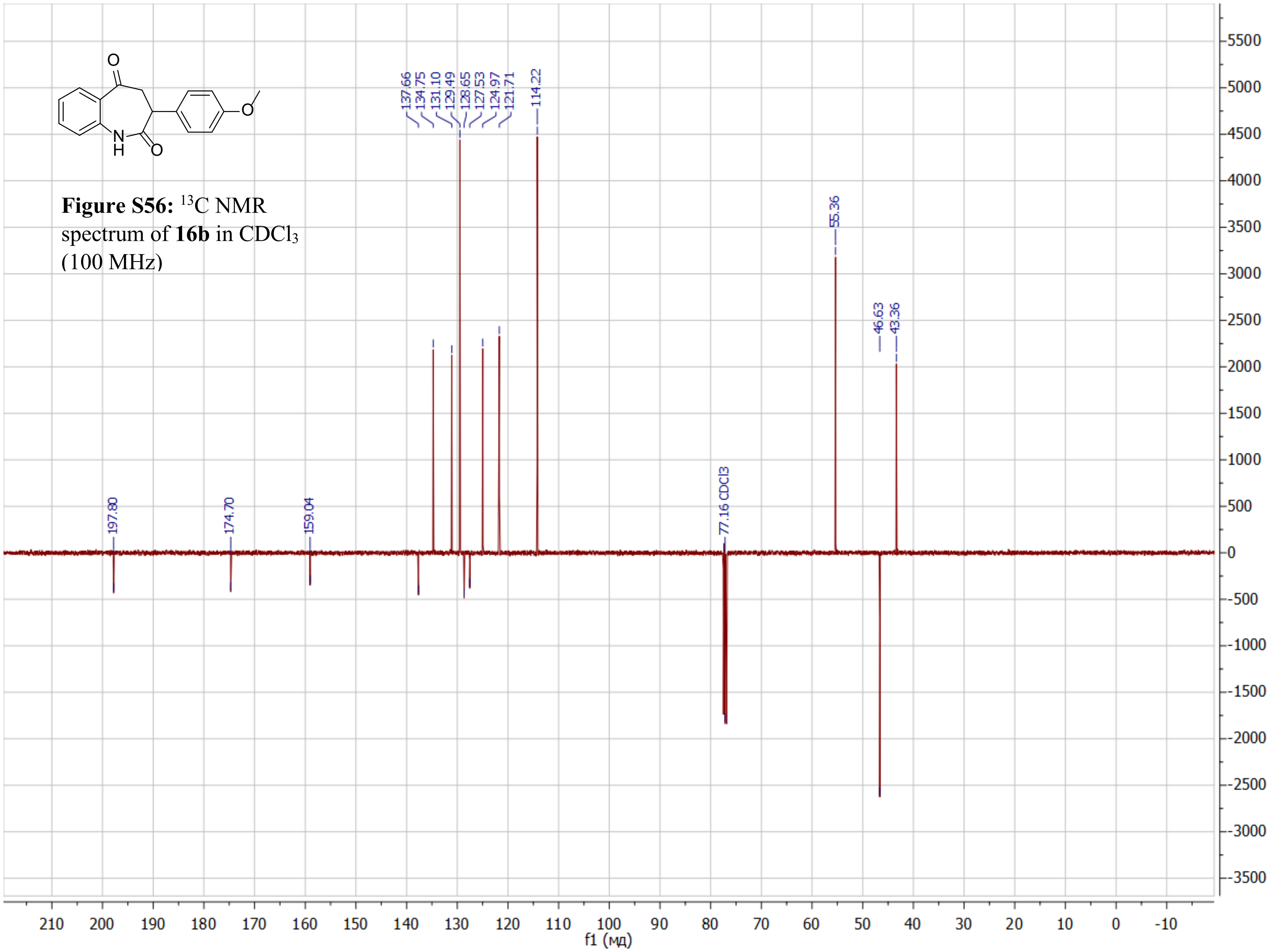

Figure S56: ^{13}C NMR spectrum of **16b** in $CDCl_3$ (100 MHz)

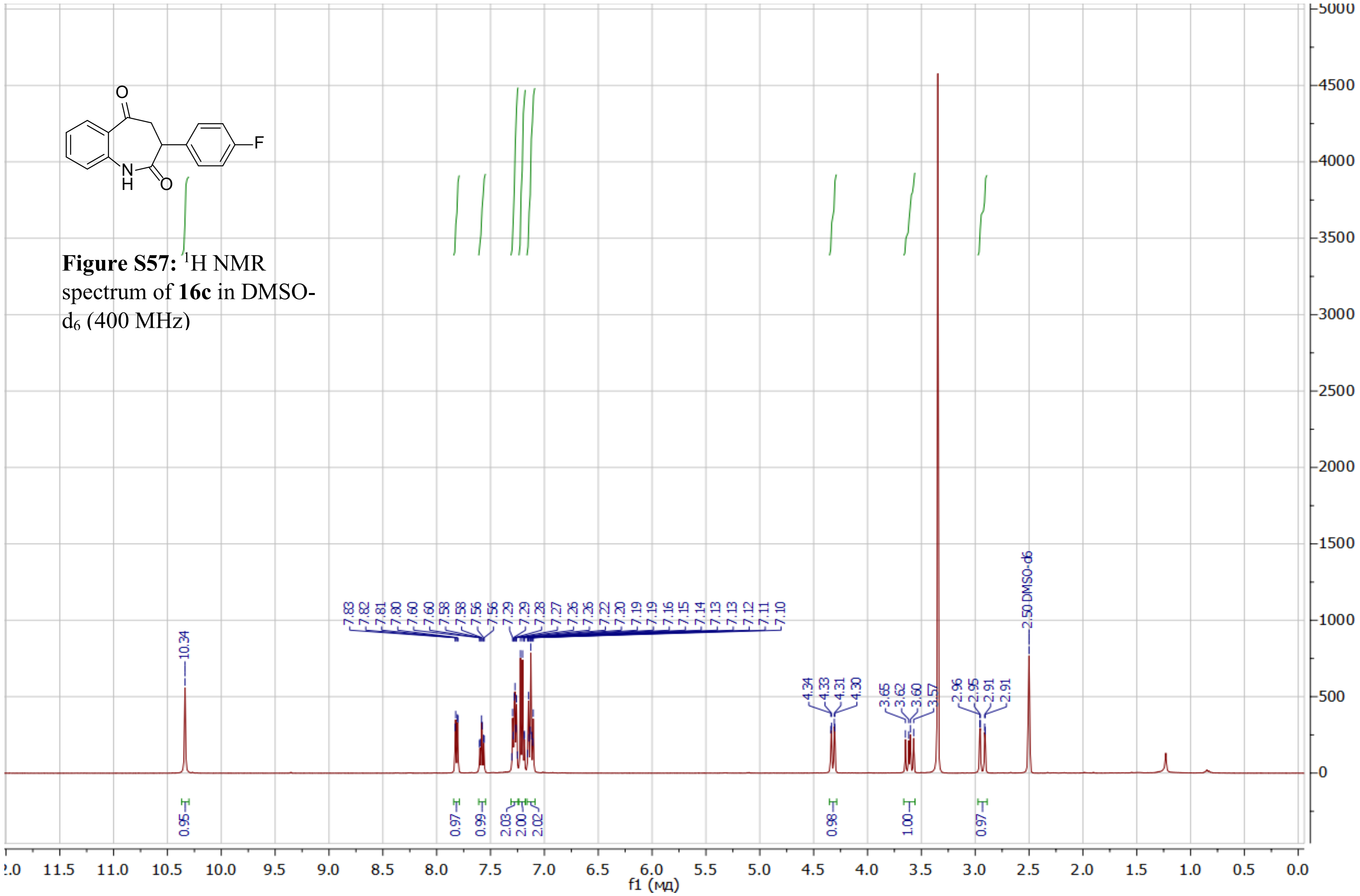

Figure S57: ^{1}H NMR spectrum of **16c** in DMSO-d_6 (400 MHz)

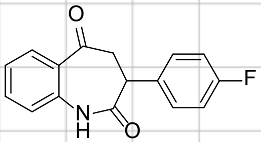

Figure S58: ^{13}C NMR spectrum of **16c** in DMSO-d_6 (100 MHz)

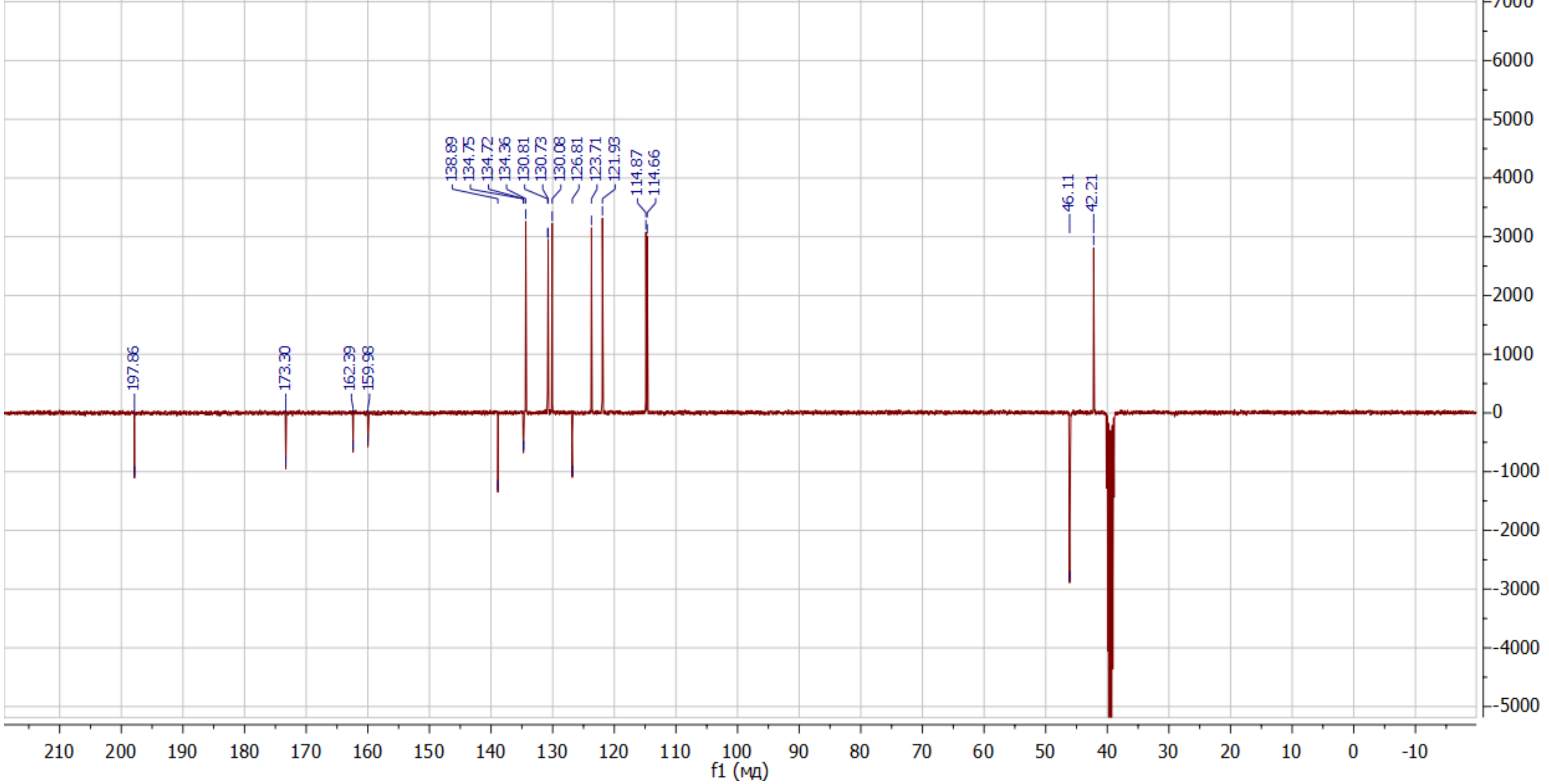

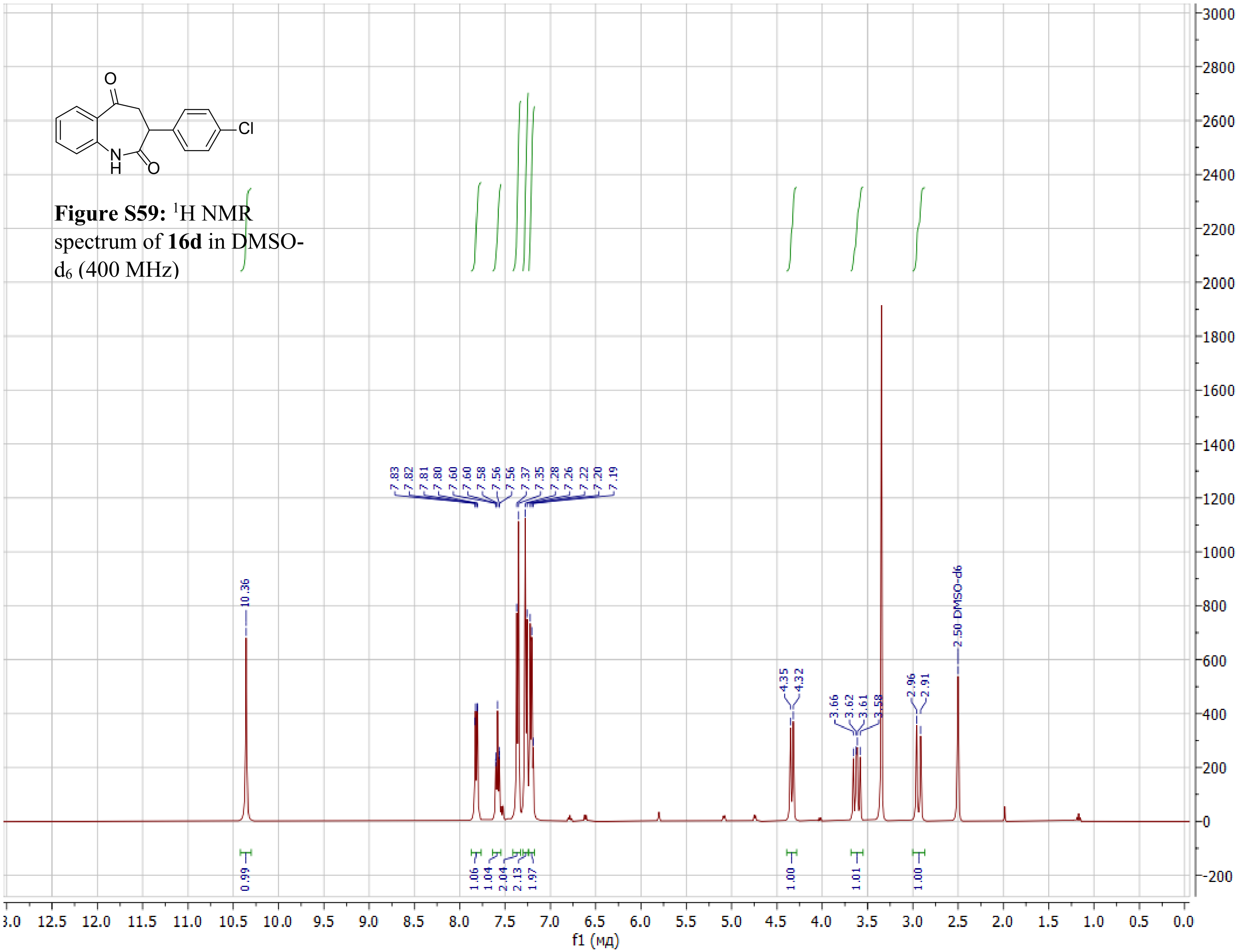

Figure S59: ^{1}H NMR spectrum of **16d** in DMSO-d_6 (400 MHz)

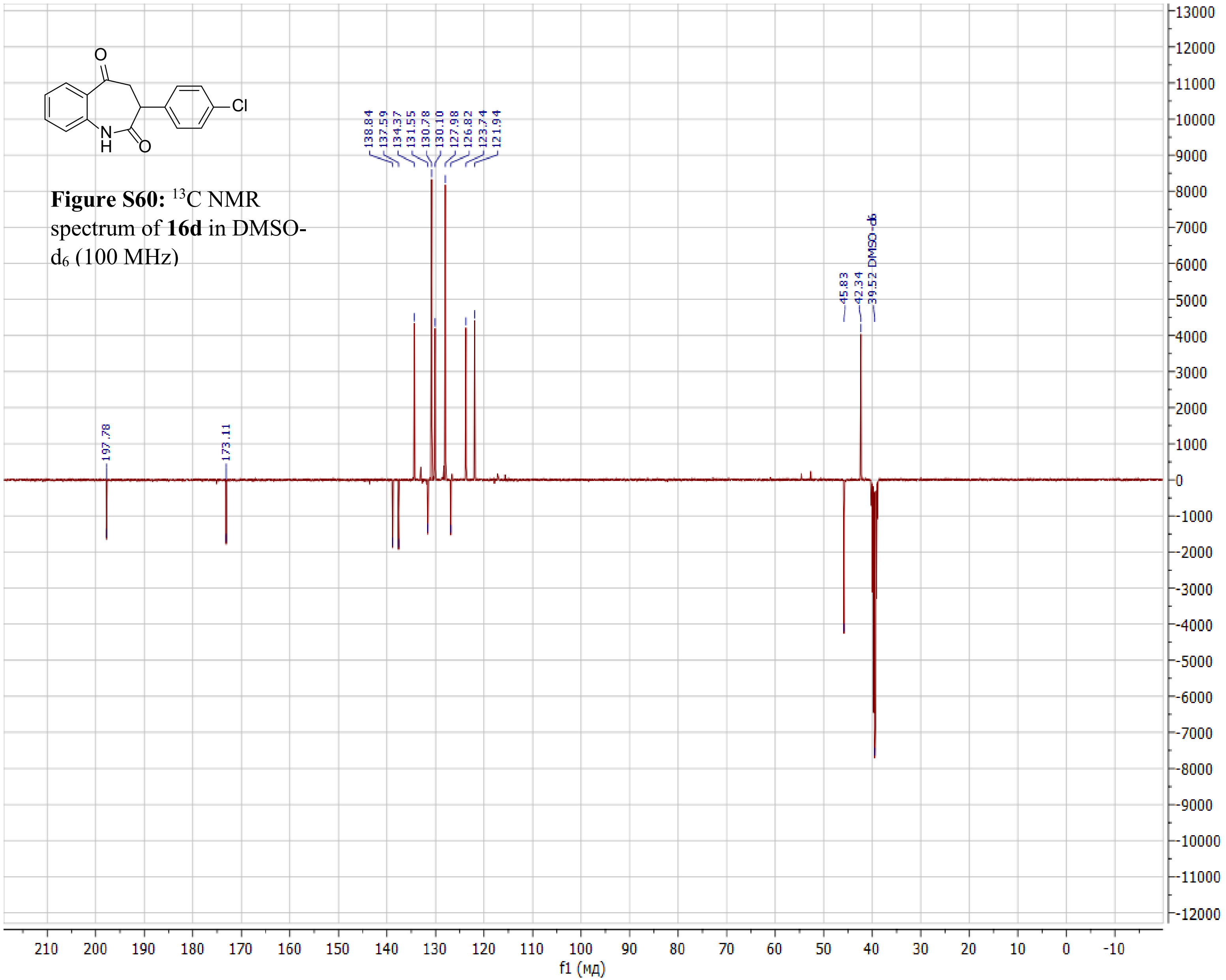

Figure S60: ^{13}C NMR spectrum of **16d** in DMSO-d_6 (100 MHz)

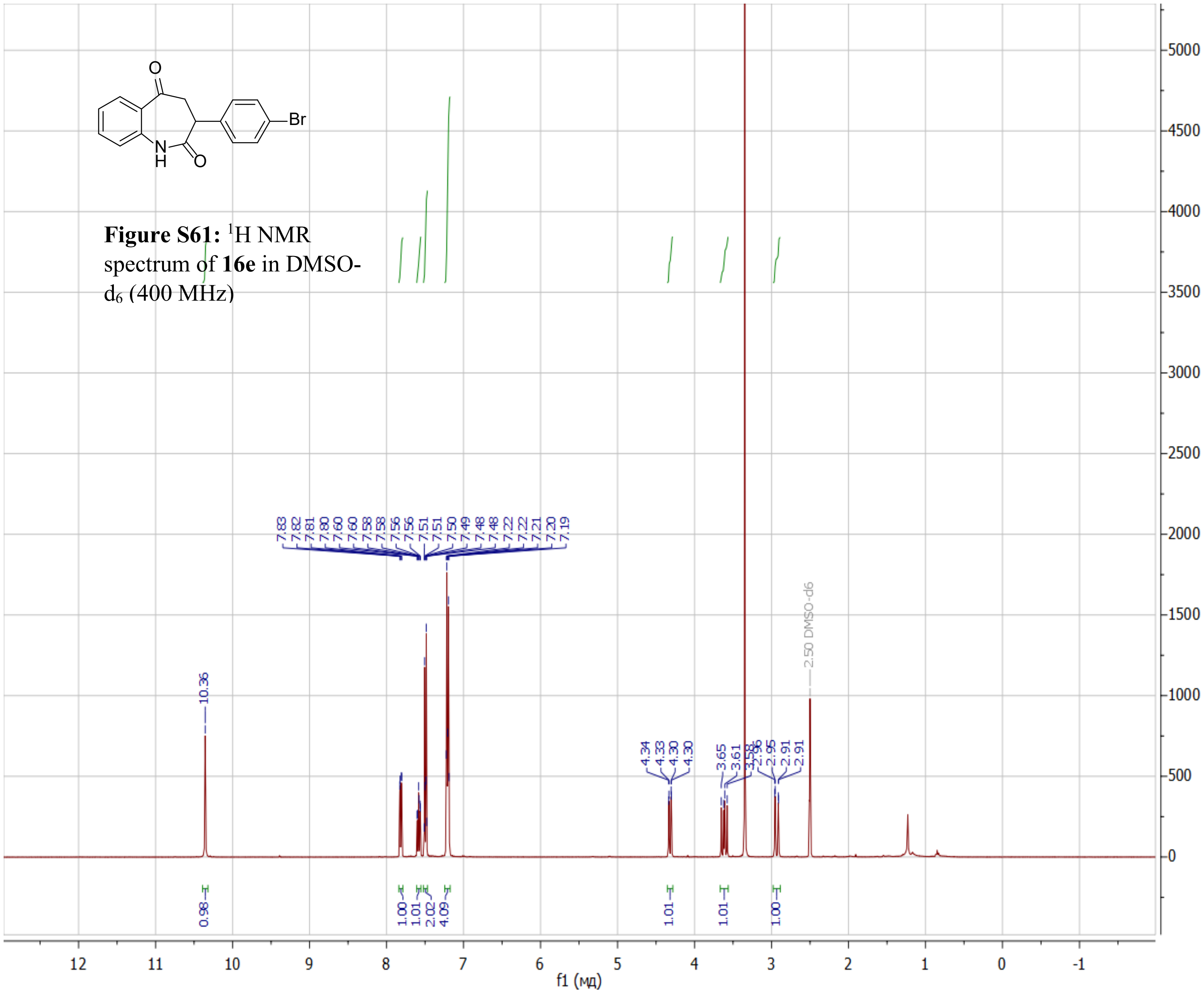

Figure S61: ^{1}H NMR spectrum of **16e** in DMSO-d_6 (400 MHz)

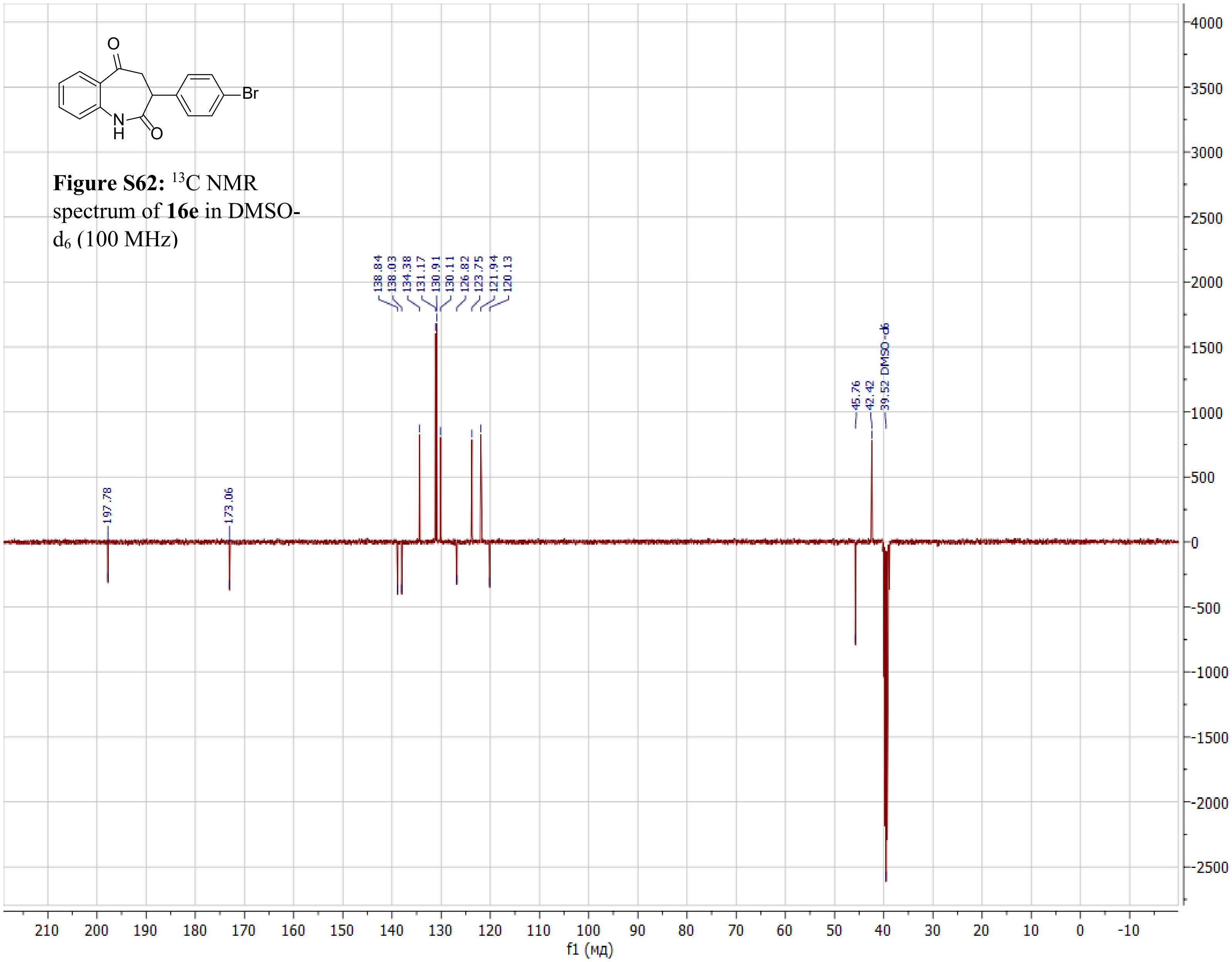

Figure S62: ^{13}C NMR spectrum of **16e** in DMSO-d_6 (100 MHz)

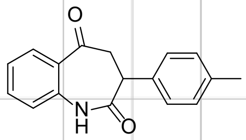

Figure S63: ^{1}H NMR spectrum of **16f** in $CDCl_3$ (400 MHz)

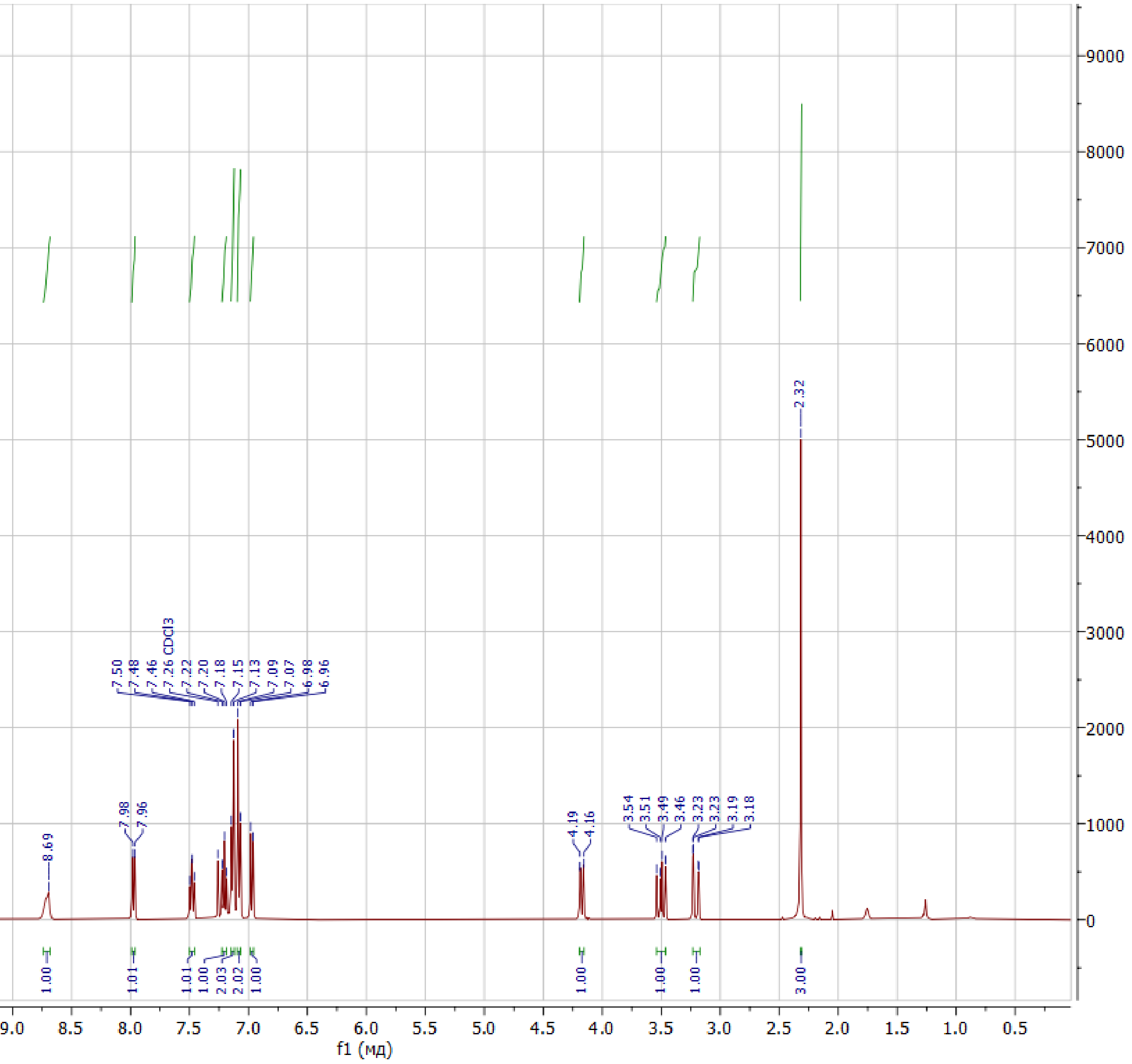

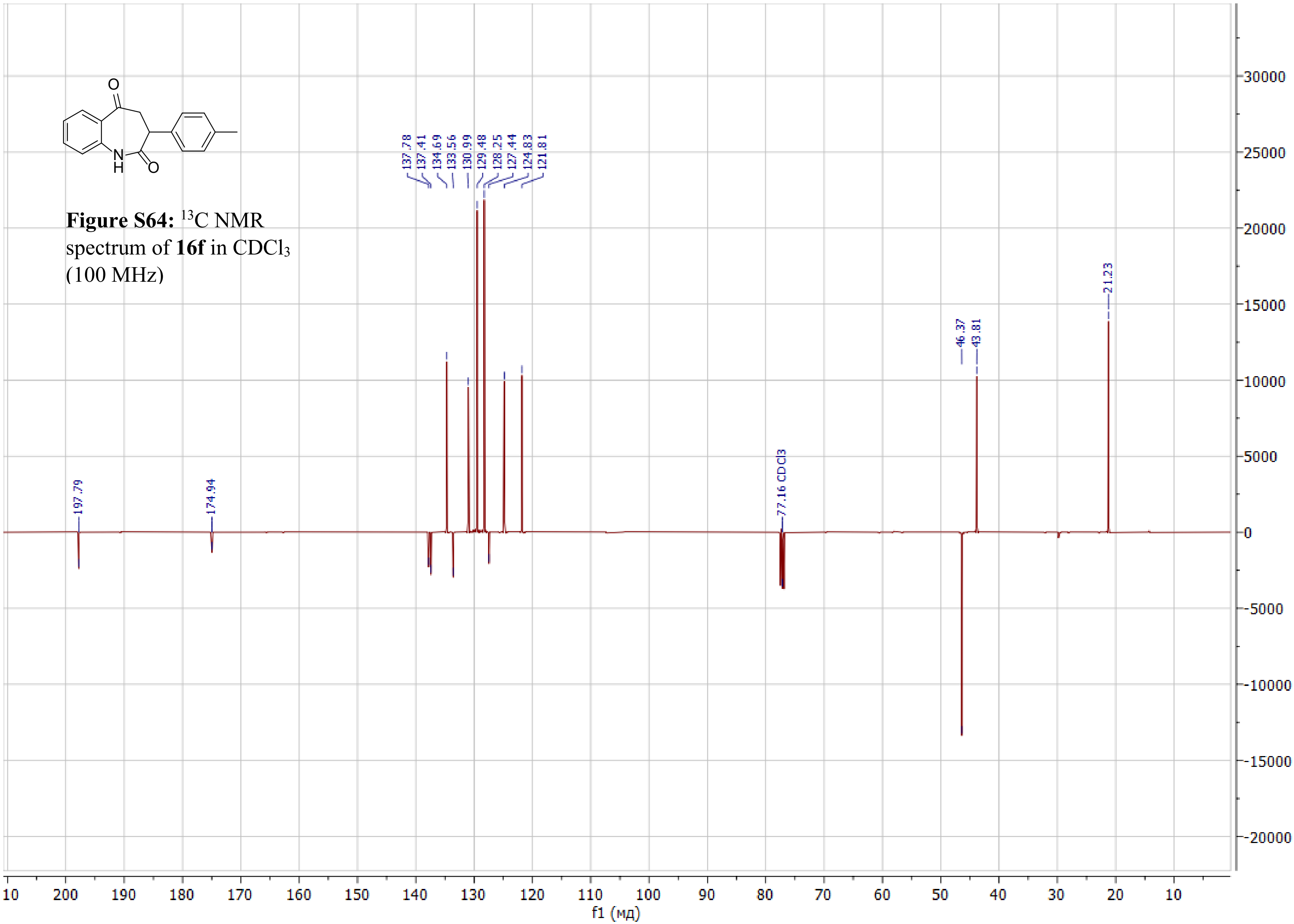

Figure S64: ^{13}C NMR spectrum of **16f** in $CDCl_3$ (100 MHz)

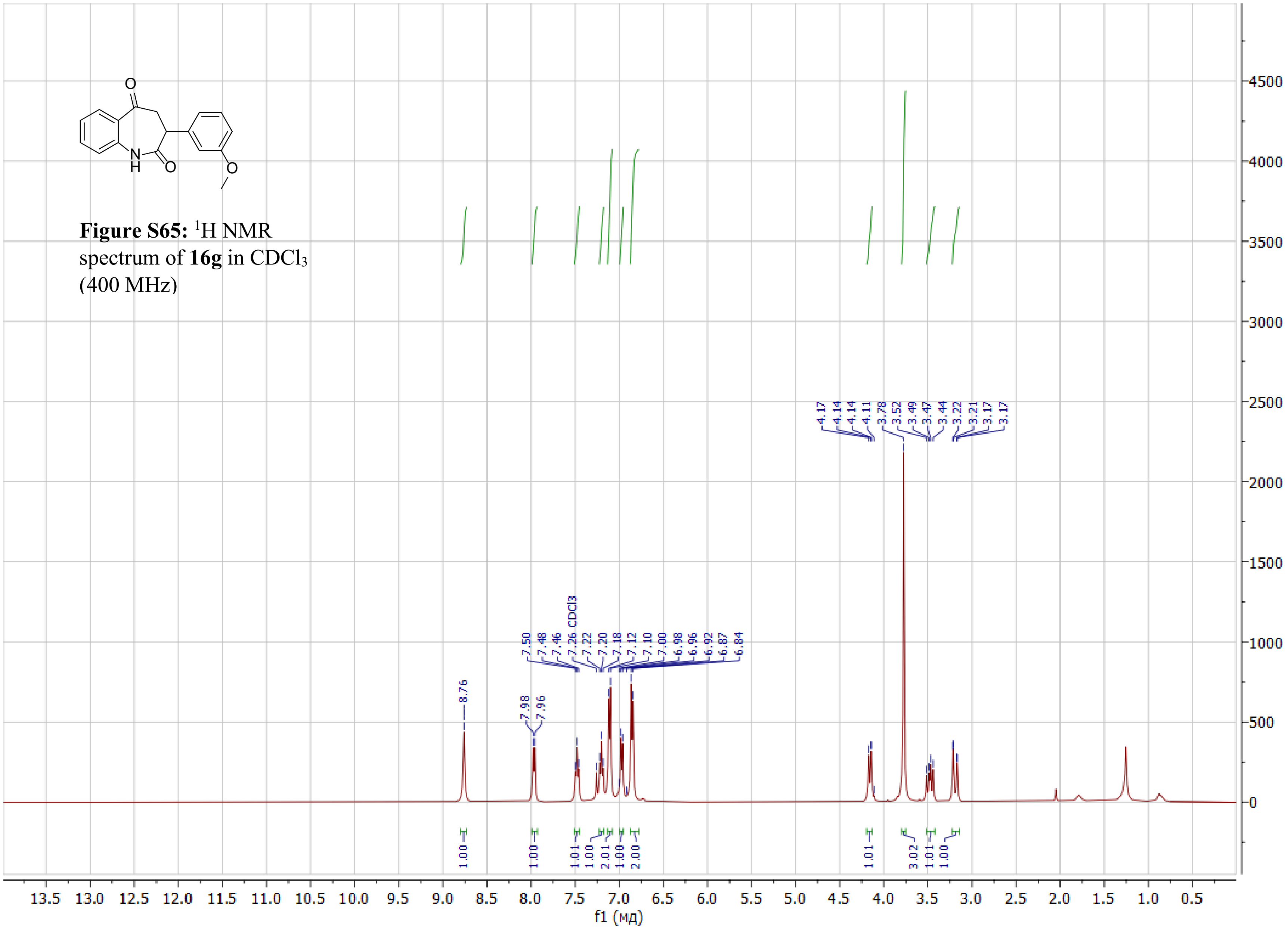

Figure S65: ^{1}H NMR spectrum of **16g** in $CDCl_3$ (400 MHz)

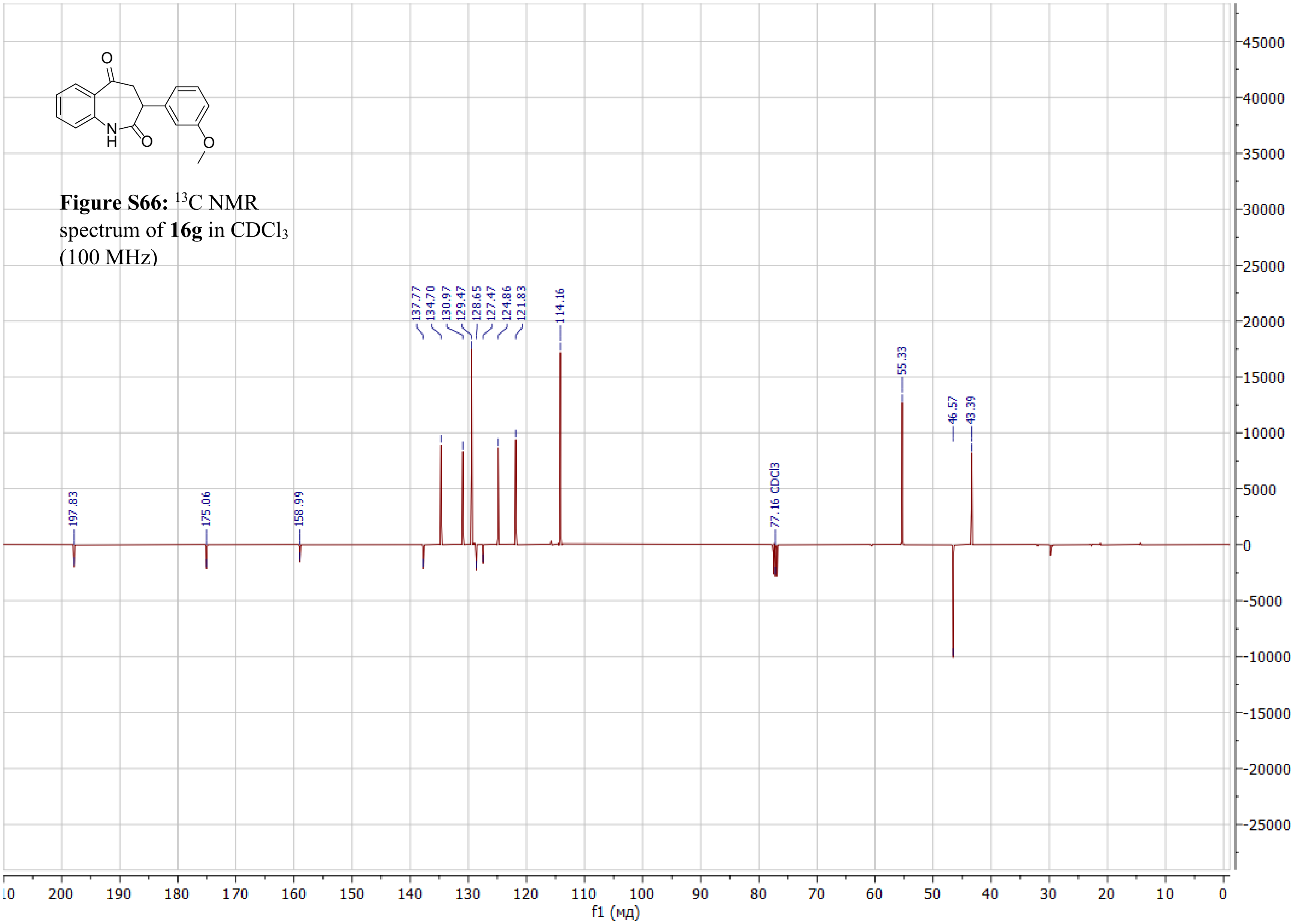

Figure S66: ^{13}C NMR spectrum of **16g** in $CDCl_3$ (100 MHz)

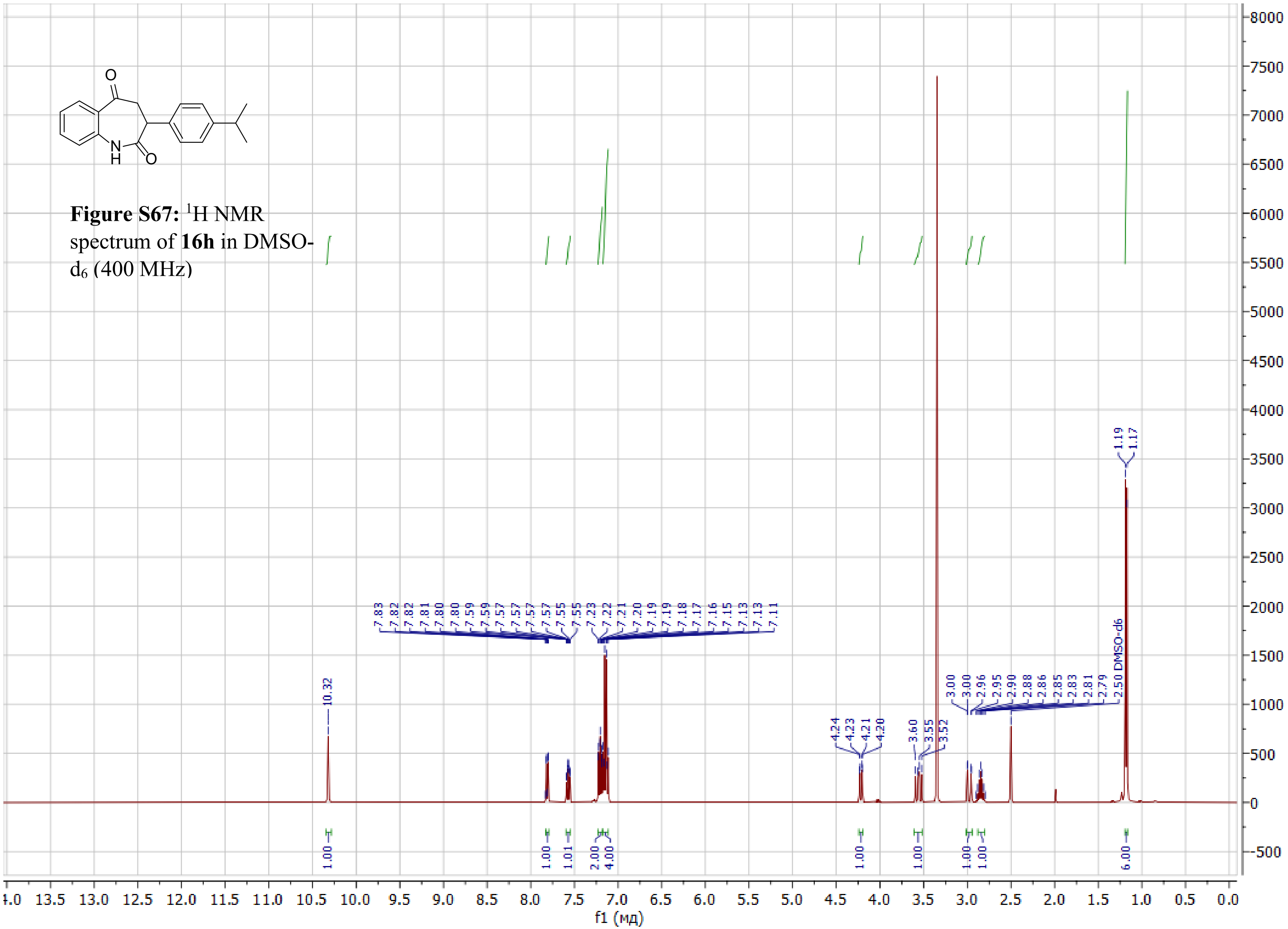

Figure S67: ^{1}H NMR spectrum of **16h** in DMSO-d$_6$ (400 MHz)

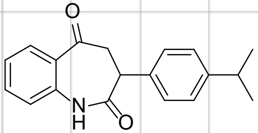

Figure S68: ^{13}C NMR spectrum of **16h** in DMSO-d_6 (100 MHz)

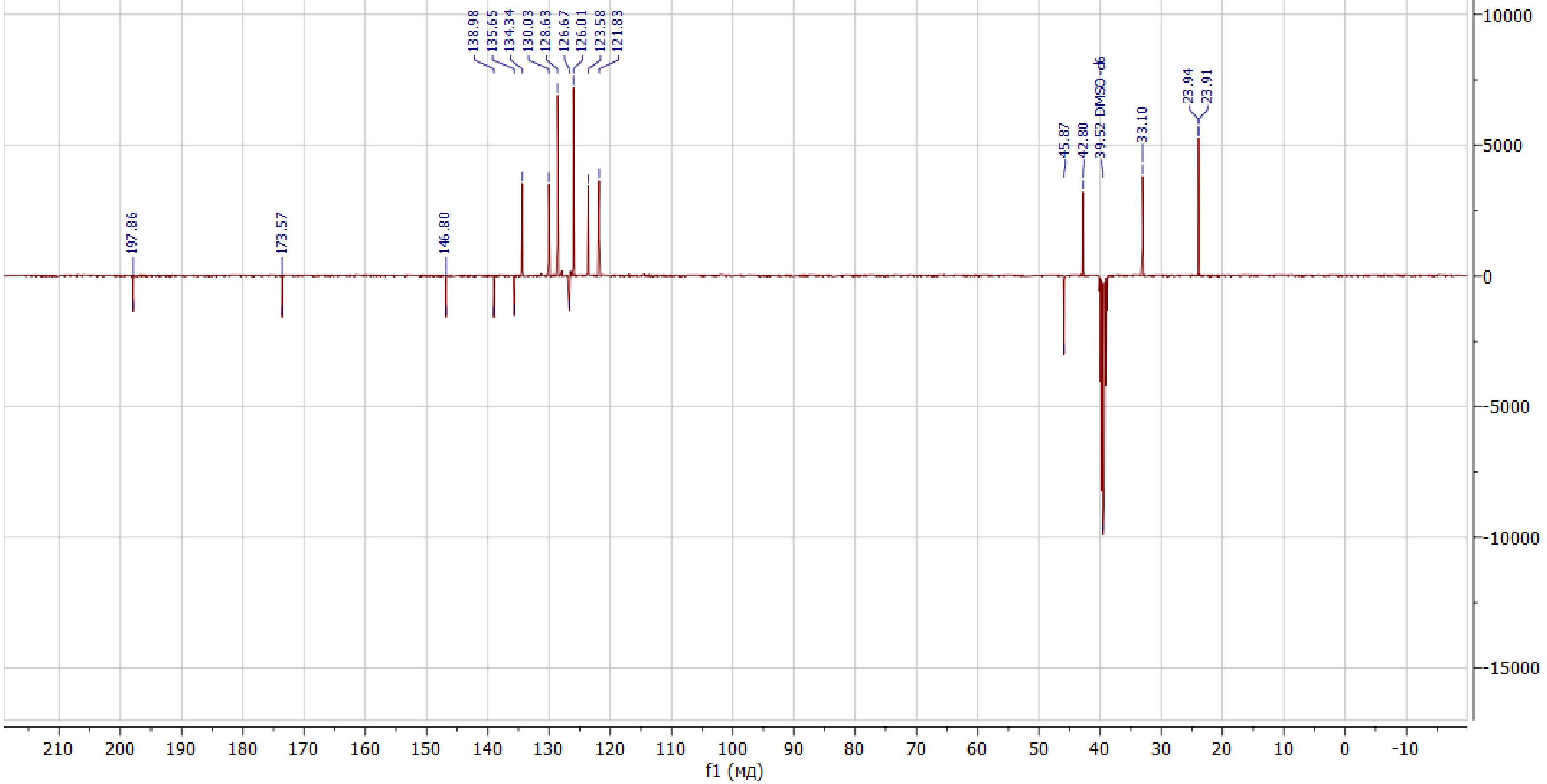

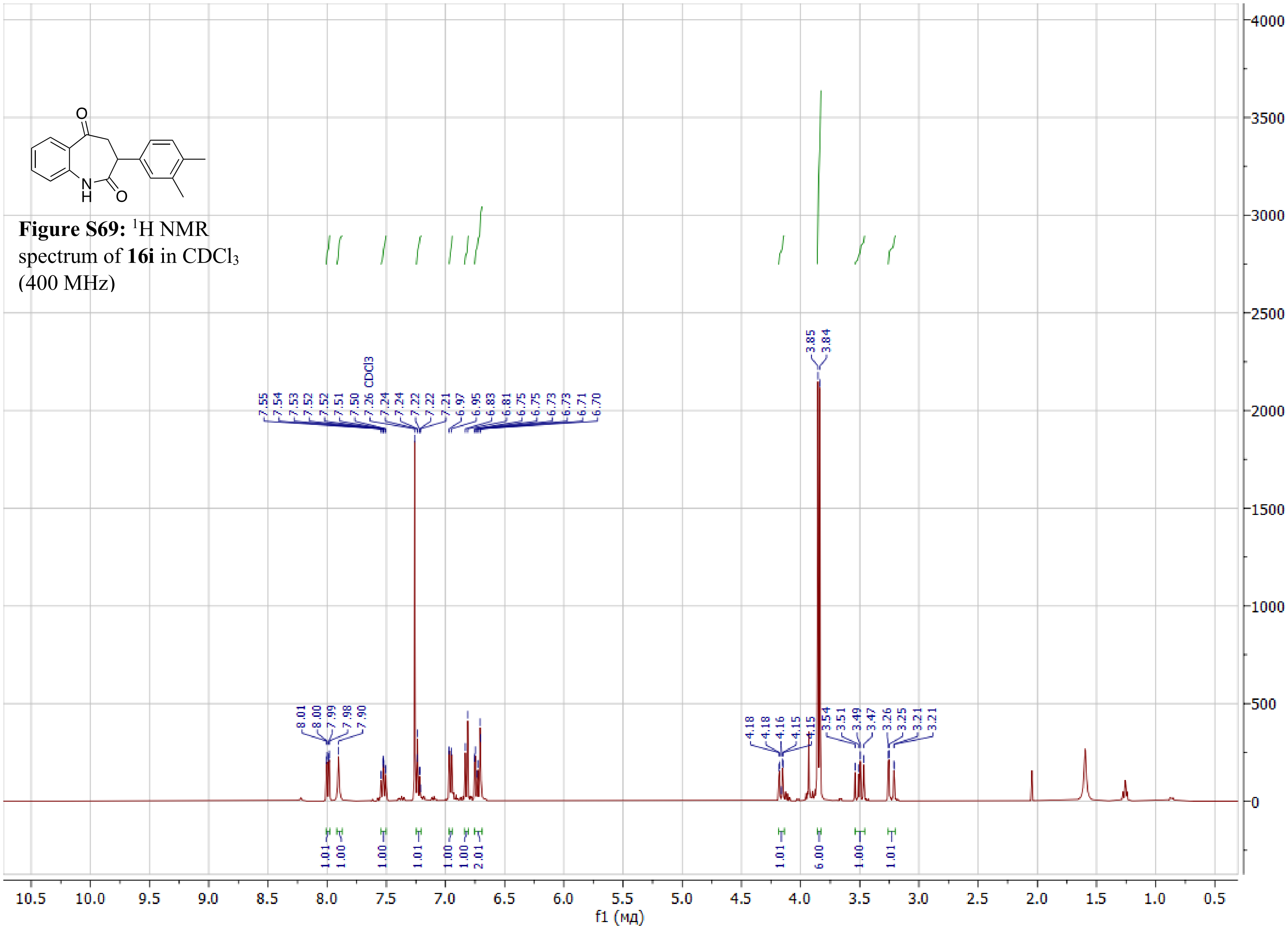

Figure S69: ^{1}H NMR spectrum of **16i** in $CDCl_3$ (400 MHz)

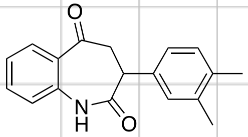

Figure S70: ^{13}C NMR spectrum of **16i** in $CDCl_3$ (100 MHz)

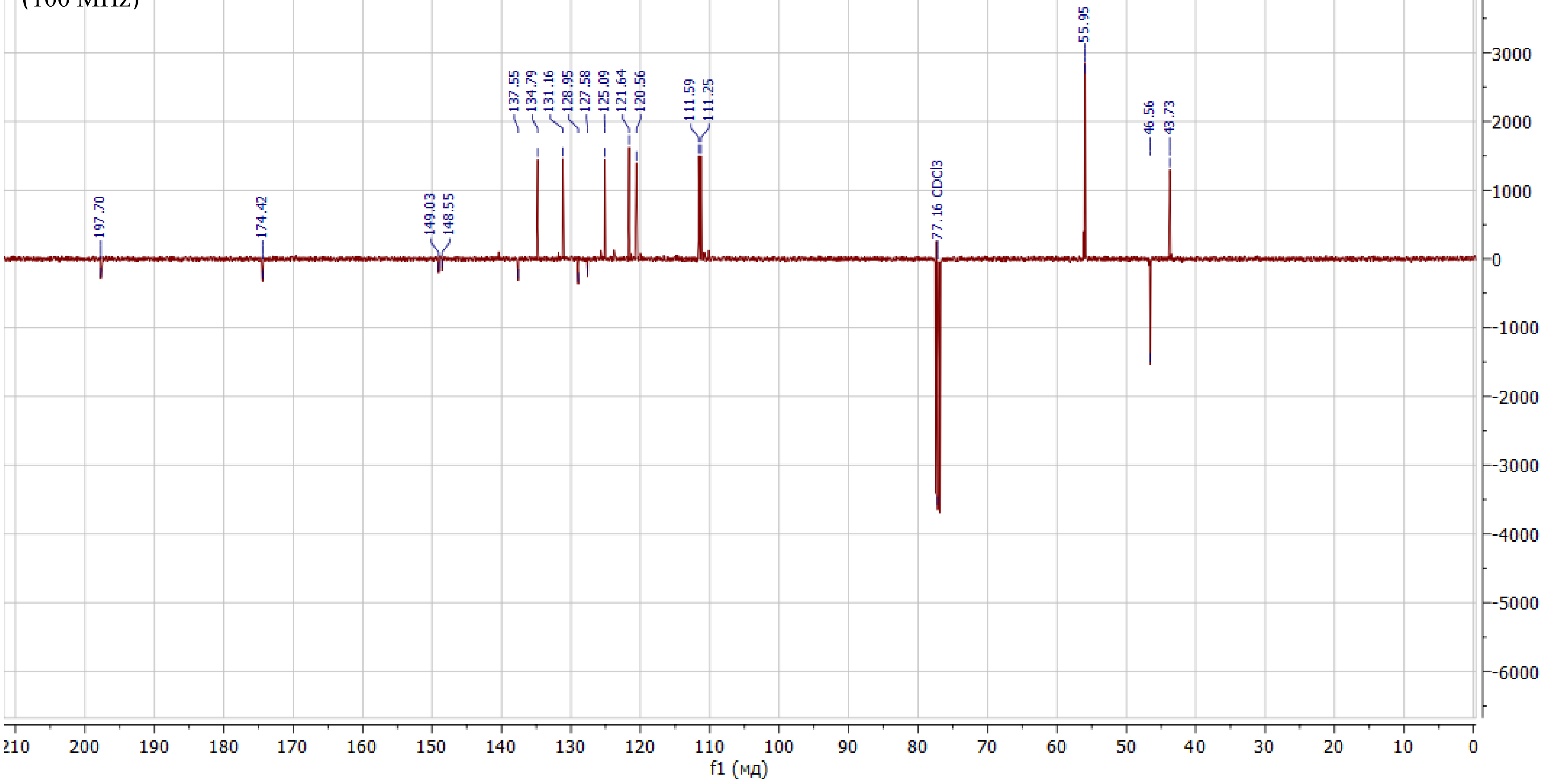

Figure S71: ^{1}H NMR spectrum of **16j** in $CDCl_3$ (400 MHz)

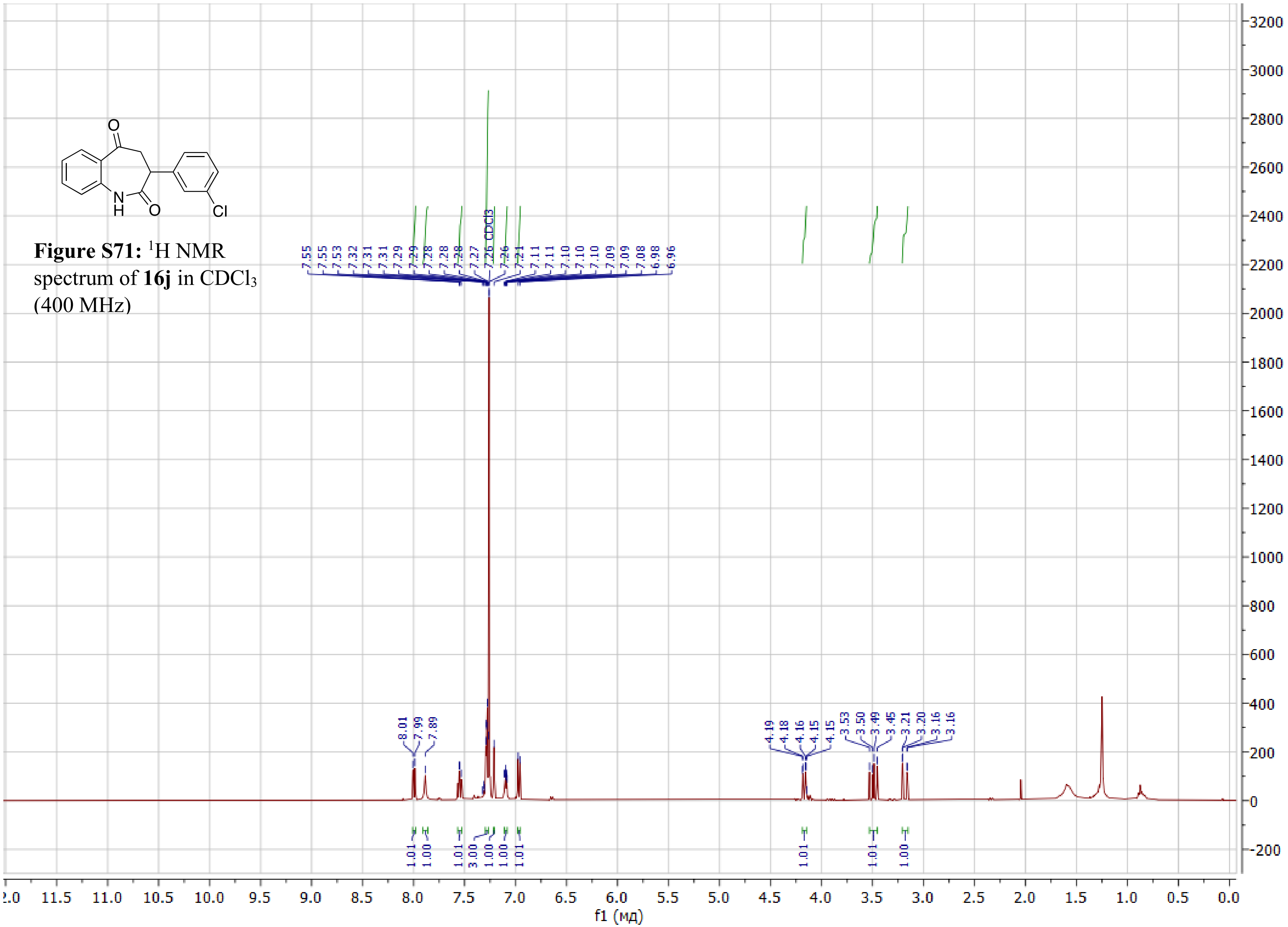

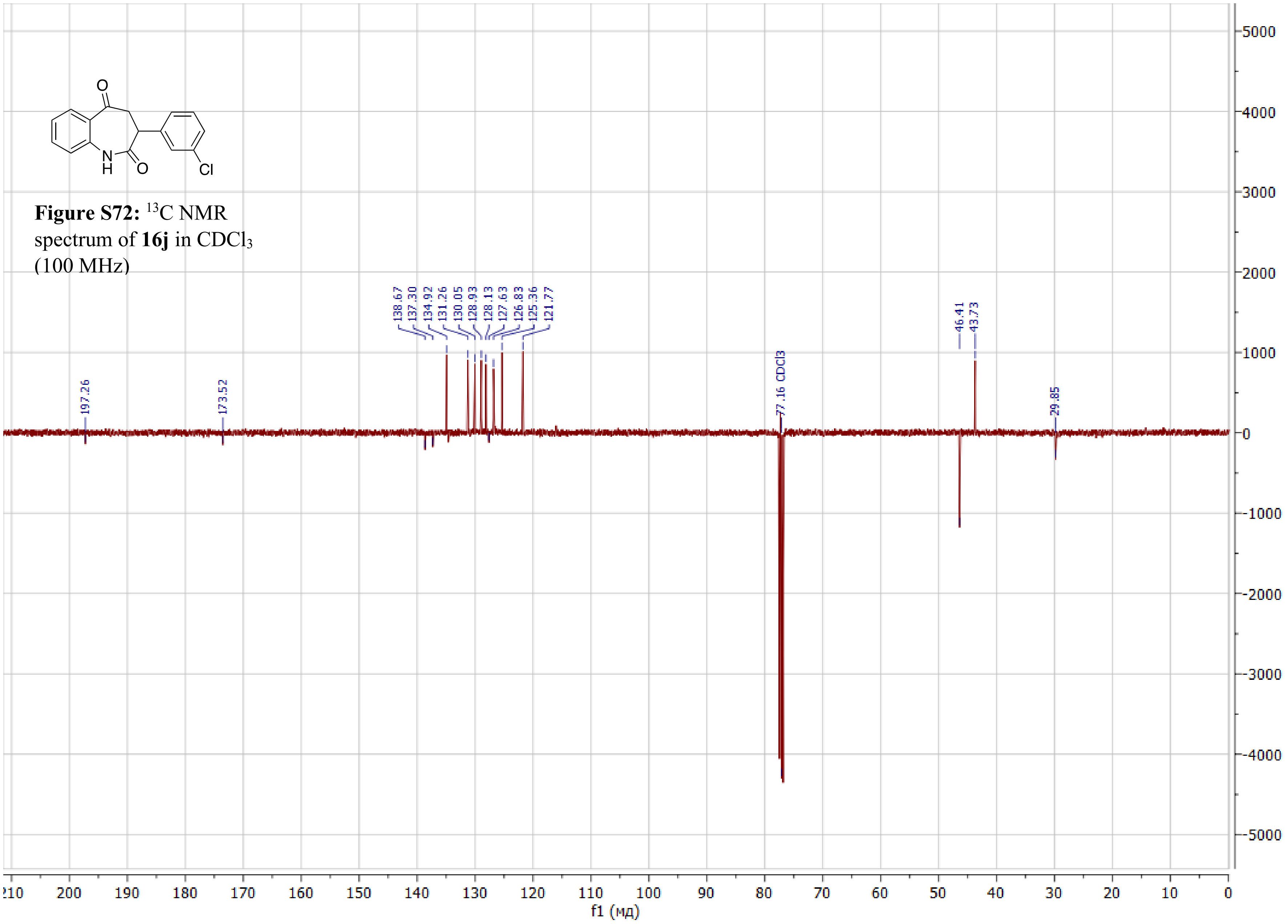

Figure S72: ^{13}C NMR spectrum of **16j** in $CDCl_3$ (100 MHz)

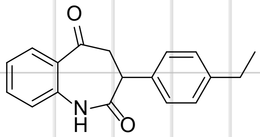

Figure S73: ^{1}H NMR spectrum of **16k** in DMSO-d_6 (400 MHz)

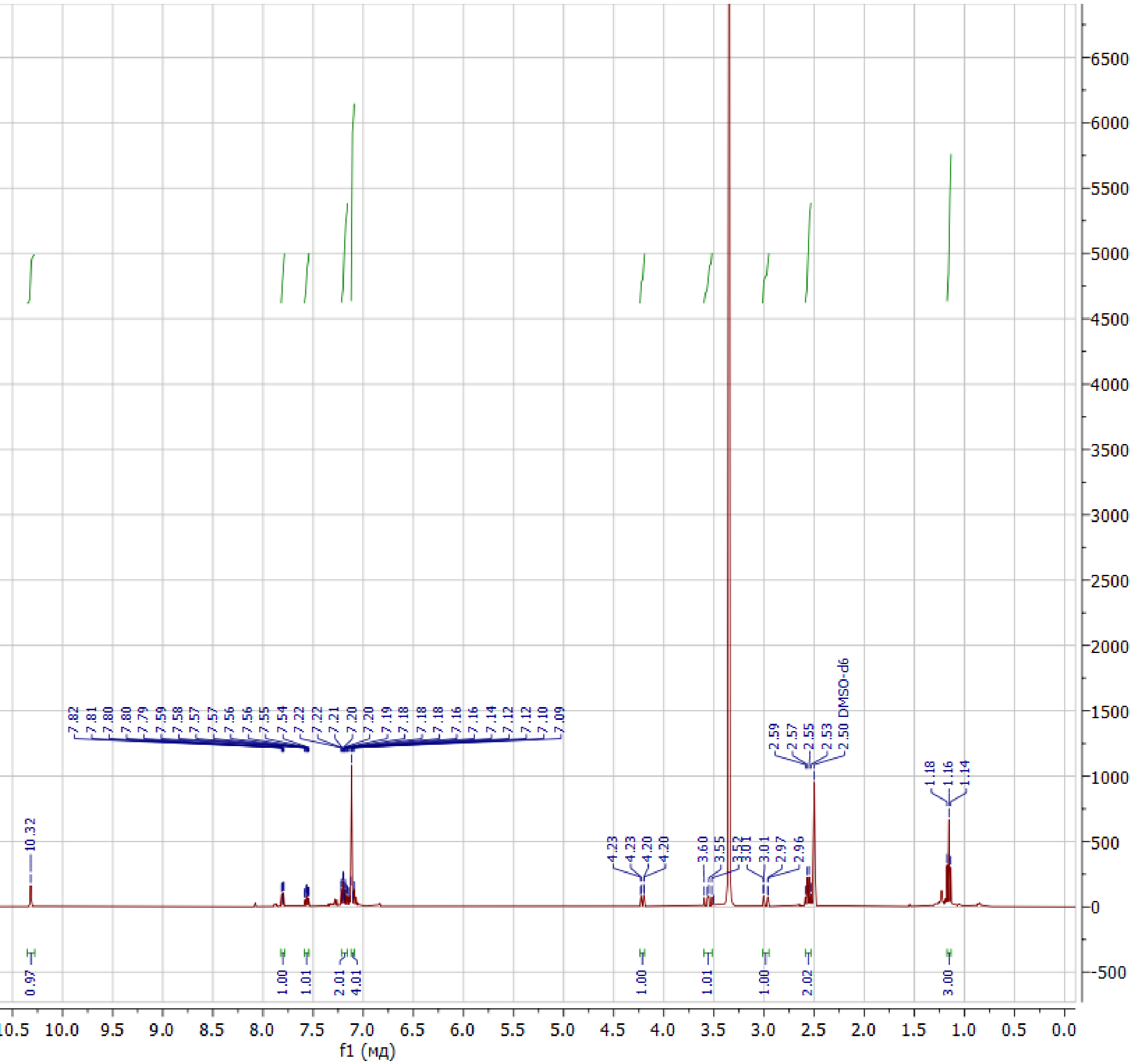

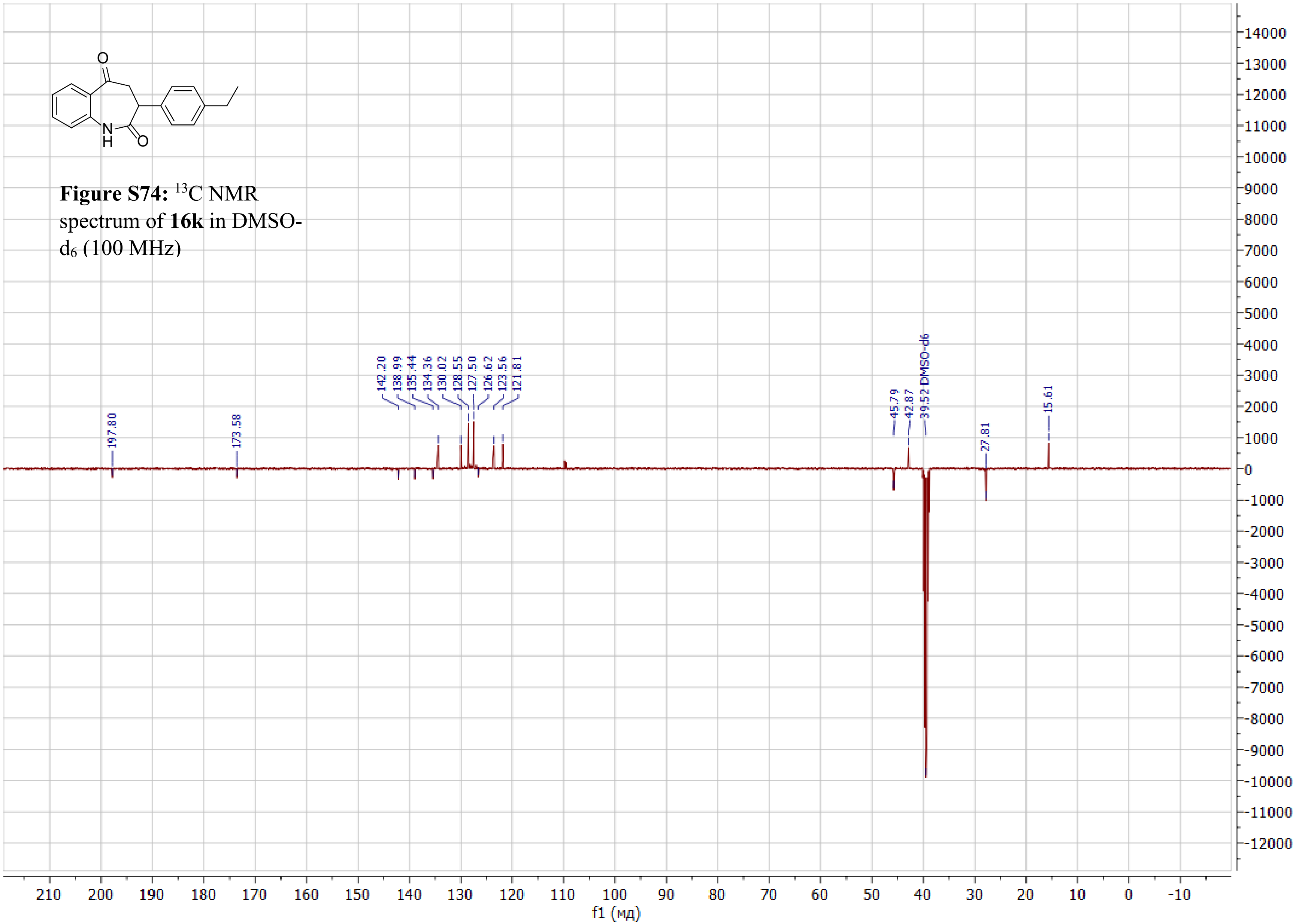

Figure S74: ^{13}C NMR spectrum of **16k** in DMSO-d_6 (100 MHz)
